# USP4 Auto-Deubiquitylation Promotes Homologous Recombination

**DOI:** 10.1016/j.molcel.2015.09.019

**Published:** 2015-11-05

**Authors:** Paul Wijnhoven, Rebecca Konietzny, Andrew N. Blackford, Jonathan Travers, Benedikt M. Kessler, Ryotaro Nishi, Stephen P. Jackson

**Affiliations:** 1 The Wellcome Trust/Cancer Research UK Gurdon Institute, University of Cambridge, Cambridge CB2 1QN, UK; 2 Target Discovery Institute, Nuffield Department of Medicine, University of Oxford, Oxford OX3 7FZ, UK; 3 The Wellcome Trust Sanger Institute, Hinxton CB10 1SA, UK

## Abstract

Repair of DNA double-strand breaks is crucial for maintaining genome integrity and is governed by post-translational modifications such as protein ubiquitylation. Here, we establish that the deubiquitylating enzyme USP4 promotes DNA-end resection and DNA repair by homologous recombination. We also report that USP4 interacts with CtIP and the MRE11-RAD50-NBS1 (MRN) complex and is required for CtIP recruitment to DNA damage sites. Furthermore, we show that USP4 is ubiquitylated on multiple sites including those on cysteine residues and that deubiquitylation of these sites requires USP4 catalytic activity and is required for USP4 to interact with CtIP/MRN and to promote CtIP recruitment and DNA repair. Lastly, we establish that regulation of interactor binding by ubiquitylation occurs more generally among USP-family enzymes. Our findings thus identify USP4 as a novel DNA repair regulator and invoke a model in which ubiquitin adducts regulate USP enzyme interactions and functions.

## Highlights

• The deubiquitylating enzyme USP4 promotes homologous recombination-mediated repair
• USP4 regulates recruitment and/or association dynamics of CtIP at DNA damage sites
• Auto-deubiquitylation of USP4 stimulates HR events and interactions with CtIP/MRN
• Ubiquitin adducts might regulate USP-enzyme interactions/function more commonly

## Graphical Abstract

**Figure:**
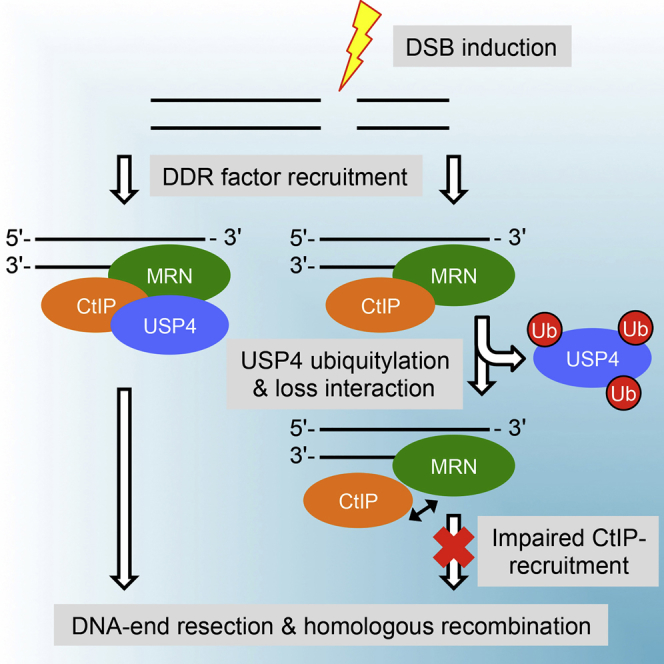

## Teaser

Wijnhoven et al. revealed that the human deubiquitylase USP4 promotes homologous recombination, DNA end-resection, and CtIP recruitment and that USP4 interacts with CtIP and MRN. Wijnhoven et al. also showed that such interactions and DDR processes are stimulated by USP4 auto-deubiquitylation on multiple sites, including those on cysteine residues.

## Introduction

To counteract the deleterious consequences of DNA double-strand breaks (DSBs) and other DNA lesions, multiple cellular mechanisms have evolved, collectively termed the DNA damage response (DDR) (Ciccia and Elledge, 2010, Jackson and Bartek, 2009). The DDR is tightly regulated by reversible post-translational protein modifications (PTMs). For instance, following DSB recognition by sensor proteins such as the MRE11-RAD50-NBS1 (MRN) complex that interacts with CtIP (Sartori et al., 2007), phosphorylation cascades triggered by the protein kinases ATM, ATR, and DNA-PKcs control and coordinate DSB repair and associated events. These events include phosphorylation of histone 2A variant H2AX on serine 139 (to form γH2AX) on chromatin flanking DSB sites, to which MDC1 then binds (Stucki et al., 2005), mediating recruitment of factors such as the E3 ubiquitin ligases RNF8 and RNF168, together with 53BP1 and the BRCA1-A complex, leading to chromatin remodelling in preparation for repair (Ciccia and Elledge, 2010).

Phosphorylation has been extensively studied in the context of the DDR for many years. By contrast, how ubiquitylation—the covalent attachment of the ∼8.5 kDa protein ubiquitin to substrates—and related events regulate DSB repair and associated processes has only recently become the focus of intensive research. Ubiquitin is conjugated to its substrates via an enzymatic cascade involving an activating (E1), conjugating (E2) enzyme, and, in most cases an E3 ubiquitin ligase (Hershko et al., 1983); and in mammals, ubiquitylation involves two E1s, more than 35 E2s, and >600 E3 ligases (Clague et al., 2015). Substrates can be mono-ubiquitylated at more than one site and/or are polyubiquitylated by polymerization of multiple ubiquitin moieties via one or more of seven internal lysine residues (Lys-6, Lys-11, Lys-27, Lys-29, Lys-33, Lys-48, and Lys-63) within ubiquitin or with the ubiquitin amino-terminus (Komander, 2009a). These different linkages lead to ubiquitin chains with distinct structural features and functions, including those with well-established roles in the DDR (Ciccia and Elledge, 2010, Jackson and Durocher, 2013). In recent years, it has become evident that editing and removal of such ubiquitylations by deubiquitylases (DUBs) play crucial roles in regulating ubiquitylation events and the activities they control.

The human genome encodes 94 putative DUBs, classed into five groups based on structural features of their catalytic domains (Komander et al., 2009b). We recently carried out a systematic screen of DUBs for DDR functions, particularly focusing on DSB repair by non-homologous end-joining (NHEJ) or homologous recombination (HR) (Nishi et al., 2014). Among others, this work suggested DSB repair roles for members of the structurally similar ubiquitin-specific proteases USP4, USP15, and USP11 (Komander et al., 2009b), the latter being a DUB with previously established DDR roles (Schoenfeld et al., 2004, Wiltshire et al., 2010). Notably, USP4, USP11, and USP15 are implicated in related cellular events, including TGF-β signaling (Al-Salihi et al., 2012, Inui et al., 2011, Zhang et al., 2012), raising the possibility that they might have redundant and/or complementary DDR functions.

Here, we report that USP4, a DUB with previously reported links to mitogen-activated protein (MAP) kinase signaling (e.g., Zhang et al., 2012), pre-mRNA splicing (Song et al., 2010), and control of p53 stability (Zhang et al., 2011), regulates DNA repair and cellular survival upon DSB induction. We also show that USP4 depletion impairs HR repair by affecting the process of DNA-end resection. Additionally, we establish that USP4 interacts with CtIP and the MRN complex via its C-terminal insert domain and that these interactions are subject to a USP4 auto-regulatory deubiquitylation mechanism. Finally, we provide evidence that this type of control might operate more widely by showing that interactions between USP15 with SMAD2/3 (Inui et al., 2011) are subject to USP15 auto-regulation.

## Results

### USP4 Promotes DSB Repair

**Figure 1:**
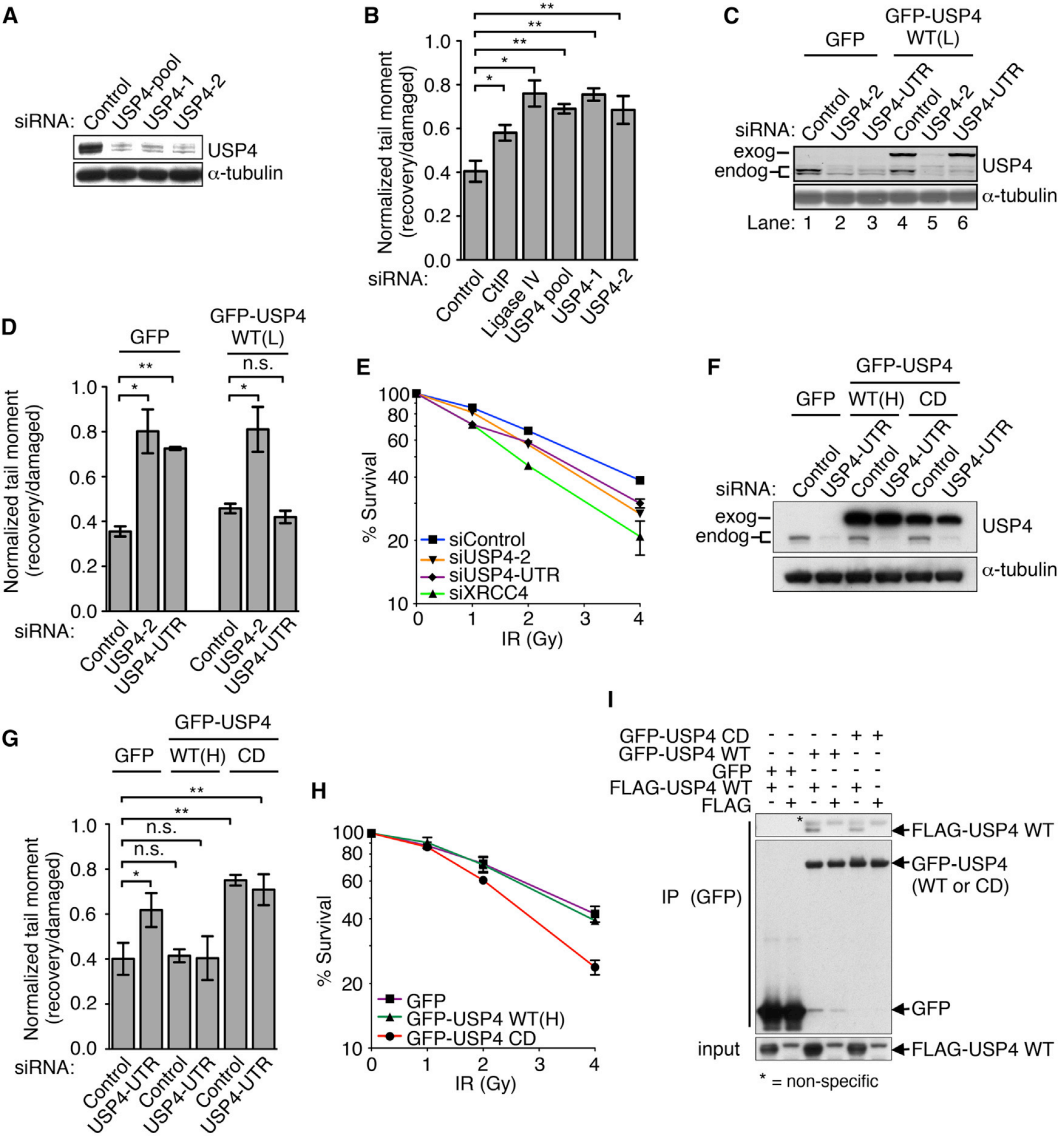
USP4 Promotes DSB Repair (A) USP4-targeting siRNAs depleted USP4 from U2OS cells. (B) USP4 depletion from U2OS cells caused DSB repair defects (neutral comet assays) after phleomycin (40 μg/ml, 2 hr) treatment, measuring the effects of depleting CtIP or DNA ligase IV, as controls (mean ± SEM; n = 3). Also see Table S1 for siRNAs and Table S2 for antibodies used in this study. (C) Treatments of U2OS cells with USP4-2 but not USP4-UTR siRNAs depleted exogenously expressed GFP-USP4 WT(L); exog, exogenous; endog, endogenous. Also, see Table S3 for plasmids used in this study. (D) Exogenously expressed USP4 WT(L) restored DSB repair defects (neutral comet assays) observed after USP4 depletion (mean ± SEM; n = 3). (E) USP4 depletion sensitized U2OS cells to IR (mean ± SEM; n = 3 and XRCC4 siRNA-treated cells were the positive control). (F) Treatment of U2OS cells with USP4-UTR siRNAs depleted endogenous USP4 but not exogenously expressed GFP-USP4 WT(H) or CD. exog, exogenous; endog, endogenous (also see Figure S1C for expression levels). (G) Expression of GFP-USP4 CD but not WT(H) caused DSB repair defects (neutral comet assays) irrespective of endogenous USP4 depletion (mean ± SEM; n = 3). (H) Expression of GFP-USP4 CD sensitized U2OS cells to IR (mean ± SEM; n = 3). (I) GFP-USP4 WT and CD immunoprecipitations from U2OS cell extracts retrieved FLAG-USP4 WT (^∗^p < 0.05 ^∗∗^p < 0.01; n.s., not significant). See also Figure S1.

We recently showed that the structurally similar proteins USP4, USP11, and USP15 have DDR roles (Nishi et al., 2014) (see Figure S1A for their domain features). Focusing on USP4, we found by neutral comet assays that its depletion from human U2OS cells by various short-interfering RNA (siRNA) oligonucleotides (Figure 1A) reduced repair of DSBs induced by the radiomimetic chemical phleomycin (Figure 1B; see Figure S1B for control CtIP and DNA ligase IV depletions). To determine whether such effects were mediated via USP4 loss, we generated stable cell lines expressing GFP fused to wild-type USP4 (GFP-USP4 WT) and selected a clone, GFP-USP4 WT(L) (see Figure S1C for this and other cell lines used in this study), where fusion protein expression was comparable to endogenous USP4 (see Figure 1C, lane 4 to compare endogenous and exogenous USP4). We then treated GFP-USP4 WT(L) cells or control cells expressing GFP alone with an siRNA targeting the USP4 coding region (USP4-2) to deplete both endogenous and GFP-fused USP4 or with an siRNA targeting the USP4 3′ UTR to deplete endogenous but not exogenous GFP-fused USP4 (Figure 1C). Ensuing comet analyses (Figure 1D) revealed that, while both siRNAs produced DSB repair defects in control cells, only the coding-region targeting siRNA yielded such a defect in cells expressing GFP-USP4 WT(L). These data thus indicated that GFP-USP4 WT expression complemented DSB repair defects induced by depleting endogenous USP4. USP4 depletion also sensitized cells to ionizing radiation (IR) (Figure 1E; see Figure S1D for depletion of the NHEJ protein XRCC4), providing additional evidence that USP4 promotes DNA repair.

Consistent with previous work (Nishi et al., 2014), we found that stably expressed GFP-fused USP4 WT accumulated at DNA damage sites generated by laser micro-irradiation (Figure S1E), suggesting that USP4 functions in proximity to DNA lesions. By contrast, we did not detect DNA-damage accumulation of GFP-fused U4/U6 recycling protein SART3 (data not shown), which interacts with USP4 and targets USP4 to its spliceosomal substrate PRP3 (Song et al., 2010). Although this might be due to detection limitations, these data suggested that USP4 might exist in multiple complexes and that its roles in DSB repair might be distinct from its spliceosomal functions. While it has been reported that overexpressed SART3 causes nuclear localization of exogenously expressed USP4 (Song et al., 2010), we found that SART3 depletion from U2OS cells did not detectably affect the nuclear/cytoplasmic distribution of endogenous USP4 (data not shown), suggesting that USP4 nuclear targeting might be mediated by multiple mechanisms.

To assess whether USP4 catalytic activity was needed for effective DSB repair, we characterized U2OS cell clones expressing similar amounts of USP4 WT (referred to as GFP-USP4 WT(H)) or GFP fused to catalytically dead USP4 (USP4 CD), where the catalytic cysteine (Cys-311) was changed to alanine (C311A; also see Figure S1C for expression levels). We then depleted endogenous USP4 from such cells by siRNA treatment (Figure 1F) and subjected them to neutral comet assays. This showed that USP4 catalytic activity was required for effective DSB repair (Figure 1G). Furthermore, during the course of these studies, we found that cells expressing USP4 CD displayed DSB repair defects even without endogenous USP4 depletion (Figure 1G; see Figure S1F for equivalent neutral comet assays without siRNA treatments), suggesting that catalytically dead USP4 behaved in a dominant-negative manner. Accordingly, U2OS cells expressing GFP-USP4 CD were more sensitive to IR than cells expressing GFP or GFP-USP4 WT (Figure 1H). By co-transfection of FLAG-epitope-tagged USP4 WT with GFP-USP4 WT or CD constructs, followed by GFP-immunoprecipitation (IP) and western blot analyses, we found that USP4 molecules interacted with one another irrespective of catalytic function (Figure 1I). We thus speculate that USP4 CD exerts its dominant-negative effects, at least in part via binding to endogenous USP4.

### USP4 Functions in DNA-End Resection

**Figure 2:**
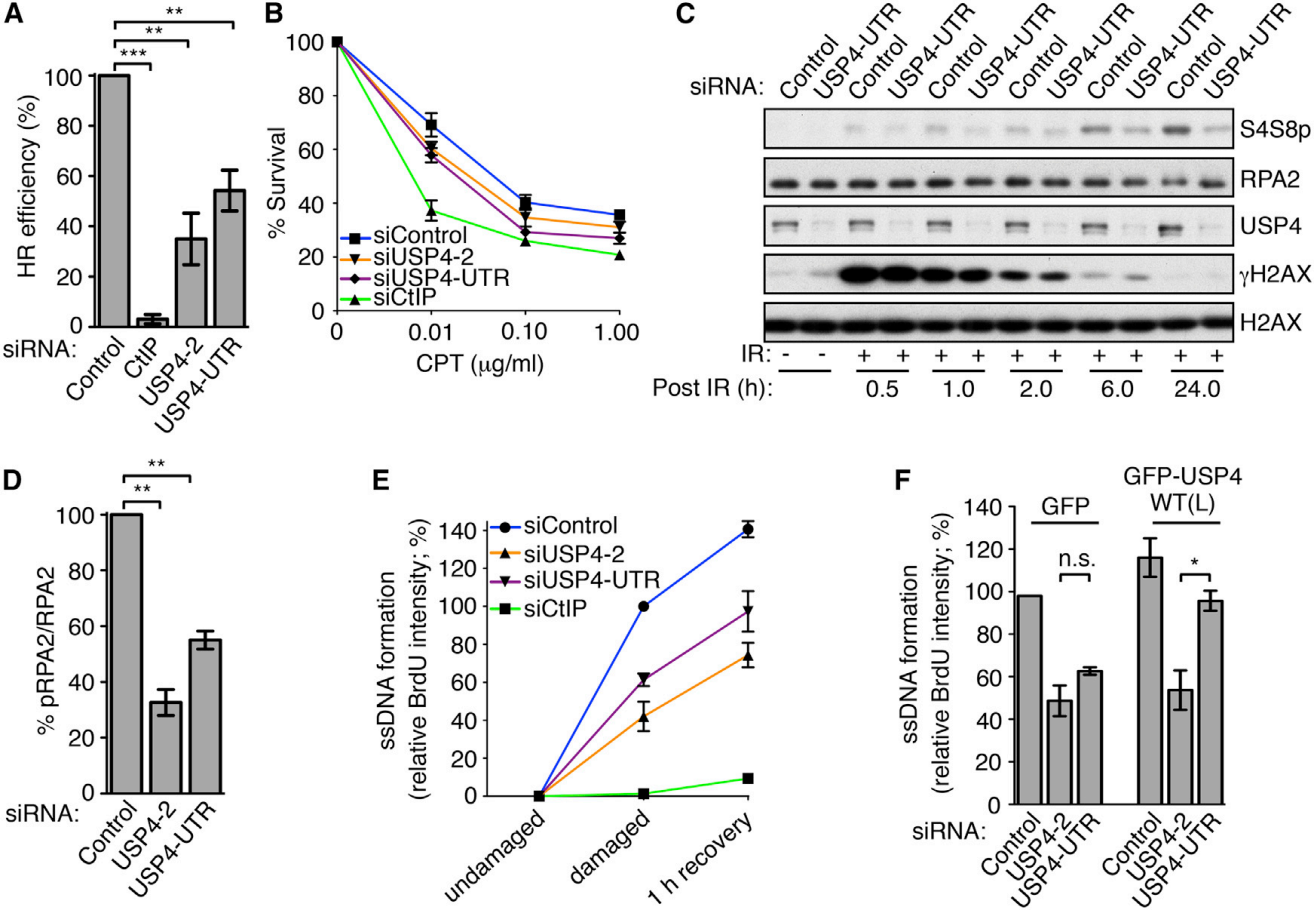
USP4 Functions in DNA-End Resection (A) USP4 depletion caused HR defects [direct-repeat (DR)-GFP reporter assays]. Quantifications were normalized to control siRNA-treated cells and set to 100% (mean ± SEM; n = 4). (B) USP4 depletion sensitized U2OS cells to camptothecin (CPT) (mean ± SEM; n = 3 and CtIP siRNA-treatment was the positive control). (C) USP4 depletion reduced RPA2 Ser-4/Ser-8 phosphorylation (S4S8p) after IR (10 Gy). (D) USP4 depletion reduced RPA2 S4S8p after camptothecin (1 μM, 1 hr) treatment. Intensities were quantified with Odysey CLx (LI-COR) and Image Studio 4.x software and RPA2 S4S8p was normalized to RPA2. Quantifications were normalized to the camptothecin-treated siControl and set to 100% (mean ± SEM; n = 3). (E) USP4 siRNA treatment, followed by camptothecin (1 μM, 1 hr) treatment, of U2OS cells reduced resection (BrdU intensities). Quantifications were normalized to the camptothecin-treated siControl (CtIP depletion was the positive control; mean ± SEM; n = 3). (F) GFP-USP4 WT(L)-complemented U2OS cells restored resection defects (BrdU intensities) observed upon USP4 depletion. Quantifications were normalized to camptothecin and Control siRNA-treated GFP cells (mean ± SEM; n = 3; ^∗^p < 0.05; ^∗∗^p < 0.01; ^∗∗∗^p < 0.001; n.s., not significant). See also Figure S2.

Through ensuing studies, we established that USP4 depletion reduced HR-repair efficiencies in a cell-based assay measuring chromosomal DSB repair by gene conversion (Figure 2A). Importantly, these effects were observed despite the siRNA targeting the USP4 3′ UTR having little effect on the combined S/G2-phase cell population (Figure S2A; although siUSP4-2 reduced the S/G2 population by ∼25% compared to the siRNA control, this is unlikely to account for the ∼60% reduced HR efficiency). USP4 depletion also reduced NHEJ as assessed by a random plasmid integration assay (Figure S2B). Although USP4 involvement in NHEJ will be worth pursuing, our studies focused on its impact on HR and related events. Importantly, in line with our other studies implying that USP4 promotes DSB repair via mechanisms distinct from its spliceosomal functions in concert with SART3, HR was not significantly altered by SART3 depletion (Figure S2D; see Figure S2C for siSART3 depletion).

In accordance with USP4 functioning in HR, its depletion sensitized cells to camptothecin (Figure 2B), which yields replication-associated one-ended DSBs in S-phase that must be repaired by HR. In HR, RAD51 assemblies replace replication protein A (RPA) on resected ssDNA to form nucleoprotein filaments that mediate strand-invasion and ensuing HR events. As these RAD51 assemblies can be detected as IR-induced sub-nuclear foci (IRIF) (Polo and Jackson, 2011), we assessed whether their formation was affected by USP4. Indeed, USP4 depletion reduced the proportion of cells exhibiting RAD51 foci that co-localized with γH2AX, indicating that RAD51 loading at DSB sites was compromised (Figure S2E; BRCA1 depletion was used as control). When loaded on ssDNA, the RPA subunit RPA2 is phosphorylated on Ser-4 and Ser-8, and inhibition of all known DNA-end resection factors reduces this mark (e.g., Gravel et al., 2008, Polo et al., 2012, Sartori et al., 2007). In line with USP4 affecting resection, its depletion reduced RPA2 Ser-4/Ser-8 phosphorylation (S4S8p) at various times after IR (Figure 2C) or camptothecin exposure (Figures 2D and S2F). Importantly, γH2AX intensities after camptothecin treatment were not significantly affected when cells were treated with the siRNA targeting the USP4 3′ UTR (Figure S2F), implying that S-phase entry and progression were not markedly altered by USP4 depletion (siUSP4-2 treatments reduced S-phase cell populations by ∼25% [Figure S2A], probably partially accounting for reduced γH2AX and S4S8p RPA2 after siUSP4-2 treatment [Figure S2F]). We also found that expression of GFP-USP4 WT(L) at levels similar to those of endogenous USP4, partially rescued the RPA2 phosphorylation defect caused by depleting endogenous USP4 (Figure S2G). Furthermore, pulse-labeling cells with 5-ethynyl-2′-deoxyuridine (EdU) followed by assessment of its incorporation into DNA using EdU labeling indicated that overall levels of DNA replication in S-phase cells were not significantly altered by USP4 depletion (Figure S2H). Collectively, these data strongly suggested that USP4 promotes DNA-end resection.

To more directly address resection efficiencies in S-phase cell populations, we pulse-labeled cells with 5-bromo-2′-deoxyuridine (BrdU), treated them with camptothecin, and then probed for BrdU incorporation under native conditions where BrdU is detected in ssDNA but not dsDNA. Flow-cytometry-based quantification established that USP4 depletion (Figure 2E), but not SART3 depletion (Figure S2I), reduced native BrdU staining intensities in replicating cells without USP4 or SART3 depletion affecting protein levels of key resection factors (Figure S2J). Furthermore, the resection defect caused by depleting endogenous USP4 was largely restored by GFP-USP4 WT(L) expression (Figure 2F). Taken together, these data indicated that, in a SART3-independent manner, USP4 promotes resection, thus at least in part explaining its impact on HR.

### USP4 Regulates CtIP Recruitment to DNA Damage Sites

**Figure 3:**
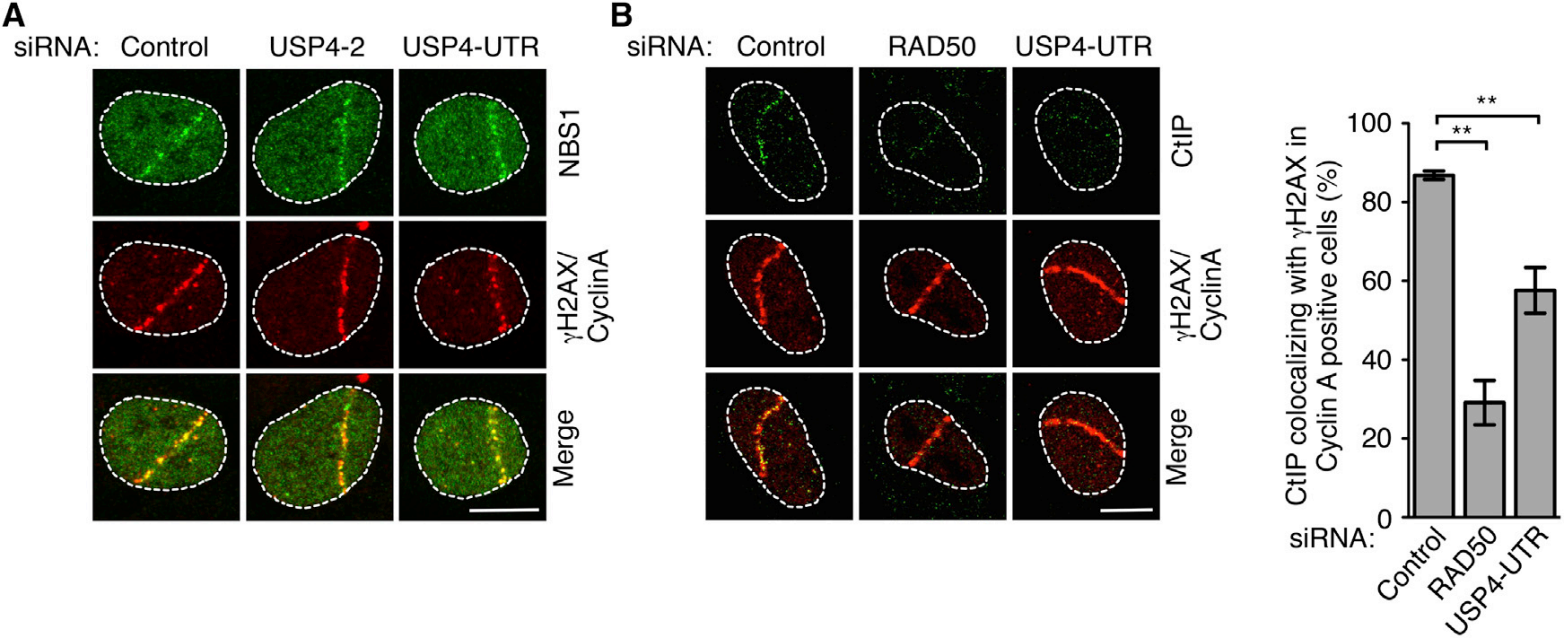
USP4 Regulates CtIP Recruitment to DNA Damage Sites (A) NBS1 recruitment to laser-line micro-irradiation induced DNA lesions in Cyclin A-positive U2OS cells that were treated with USP4-targeting siRNAs was not reduced compared to the siRNA control. Nuclei were outlined, and the scale bar indicated 10 μm. (B) CtIP recruitment to laser-line micro-irradiation induced DNA lesions in Cyclin A-positive U2OS cells was reduced after USP4 siRNA treatments. RAD50 siRNA treatment (see Figure S3A for RAD50 depletion) was the positive control (mean ± SEM; n = 3; ^∗∗^p < 0.01). Nuclei were outlined, and the scale bar indicated 10 μm. See also Figure S3.

Because the MRN complex and CtIP play key roles in DSB resection (Sartori et al., 2007), we assessed whether their recruitment to DNA damage sites was affected by USP4. We observed that USP4 depletion did not detectably affect NBS1 (Figure 3A) or MRE11 (data not shown) recruitment to DNA damage induced by laser micro-irradiation. By contrast, CtIP recruitment to DNA damage sites in γH2AX and Cyclin-A-positive cells was significantly reduced upon USP4 depletion (Figure 3B; CtIP recruitment was also impaired in RAD50-depleted cells; see Figure S3 for RAD50 depletion). These data thus implied that USP4 promotes HR by affecting the recruitment and/or association dynamics of CtIP at DNA damage sites.

### USP4 Interacts with CtIP and MRN via Its C-Terminal Insert Region

**Figure 4:**
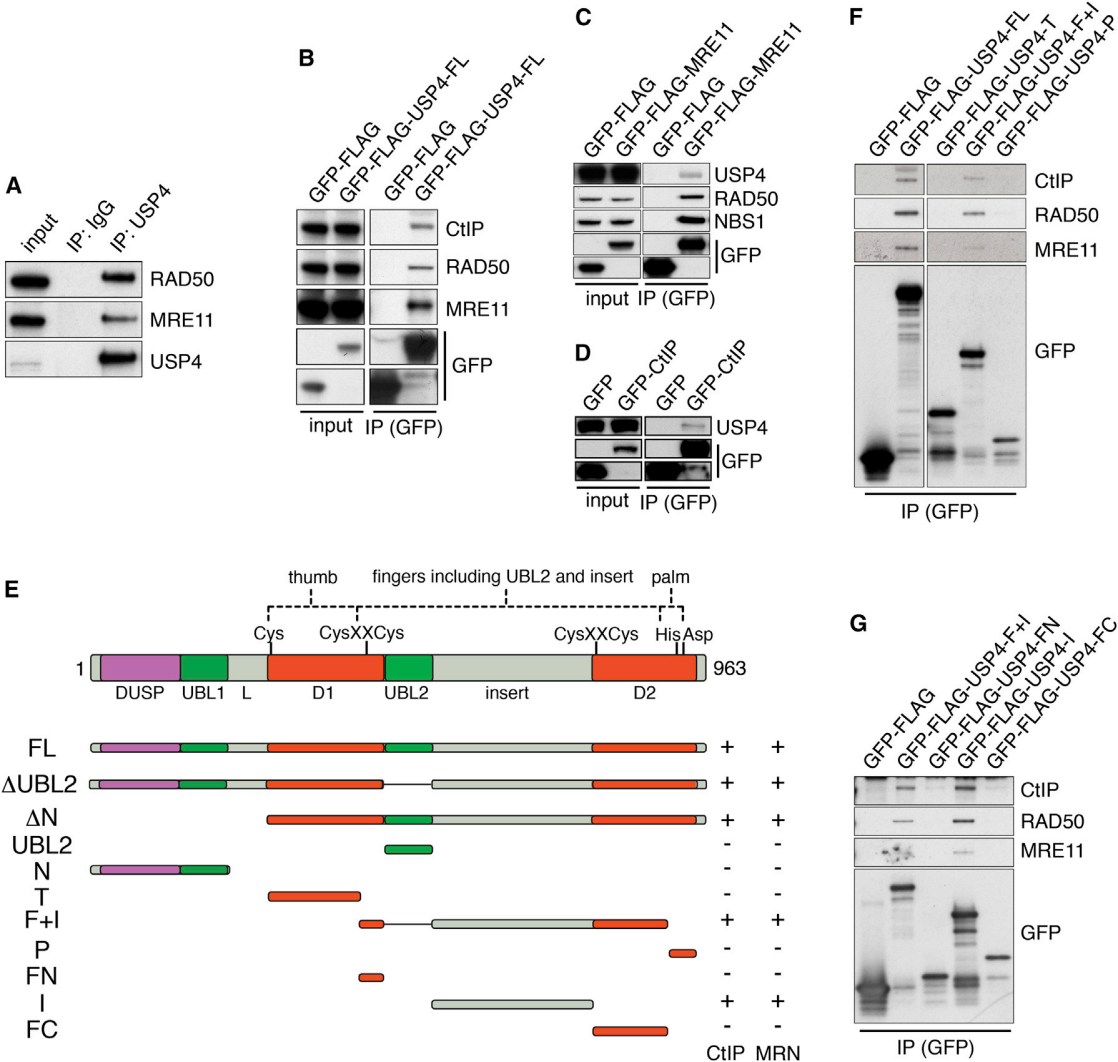
USP4 Interacts with CtIP and MRN via Its C-Terminal Insert Region (A) Endogenous USP4 immunoprecipitation (IP) from 293FT cell extracts retrieved RAD50 and MRE11. (B) Full-length (FL) GFP-FLAG-USP4 IP from U2OS cell lysates retrieved CtIP, RAD50, and MRE11. (C and D) (C) GFP-FLAG-MRE11 or (D) GFP-CtIP immunoprecipitations from 293FT cell extracts retrieved USP4. (E) Schematic view of full-length (FL) USP4 with indicated structural domains. USP4 deletion mutants and their ability to retrieve CtIP or MRN were indicated. Positions of cysteine, histidine and aspartic-acid that form the USP4 catalytic triad; the zinc-binding motif cysteine residues (CysXXCys); and the “thumb,” “fingers,” and “palm” catalytic subdomains were indicated. (F) GFP-FLAG-USP4-F+I immunoprecipitations retrieved CtIP, RAD50, and MRE11 (See Figure S4C for corresponding inputs; all samples were run on the same SDS-poly acrylamide gel). (G) GFP-FLAG-USP4-I immunoprecipitations retrieved CtIP, RAD50, and MRE11 (See Figure S4D for inputs). See also Figure S4.

In light of the above findings, we tested whether USP4 might physically interact with CtIP and/or MRN. Indeed, when we immunoprecipitated endogenous USP4 from human 293FT cell extracts ensuing western blotting analyses readily detected both RAD50 and MRE11 (Figure 4A). Despite it being well established that CtIP interacts with MRN (e.g., Sartori et al., 2007, Wang et al., 2013), we did not detect CtIP in our immunoprecipitates. In line with our speculation that this might reflect CtIP interactions being disrupted/weakened by the anti-USP4 antibody, CtIP was detected together with RAD50 and MRE11 in immunoprecipitates generated by using an anti-GFP antibody and lysates from cells transiently expressing GFP-FLAG-fused full-length (FL) USP4 (GFP-FLAG-USP4-FL; Figure 4B; GFP-FLAG only expression was used as control. As shown in Figure S4A, these interactions were not discernibly affected by DNA damage induction). Such interactions were also seen in reciprocal studies where GFP-CtIP or GFP-FLAG-MRE11 was immunoprecipitated and ensuing samples probed for endogenous USP4 (Figures 4C and 4D, respectively).

To identify the region(s) of USP4 mediating its MRN/CtIP interactions, we expressed various USP4 deletion mutants in cells (Figure 4E; Table S3), immunoprecipitated them, and then probed for CtIP, RAD50, and MRE11 binding by western blotting. This established that interactions with CtIP and MRN were not diminished by deleting the USP4 ubiquitin-like domain 2 (UBL2) region (ΔUBL2) or the N-terminal ∼30% of USP4 (ΔN). Furthermore, CtIP and MRN did not detectably interact with the UBL2 or N-terminal domain of USP4 (Figures 4E and S4B; data not shown). These results thus indicated that USP4 interactions with CtIP and the MRN complex likely occurred via the USP4 C-terminal catalytic region D1 and D2 domains and/or the C-terminal insert region (I) positioned between these domains (Figure 4E). Focusing on the USP4 catalytic domain, which structurally resembles an open right hand comprising three regions named the “thumb” (T), “fingers” (F), and “palm” (P) catalytic sub-domains (Clerici et al., 2014), we found that the USP4 “fingers” domain including the insert (F+I), but not the other regions tested, was sufficient to mediate interactions with CtIP, MRE11, and RAD50 (Figures 4E and 4F; see Figure S4C for corresponding inputs). Further analyses indicated that the USP4 insert (I), but not the fingers (F), region was sufficient for these interactions (Figures 4E and 4G; see Figure S4D for inputs), although we note that additional USP4 regions might also contribute to interactor binding.

### USP4 Counteracts Its Own Ubiquitylation

**Figure 5:**
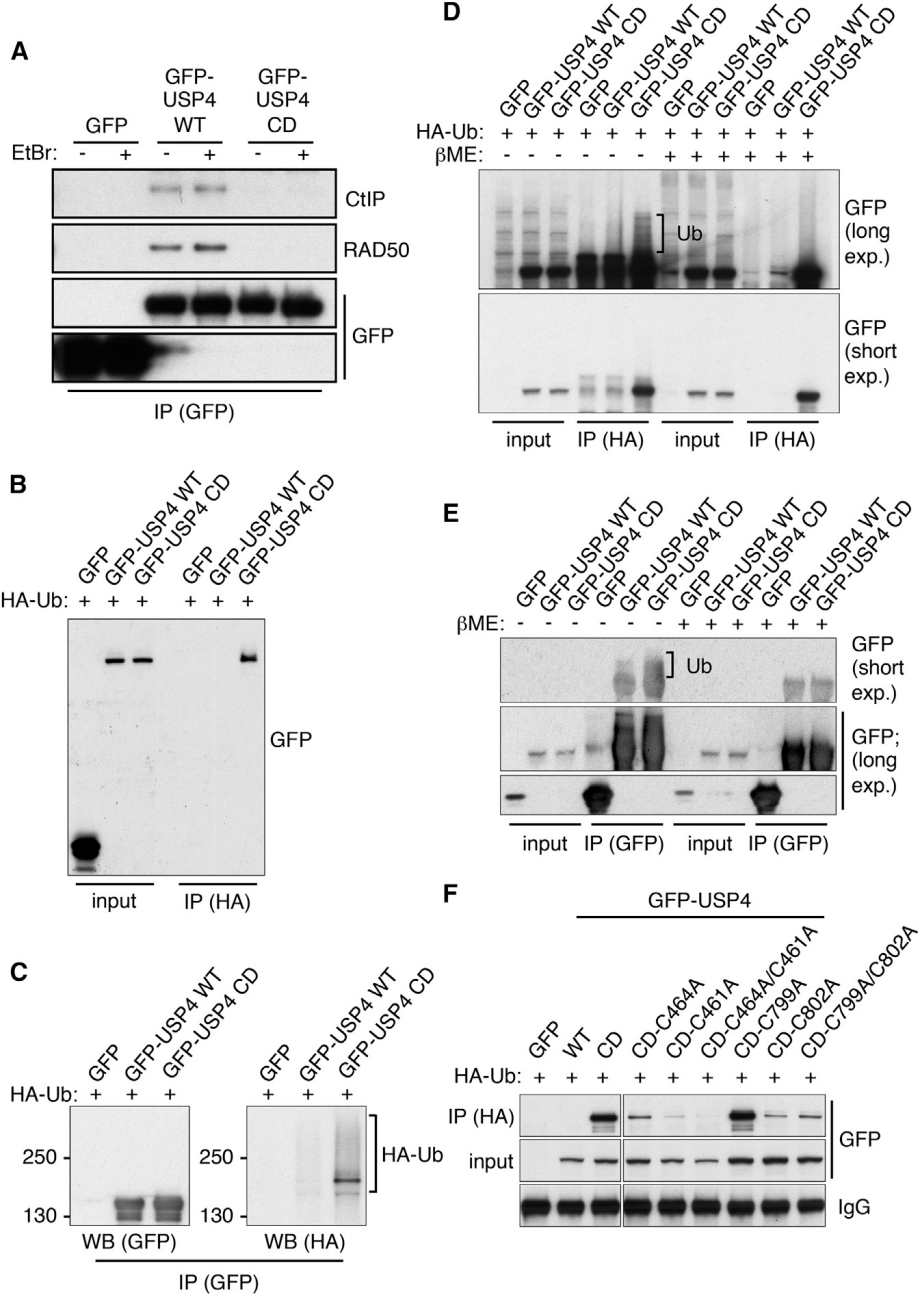
USP4 Counteracts Its Own Ubiquitylation (A) GFP-USP4 WT but not CD immunoprecipitations from U2OS cell extracts retrieved CtIP and RAD50 in presence or absence of EtBr (50 μg/ml; see Figure S5A for inputs). (B) HA-ubiquitin (HA-Ub) immunoprecipitations (in presence of 1 M NaCl) retrieved GFP-USP4 CD but no detectable GFP-USP4 WT from U2OS cell extracts. (C) GFP-immunoprecipitations from U2OS cell extracts that expressed HA-ubiquitin and GFP, GFP-USP4 WT, or CD, followed by western blot analysis with an HA antibody retrieved HA-ubiquitylated forms of GFP-USP4 CD and to a lesser extent GFP-USP4 WT (130 or 250 indicated respective protein sizes in kDa). (D) HA-Ub immunoprecipitations from U2OS cell extracts that were processed in absence of β-mercaptoethanol (βME) retrieved modified forms of GFP-USP4 CD that were not visible in presence of βME (exp., exposure; GFP cells were the control). (E) GFP immunoprecipitations from U2OS cell extracts that were processed in absence of βME, retrieved modified forms of GFP-USP4 CD, and to a lesser extent GFP-USP4 WT, which were not visible in presence of βME. (F) HA-Ub immunoprecipitations (followed by western blotting analysis) from U2OS cell extracts expressing various GFP-fused USP4 derivatives retrieved GFP-USP4 CD and CD-C799A, but less efficiently the other zinc-binding motif cysteine mutants (CD-C461A, CD-C464A, and CD-C802A; IgG, IgG heavy chain). All samples were run on the same SDS poly-acrylamide gel. See also Figure S5 and Table S4 (describes all ubiquitin sites identified by tandem mass spectrometry on USP4 WT and CD).

During the course of our studies, we observed that mutating the USP4 catalytic cysteine to alanine (C311A) to render USP4 enzymatically inactive (“catalytic-dead” [CD]), almost totally abrogated its interactions with CtIP and MRN (Figure 5A; note that binding of USP4 to CtIP and RAD50 was not abrogated by the DNA-intercalating agent ethidium bromide (EtBr), suggesting that interaction was not mediated by DNA bridging. See Figure S5A for inputs). In light of previous work indicating that USP4 can deubiquitylate itself (Wada et al., 2006), we hypothesized that USP4 catalytic inactivation could lead to its enhanced ubiquitylation, which might block its CtIP/MRN interactions. We therefore assessed ubiquitylation of GFP-USP4 WT, GFP-USP4 CD, and GFP (assessment of endogenous USP4 ubiquitylation events could more directly address the physiological nature of such modifications but was technically not feasible; data not shown) by co-expressing these with human influenza hemagglutinin-epitope-tagged ubiquitin (HA-Ub) and immunoprecipitating ubiquitylated proteins with an HA antibody in the presence of 1 M NaCl. Western blotting of ensuing samples with a GFP antibody indicated that HA-ubiquitin retrieved USP4 CD but not USP4 WT or GFP alone at appreciable levels (Figure 5B). Although these results might have been explained by USP4 CD displaying enhanced non-covalent binding to ubiquitin than wild-type USP4, when we carried out binding studies with recombinant ubiquitin, this was not the case (Figure S5B). To verify that USP4 was indeed ubiquitylated, we prepared GFP-immunoprecipitates from lysates of cells co-expressing HA-ubiquitin together with GFP-USP4 WT or CD. Probing ensuing western blots with an HA antibody detected smears of slower migrating products, thus identifying these as ubiquitylated USP4 derivatives (Figure 5C). Furthermore, in line with our other findings and previously reported USP4 ubiquitylation events (Wada et al., 2006), these ubiquitylated species were more prominent with GFP-USP4 CD than with GFP-USP4 WT (Figure 5C). Together with the stringency of the immunoprecipitation conditions we used, these data indicated that USP4 is ubiquitylated and that catalytically dead USP4 contained greater levels of covalently bound ubiquitin than the wild-type protein.

To assess USP4 ubiquitylation further, we carried out tandem mass spectrometry studies on GFP-USP4 CD or WT, purified from cell lysates via GFP-immunoprecipitations, followed by post-translational modification analysis to identify GlyGly modifications on amino acid residues, representing remains of ubiquitin or ubiquitin-like modifications after trypsin digestion. Thus, we detected such modifications on a considerable number of lysine residues and also on serine and threonine residues (see Table S4). Notably, our analysis also suggested ubiquitylations on non-conventional cysteine residues of both USP4 CD and WT. In particular, we noted evidence for modifications on Cys-461 or Cys-464 and Cys-799 or Cys-802 (Figures S5C and S5D; data not shown; as each pair of cysteine residues was on the same trypic peptide, it was not possible to differentiate between them), which form a flexible zinc-binding region that stabilizes the catalytic domain of USP4 (Clerici et al., 2014). Further analysis and quantification of these ubiquitylations by mass spectrometry was not possible however, due to technical issues relating to the labile nature of ubiquitin-thioester linkages (see below) and confounding modifications of these and other USP4 cysteine residues by chemical reagents used in sample preparations (see Supplemental Information for further details).

Previous reports have proposed the existence of ubiquitin adducts in which the ubiquitin C terminus is covalently attached to target protein serine or threonine residues by ester linkages or cysteine residues by thioester linkages (e.g., Cadwell and Coscoy, 2005). While cysteine ubiquitylation has so far been largely unexplored, its prevalence may have been underestimated because the associated thioester linkage is readily disrupted by reducing agents (Huang et al., 2009) such as β-mercaptoethanol that are often used in cell extract generation and analysis. In light of this and our other data, we tested whether we could detect USP4 ubiquitylations that were sensitive to β-mercaptoethanol treatment. Thus, through HA-ubiquitin immunoprecipitations followed by western blotting in the absence or presence of β-mercaptoethanol, we identified modified forms of GFP-USP4 CD that migrated more slowly on SDS polyacrylamide gels than GFP-USP4 WT and which were lost upon β-mercaptoethanol treatment (Figure 5D). To confirm this finding, we carried out GFP immunoprecipitations from extracts containing GFP-tagged USP4 CD or USP4 WT and processed these the in absence or presence of β-mercaptoethanol. Western blot analysis with an antibody recognizing GFP revealed that USP4 CD and to a lesser extent USP4 WT were modified and that these modifications were not observed when samples had been treated with β-mercaptoethanol (Figure 5E; note that USP4 WT and CD were present in similar amounts and that it is unlikely that the observed modifications reflected differential oxidation events between wild-type and catalytically dead USP4). These results indicated that at least a fraction of ubiquitylated USP4 CD was sensitive to reducing conditions, thus supporting the conclusion that the protein is subject to cysteine ubiquitylation.

Structural data (Clerici et al., 2014) indicate that Cys-799, -802, -461, and -464 form a flexible zinc-binding region that stabilizes the USP4 catalytic domain and is exposed outward from the protein core, potentially making the region accessible for modification. While exploring the possible functional impact of these cysteine residues, we found that USP4 CD derivatives containing cysteine to alanine mutations on Cys-461 (C461A), Cys-464 (C464A), and Cys-802 (C802A) but not Cys-799 (C799A) upon co-expression in cells with HA-ubiquitin, were less readily retrieved by HA-ubiquitin immunoprecipitations from cell extracts than the GFP-USP4 CD protein itself (Figure 5F). While these findings provided support for the zinc-binding motif cysteine residues being ubiquitylated, it is also possible that mutating these sites altered USP4 structurally in a manner that reduced its overall ubiquitylation levels, perhaps by making it a less effective target for relevant E3 ubiquitin ligases. Nevertheless, as shown in Figure S5E, we found that although binding of an HA-ubiquitin activity probe was lower for USP4 WT-C464A than for USP4 WT, the Cys-464 mutation still maintained catalytic activity, implying that the USP4 structure was still at least in part intact. Together, these findings supported a model in which USP4 ubiquitylations, including those on zinc-binding cysteine residues, are subject to turnover by USP4 catalytic activity.

### Ubiquitylation Counteracts USP4 Interactions and Function

**Figure 6:**
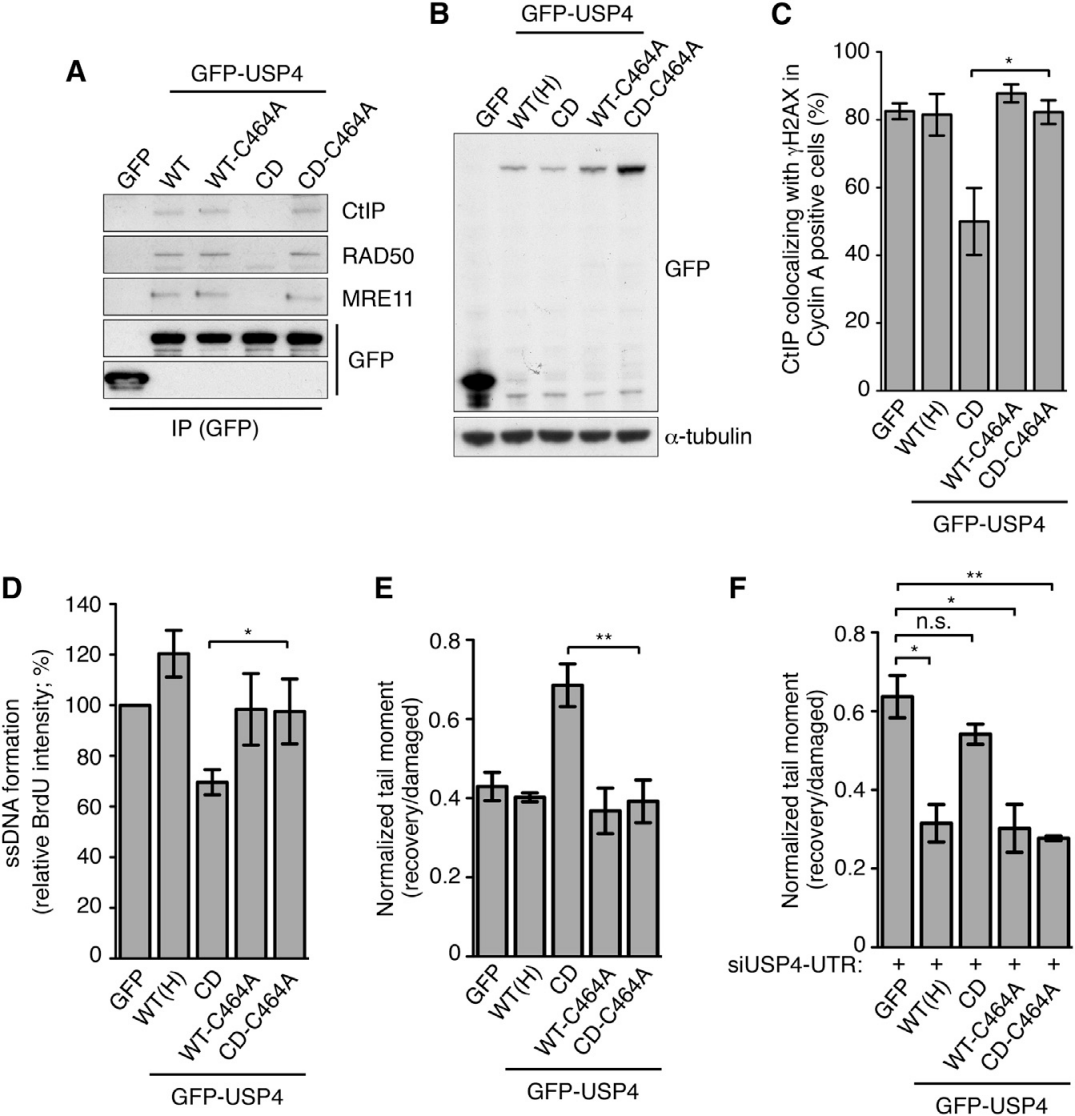
Ubiquitylation Counteracts USP4 Interactions and Function (A) GFP-USP4 WT, WT-C464A, and CD-C464A but not CD immunoprecipitations retrieved CtIP, RAD50, and MRE11 (see Figure S5A for inputs). (B) Protein levels of U2OS cells expressing GFP-USP4 WT-C464A or GFP-USP4 CD-C464A. GFP, GFP-USP4 WT (H) and CD cell lines were described previously (Figure S1C). (C–E) Mutating USP4 Cys-464 to Ala restored (C) CtIP recruitment defects (mean ± SEM; n = 3), (D) DNA-end resection defects (mean ± SEM; n = 5), and (E) DSB repair (neutral comet assays) defects (mean ± SEM; n = 3), observed with GFP-USP4 CD-expressing U2OS cells. (F) Mutating Cys-464 to an alanine in USP4-UTR siRNA-treated U2OS cells restored the DSB repair defects observed in GFP-USP4 CD expressing cells upon endogenous USP4 depletion (mean ± SEM; n = 3; ^∗^p < 0.05; ^∗∗^p < 0.01). See also Figure S6.

Our data highlighted how disrupting USP4 catalytic activity abrogated its interactions with CtIP and MRN and also led to enhanced USP4 ubiquitylation, suggesting that these phenomena might be mechanistically linked. To address this possibility, we focused on USP4 Cys-464, whose mutation strongly reduced retrieval of USP4 CD by HA-ubiquitin immunoprecipitations. Thus, we carried out immunoprecipitation-western blot analyses to see whether introducing the Cys-464 to Ala (C464A) mutation into USP4 CD (in which the USP4 catalytic cysteine residue C311 was mutated to alanine) might restore interactions with CtIP and MRN. Indeed, while having little or no effect on CtIP/MRN interactions with GFP-USP4 WT, the C464A mutation markedly stimulated interactions between GFP-USP4 CD and CtIP/MRN (Figure 6A; see Figure S6 for corresponding inputs). To explore whether this compensatory effect extended to USP4 functions in the DDR, we generated U2OS cell lines stably expressing GFP-USP4 WT-C464A or GFP-USP4 CD-C464A (Figure 6B). Analyses of these and the previously described GFP-USP4 WT(H) and CD cell lines established that mutating the USP4 catalytic cysteine to alanine resulted in CtIP recruitment and resection defects that were largely alleviated by the C464A mutation (Figures 6C and 6D, respectively). These results thus supported a model in which USP4 functions primarily through mediating interactions with CtIP and the MRN complex rather than by targeting these or other factors for deubiquitylation.

As we had previously found that expression of USP4 CD functioned in a dominant-negative manner (Figure 1G and Figure S1F), we assessed whether this was abrogated by the C464A mutation. Indeed, neutral comet assays indicated that GFP-USP4 CD but not GFP-USP4 CD-C464A expression caused DSB repair defects in cells after phleomycin treatment (Figure 6E). Importantly, GFP-USP4 CD but not GFP-USP4 CD-C464A expression also caused DSB repair defects when endogenous USP4 was depleted from cells by siRNA treatment (Figure 6F), establishing that GFP-USP4 CD-C464A functions directly to promote DSB repair without endogenous USP4 contributing to the phenotype. Together, these findings provided support for USP4 interactions with CtIP/MRN being critical for its DDR functions and for a model wherein USP4 auto-deubiquitylation promotes these interactions and thereby USP4 functions in DSB repair.

### Auto-Regulated Ubiquitylation of Other USP-Family DUBs

**Figure 7:**
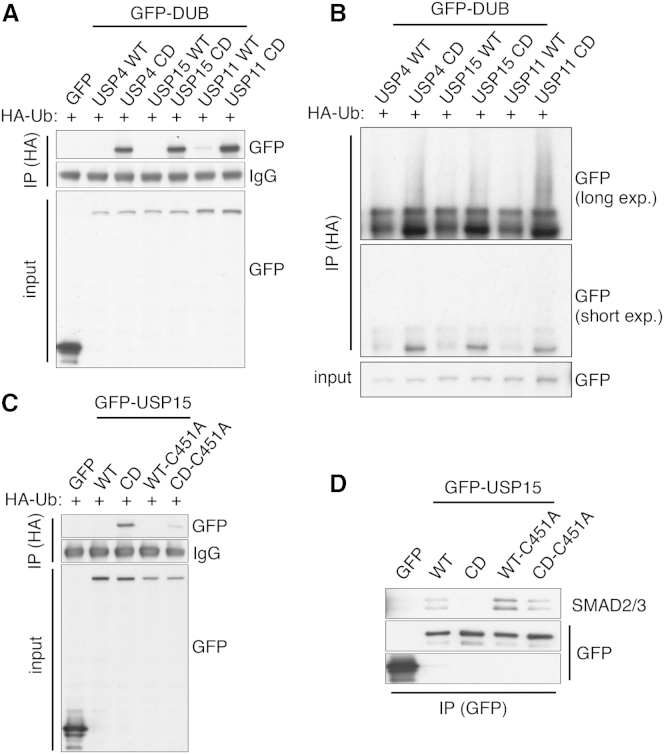
Auto-Regulated Ubiquitylation of Other USP-Family DUBs (A) HA-ubiquitin (HA-Ub) immunoprecipitations (in presence of βME) retrieved USP4 CD, USP15 CD, and USP11 CD (wild-type USP4, USP15, and USP11 were not or weakly detected under these conditions). (B) HA-Ub immunoprecipitations from U2OS cell extracts that were processed without βME retrieved modified forms of USP4 CD, USP15 CD, and USP11 CD (exp., exposure). (C) HA-Ub immunoprecipitations from U2OS cell extracts retrieved USP15 CD-C451A less efficiently than USP15 CD. (D) USP15 WT, WT-C451A, and CD-C451A but not CD immunoprecipitations from U2OS cell extracts (see Figure S7B for inputs) efficiently retrieved SMAD2/3. See also Figure S7.

Based on the above findings, we hypothesized that, like USP4, other USP-family DUBs might be subject to ubiquitylation to regulate protein interactions in a manner counteracted by their catalytic activities. Focusing on USP15 and USP11, the two DUBs most related to USP4, we rendered them catalytically inactive by mutating their catalytic cysteine residue to alanine (USP15 C298A and USP11 C318A; see Figure S7A for sequence alignments). Strikingly, co-expression of these or wild-type versions with HA-ubiquitin followed by immunoprecipitations with an HA antibody and western blot analysis under reducing conditions revealed that USP15 CD and USP11 CD were retrieved more strongly than their corresponding wild-type proteins (Figure 7A). Furthermore, as for USP4 CD, when samples were analyzed under non-reducing conditions, USP15 CD and USP11 CD exhibited additional slower-migrating species (Figure 7B). These findings thus suggested that, as for USP4, the catalytic activities of USP15 and USP11 counteract their respective ubiquitin modifications. To investigate USP15 further, we focused on USP15 Cys-451, which forms part of the USP15 zinc-binding motif and aligns with USP4 Cys-464, whose mutation to alanine reduced the ability of USP4 CD to be retrieved by HA-ubiquitin under stringent immunoprecipitation conditions (see Figure 5D). Notably, transient co-expression studies employing HA-ubiquitin and various USP15 derivatives followed by HA immunoprecipitation-western blotting indicated that introducing the C451A mutation reduced the amount of GFP-USP15 CD recovered with HA-ubiquitin (Figure 7C). In light of our USP4 findings, we tested whether catalytically dead USP15 was still able to interact with one of its established substrates, SMAD2/3 (Inui et al., 2011), and if this interaction was influenced by C451A mutagenesis. Thus, through immunoprecipitation-western blot analyses, we found that, unlike USP15 WT, USP15 CD was impaired in its ability to interact with SMAD2/3 and that the USP15 CD-C451A mutant restored this interaction (Figure 7D; see Figure S7B for inputs). These findings thereby supported a model in which, as for USP4, USP15 catalytic activity counteracts its own ubiquitylation to promote substrate interactions.

## Discussion

We have established that USP4 promotes DSB repair by HR and cellular resistance to IR and the topoisomerase I inhibitor camptothecin. Mechanistically, we found that USP4 does so at least in part by promoting DSB resection in a manner that appears to be independent of its established spliceosomal functions. Accordingly, we established that USP4-depleted cells display defects in DNA-damage-induced RPA2 phosphorylation and in RAD51 accumulation at DNA damage sites. Moreover, we found that USP4 depletion markedly impaired DNA-damage accumulation of the DNA-end resection factor CtIP. USP4 thus joins a growing number of proteins known to affect CtIP activity, highlighting the crucial importance of appropriately controlling and regulating the initiation of resection.

By assessing the properties of USP4 derivatives, we discovered that it directly or indirectly interacts with CtIP and MRN and that the USP4 insert region, which resides between the USP4 D1 and D2 catalytic subdomains, was sufficient to mediate such interactions. Moreover, we observed that inactivating USP4 catalytic function almost totally abrogated its CtIP/MRN interactions. Through exploring the mechanism of this effect, we found that catalytically inactive USP4 was retrieved more effectively by HA-tagged ubiquitin than the wild-type USP4 protein, leading us to investigate whether its CtIP/MRN interactions might be affected by USP4 auto-deubiquitylation. Indeed, by mass-spectrometry, we identified various USP4 ubiquitylations that were enhanced upon USP4 catalytic inactivation, including those on cysteine residues within an evolutionarily conserved USP4 zinc-binding motif. Consistent with the chemical nature of these ubiquitin thioester linkages, our ensuing studies highlighted their removal by β-mercaptoethanol. Moreover, mutating such cysteine residues to alanine prevented USP4 retrieval by HA-ubiquitin, restored the ability of catalytically dead USP4 to interact with CtIP/MRN, and also restored the ability of catalytically inactive USP4 to promote DSB repair, even in the absence of endogenous USP4. Taken together, our observations support a model in which USP4 is subject to ubiquitylation in a manner that interferes with CtIP and MRN binding, thus impairing resection and abrogating HR. Moreover, our results indicate that the key DDR role for USP4 catalytic function is to counteract modifications on itself, thereby promoting CtIP/MRN binding, resection, and HR. Additional biochemical analyses will be needed to address precisely how USP4 ubiquitylations inhibit its interactions with other proteins. One possibility is that ubiquitylation competes with ubiquitin that is retained by the USP4 ubiquitin-binding pocket and switching-loop motif, following substrate hydrolysis (Clerici et al., 2014, Sahtoe and Sixma, 2015). It remains to be established which ubiquitin ligase(s) mediate(s) USP4 ubiquitylation and whether the auto-regulatory paradigm we have established is constitutive or is affected by factors such as cell-cycle status, chromatin structure, or DDR signaling.

In contrast to the extensive literature on lysine ubiquitylation, few reports have described ubiquitylation of cysteine residues (e.g., Cadwell and Coscoy, 2005) other than on E1-activating enzymes, E2-conjugating enzymes, and HECT-domain E3 ubiquitin ligases (Komander, 2009a). Our mapping of USP4 cysteine ubiquitylations and our observation that such ubiquitylations are labile under reducing conditions, highlight how cysteine ubiquitylation and deubiquitylation might occur more generally, at least within the USP-DUB ubiquitin protease family, many of which contain zinc-binding cysteine motifs (Ye et al., 2009). Furthermore, we found that, as for USP4, USP15 and USP11 catalytic inactivation led to the accumulation of modified forms that were abrogated by reducing agents and that mutating Cys-451 of USP15, which aligns with USP4 Cys-464, reduced USP15 retrieval by HA-ubiquitin. Moreover, we established that, analogously to USP4, catalytically inactive USP15 was impaired in binding to its protein target, SMAD2/3, and that binding was restored by introducing the Cys-451 mutation. In light of the phylogenetic connections between USP4, USP15, and USP11, and because both USP4 (this study) and USP11 function in DNA repair (Schoenfeld et al., 2004, Wiltshire et al., 2010), it will be of interest to explore possible DDR roles for USP15. In this regard, we note that USP15 has been identified as a target for ATM-mediated phosphorylation (Mu et al., 2007) and mediates resistance to IR (Nishi et al., 2014) and that like USP4, USP11 and USP15 are recruited to sites of laser-induced DNA damage (Nishi et al., 2014). Finally, if small-molecule inhibitors of USP4, USP15, and/or USP11 are developed, it will be interesting to pursue their potential in cancer therapy.

## Experimental Procedures

For detailed descriptions of these and additional procedures, see Supplemental Experimental Procedures.

### Cells, Cell Lines and Growth Conditions

U2OS cells were cultured under conventional growth conditions. All stable cells lines exogenously expressing GFP or FLAG-fused USP4 and mutant derivatives and 293FT cells were cultured in presence of 0.5 mg/ml geneticin (Life technologies). DR-GFP expressing U2OS cells were cultured in presence of 1 μg/ml puromycin.

### Antibodies, SDS-PAGE, and Western Blot Analysis

See Table S2 for antibodies used in this study. SDS-PAGE and western blot analyses were performed as described previously (Nishi et al., 2014).

### siRNAs, Plasmids, and Transfections

See Table S1s and S3 for respective siRNAs and plasmids described in this study. Plasmids were transfected using TransIT-LT1 (Mirus Bio) transfection reagent according to the manufacturer’s instructions, and siRNA transfections (30 nM/transfection) were carried out using Hiperfect (QIAGEN) according to the manufacturer’s instructions.

### Neutral Comet and Clonogenic Cell Survival Assays

Neutral comet and clonogenic cell survival assays were performed as previously described (Nishi et al., 2014).

### Immunoprecipitations

Immunoprecipitation experiments from U2OS or 293FT cells were performed as previously described (Blackford et al., 2015).

### Live Cell Laser-Line Micro-Irradiation

GFP-USP4 WT(H)-expressing cells were BrdU sensitized and then subjected to 400 μW localized laser micro-irradiation with a 405 nm UVA laser beam (Limoli et al., 1993). Pictures were taken before and 30 min after irradiation.

### DR-GFP HR Reporter Assays

HR reporter assays were performed as previously described (Nishi et al., 2014).

### DNA-End Resection (BrdU) Assay

BrdU pulse-labeled U2OS cells were treated with 1 μM camptothecin, processed, and treated with BrdU and γH2AX primary and secondary antibodies (Table S2). Cells were analyzed by flow cytometry with γH2AX detection as a control for DNA damage.

### Cell Cycle Analysis and Random Plasmid Integration Assays

Cell cycle analyses and random plasmid integration assays to measure NHEJ efficiencies were performed as described previously (Nishi et al., 2014).

### RAD51 IRIF

Cells were treated with IR (5 Gy), allowed to recover for 8 hr, and were fixed and treated with RAD51 and γH2AX primary and secondary antibodies. γH2AX-positive cells with more than three RAD51 foci were scored.

### Click-it EDU Labeling

U2OS cells were EdU pulse-labeled and fixed. EdU labeling reactions were performed using Click-iT according to the manufacturer’s instructions (Thermo Fisher Scientific). EdU intensities of S-phase cell populations were measured and quantified.

### NBS1 or CtIP Recruitment to Laser-Line Micro-Irradiation Induced DNA Lesions

U2OS cells were treated with BrdU (10 μM) for 24 hr, subjected to 250 μW localized laser micro-irradiation with a 405 nm UV-A laser beam, and 2 hr after irradiation were fixed and processed. Cyclin A, γH2AX, and NBS1- or CtIP-positive cells were quantified.

### Mass Spectrometry and Data Analysis

Samples were analyzed using nano liquid chromatography-tandem mass spectrometry (nano-LC-MS/MS) in HCD mode as described previously (Fischer and Kessler, 2015). Raw MS data were processed and analyzed by PEAKS Version 7 (Bioinformatics Solutions) using HCD fragmentation spectra. MS/MS spectra were searched against the Swissprot (21,039 human sequence entries) database allowing for variable post-translational modifications to be applied to the de novo identified peptides. See Table S4 for identified ubiquitylations on USP4 WT and CD upon immunoprecipitation with a GFP antibody from 293FT lysates. Amino acids in bold indicate previously described USP4 ubiquitylations.

### Ubiquitylation Assays

U2OS cells were co-transfected with HA-ubiquitin and GFP-tagged expression constructs. Lysates were immunoprecipitated 48 hr later with an HA antibody, and HA-retrieved proteins were subjected to western blot analysis.

### Biotin-Ubiquitin Binding Assay

Streptavidin M-280 Dynabeads (Life Technologies) were soaked in an excess of Biotin-fused human recombinant ubiquitin (R&D Systems) for 1 hr at 4°C. Then, cell lysates were incubated for 16 hr at 4°C in presence of 10 μl ubiquitin-coupled streptavidin Dynabeads, after which those were washed, processed, and subjected to western blot analysis as described in the Supplemental Experimental Procedures.

### Active Probe Binding Assays

Cells transfected with GFP, GFP-fused USP4 WT, CD, or WT-C464A were processed; incubated with HA-tagged ubiquitin vinyl sulfone (HA-Ub-VS) according to the manufacturer’s (Enzo Life Sciences) instructions; and subjected to western blot analysis.

### Statistics and Quantitative Analysis

For experiments reproduced at least three times in this study, the standard two-tailed Student’s t test for statistical significance was used. For quantitative analysis, the SEM was used. All experiments were reproduced at least twice.

## Author Contributions

P.W. designed experiments through discussions with R.N., R.K., A.N.B., J.T., B.M.K., and S.P.J.; R.N. cloned HA-ubiquitin; J.T. carried out the RAD51 IRIF experiments; P.W. and A.N.B. prepared samples for MS analyses that were carried out by R.K. and B.M.K.; P.W. carried out all further studies; P.W. and S.P.J., with input from R.N., wrote the paper; all other authors commented and suggested revisions for the paper.

## Conflicts of Interest

S.P.J. is a founder and part-time chief scientific officer (CSO) of MISSION Therapeutics Ltd., which is developing DUB inhibitors for therapeutic applications. B.M.K. is associated with Cancer Research Technologies and Forma Therapeutics. The other authors declare no competing financial interests.

## Supplemental Information

Document S1. Figures S1–S7, Tables S1–S4, and Supplemental Experimental Procedures

Document S2. Article plus Supplemental Information
