# USP4 Auto-Deubiquitylation Promotes Homologous Recombination

**DOI:** 10.1016/j.molcel.2024.12.024

**Published:** 2025-02-06

**Authors:** Paul Wijnhoven, Rebecca Konietzny, Andrew N. Blackford, Jonathan Travers, Benedikt M. Kessler, Ryotaro Nishi, Stephen P. Jackson

## Main text

(Molecular Cell *60*, 362–373; November 5, 2015)

The authors have determined after inspection of all the original data that in the originally published version of this article, an error occurred during the generation of Figure 4B. During figure preparation for this article, the authors inadvertently duplicated the CtIP input loading control bands, inserting the same bands as RAD50 input. The authors regret this error and apologize for any confusion that it has caused.

**Figure 4B:**
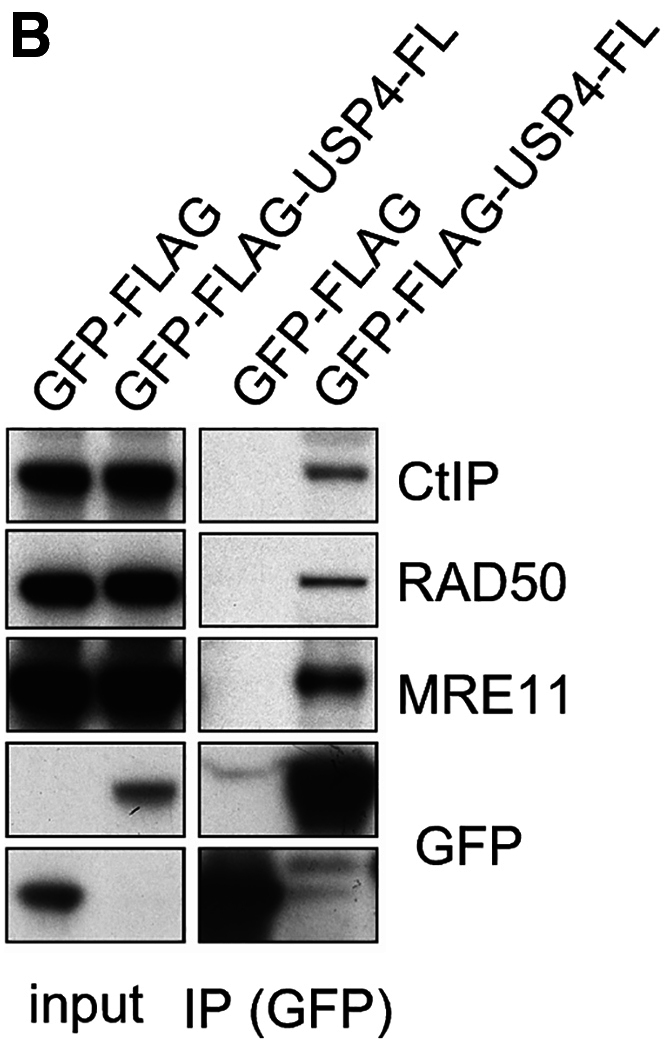
USP4 Interacts with CtIP and MRN via Its C-Terminal Insert Region

