# Supplementary material for: USP4 Auto-Deubiquitylation Promotes Homologous Recombination
Source: Mol Cell. 2015 Nov 5;60(3):362–73. doi: 10.1016/j.molcel.2015.09.019 (PMC4643306; doi:10.1016/j.molcel.2015.09.019)
Supplement: Document S1. Figures S1–S7, Tables S1–S4, and Supplemental Experimental Procedures [file mmc1.pdf]

Molecular Cell, Volume *60*

Supplemental Information

USP4 Auto-Deubiquitylation

Promotes Homologous Recombination

Paul Wijnhoven, Rebecca Konietzny, Andrew N. Blackford, Jonathan Travers, Benedikt M. Kessler, Ryotaro Nishi, and Stephen P. Jackson

Figure S1 (Related to Figure 1)

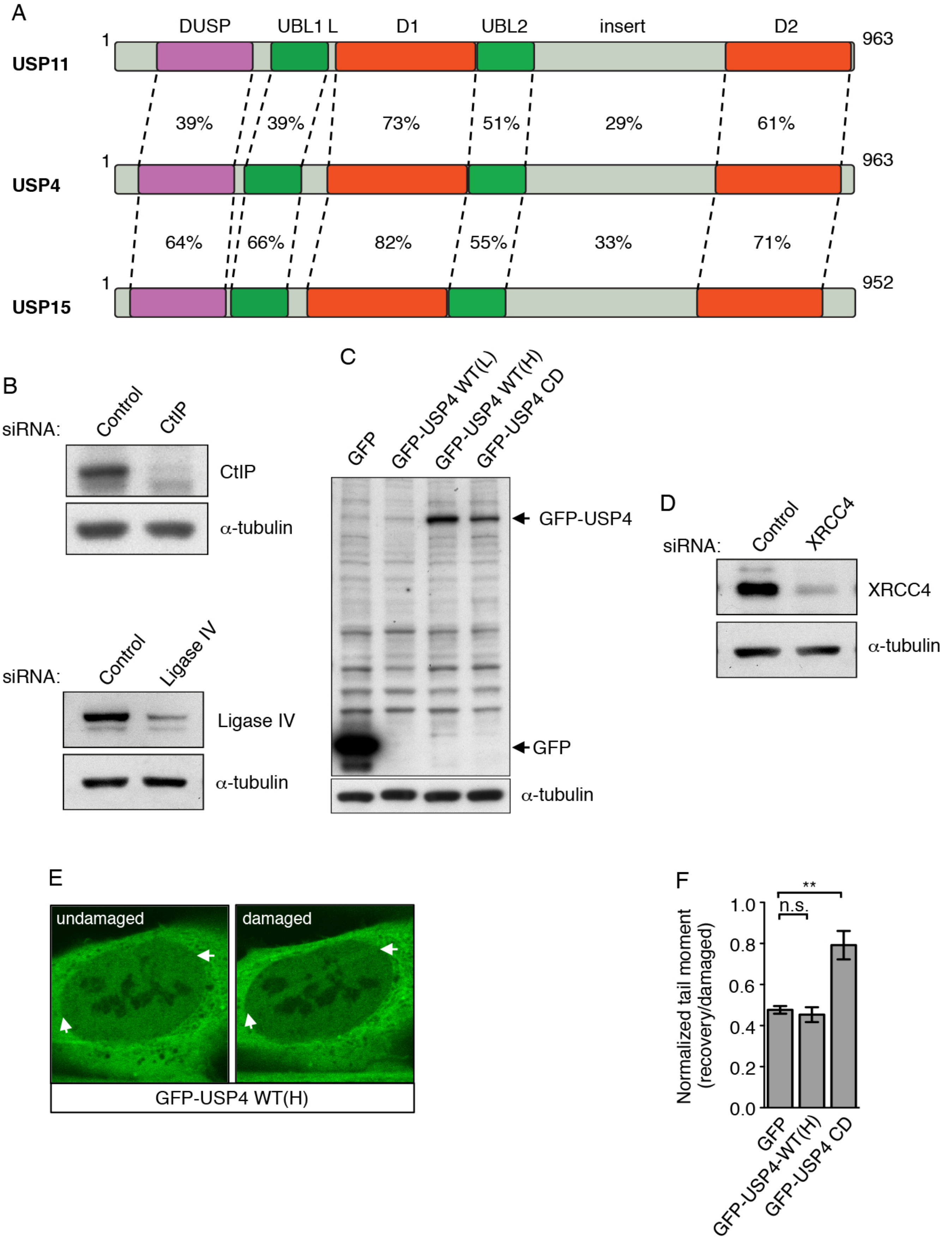

Figure S1 (Related to Figure 1)

(A) Graphic representation of USP4 and its paralogs USP15 and USP11. Domain structures and corresponding degrees of identity (%) to USP4 are indicated. DUSP = domain in USP, UBL1 = ubiquitin-like 1, L = linker region, D1 = catalytic subdomain 1, UBL2 = ubiquitin-like 2, insert = C-terminal insert, D2 = catalytic subdomain 2. (B) Treatments with CtIP or DNA Ligase IV siRNAs depleted CtIP or DNA Ligase IV from U2OS cells, respectively. Luciferase (Control) siRNA treatment was the control. (C) GFP, GFP-USP4 WT(L; where L = low expressing), GFP-USP4 WT(H; where H = high expressing) or GFP-USP4 CD protein levels from established monoclonal U2OS cell lines. (D) Treatment with XRCC4 siRNAs depleted XRCC4 in U2OS cells. Luciferase (Control) siRNA treatment was the control. (E) GFP-USP4 WT(H) stably expressed in BrdU treated (10 μM for 24 hours) U2OS cells accumulated to laser-line micro-irradiation induced DNA damage sites. (F) Exogenously expressed GFP-USP4 WT(H), but not CD rescued DSB repair defects after phleomycin treatment (neutral comet assays; mean ± s.e.m., n=3, **P < 0.01, n.s. = not significant).

Figure S2 (Related to Figure 2)

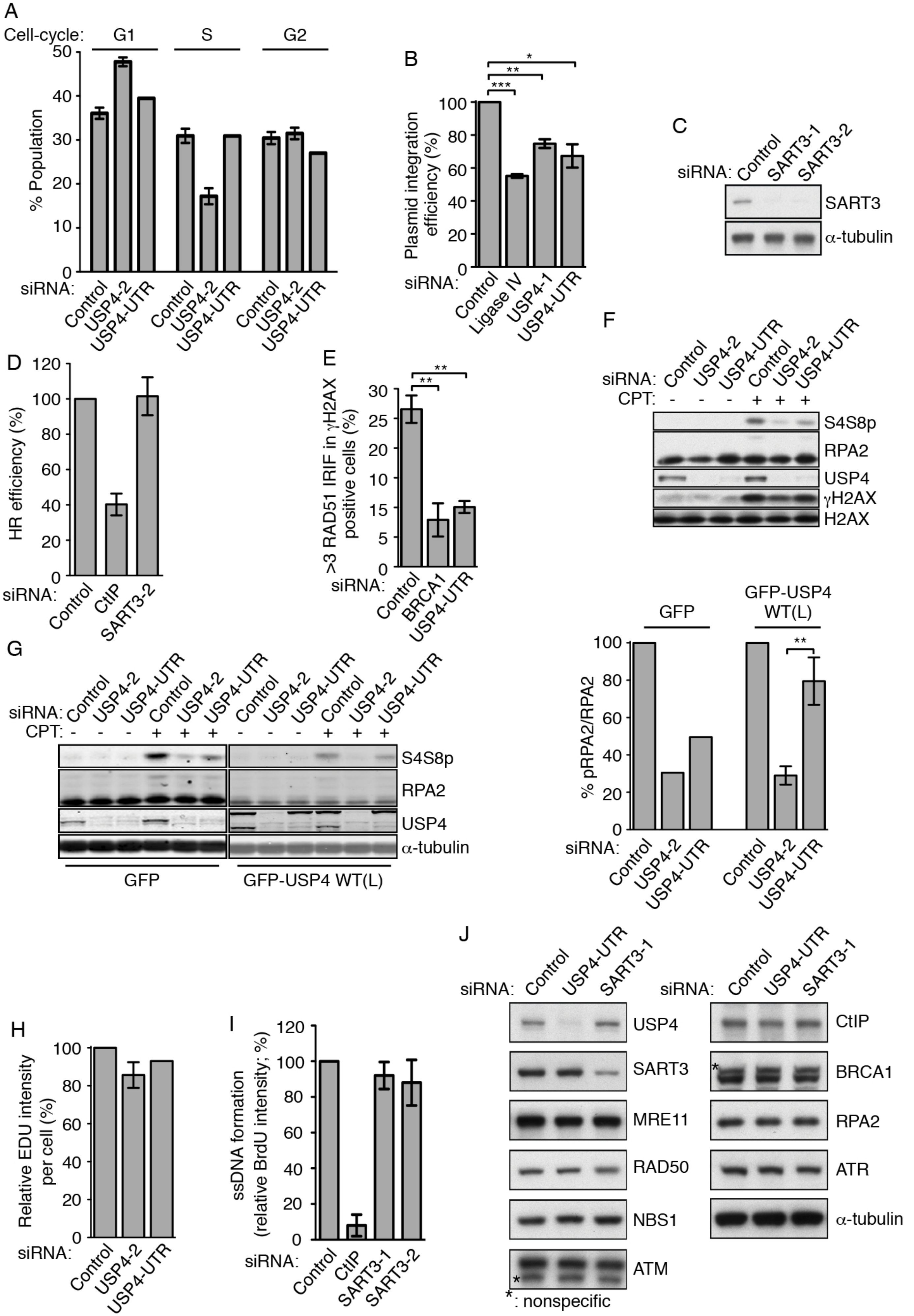

Figure S2 (Related to Figure 2)

(A) Cell cycle profiles of U2OS cells treated with luciferase (Control; n=3), USP4 [USP4-2; n=3 (mean ± s.e.m.) or USP4-UTR; n=2] siRNAs and pulse-labelled with BrdU (10 μM for 30 minutes). (B) USP4 depletion caused NHEJ defects: Random plasmid integration assays with luciferase (Control), Ligase IV or USP4 (USP4-1 or USP4-UTR) siRNA treated U2OS cells. Samples were normalized to Control siRNA treatments and set to 100% (mean ± s.e.m., n=3). (C) Treatment with SART3 siRNAs depleted SART3 from U2OS cells. Luciferase (Control) siRNA treatment was the control. (D) SART3 depletion did not cause HR defects [direct-repeat (DR)-GFP reporter assays]. Quantifications were normalized to luciferase (Control) siRNA treatments, and set to 100%. siCtIP was the positive control (mean ± s.e.m., n=4). (E) siUSP4 treatment reduced RAD51 loading after IR exposure. RAD51 ionizing irradiation-induced foci (IRIF) after luciferase (Control), BRCA1 or USP4 (USP4-UTR) siRNA treatment of U2OS cells. Cells were subsequently exposed to IR (5 Gy) and recovered for eight hours under normal growth conditions. Cells exhibiting more than three RAD51 foci in γH2AX positive cells were scored positive (mean ± s.e.m., n=3). (F) USP4 depletion reduced RPA2 Ser-4/Ser-8 phosphorylation (S4S8p) after camptothecin treatment. Luciferase (Control) or USP4 (USP4-2 or USP4-UTR) siRNA treated U2OS cells were exposed to 1 μM camptothecin for one hour. Samples were taken before and after camptothecin treatment and analyzed by western blotting with the indicated antibodies (See Figure 2D for RPA S4S8p quantifications). (G) Complementation of U2OS cells with GFP-USP4 WT(L) restored the RPA2 S4S8 phosphorylation defects observed upon siUSP4 treatment of GFP expressing cells. Representative western blots and RPA2 S4S8 phosphorylation quantifications of U2OS cells stably expressing GFP or GFP-USP4 WT(L), treated with luciferase (Control) or USP4 (USP4-2 or USP4-UTR) siRNAs and camptothecin under conditions described above [western blots describing GFP or GFP-USP4 WT(L) results were independently produced]. Per cell line, camptothecin treated GFP or GFP-USP4 WT(L) cells were set to 100% (mean ± s.e.m., GFP; n=2, GFP-USP4 WT(L); n=4). (H) USP4 depletion did not notably affect EdU incorporation during replication. U2OS cells were treated with the indicted siRNAs and pulse labelled with EdU (10 μM for 15 minutes). EdU signal intensities of S-phase cells were measured after EdU labelling (Larrieu et al., 2014) was performed and samples were normalized to Luciferase (Control) siRNA treatments, which was set to 100%. For Control and USP4-2 treatments the mean values were calculated from biological triplicates (n=3, mean ± s.e.m.) and duplicates (n=2) for USP4-UTR treatments. (I) SART3 siRNA treatment, followed by camptothecin (1 μM, 1 h) exposure of U2OS cells did not significantly reduce resection (BrdU intensities) compared to Control (luciferase) siRNA treatment. Quantifications were normalized to the camptothecin treated Control siRNA treatments (CtIP depletion was the positive control; mean ± s.e.m., n=3). (J) USP4 or SART3 siRNA treatments did not noticeably affect canonical resection factor protein levels compared to Control (luciferase) siRNA treatment (*$P < 0.05$, **$P < 0.01$, ***$P < 0.001$).

Figure S3 (Related to Figure 3)

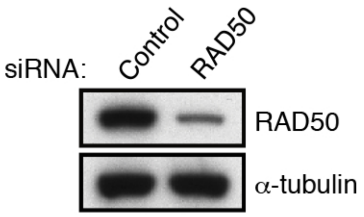

Figure S3 (Related to Figure 3)
Treatment with RAD50 siRNAs depleted RAD50 from U2OS cells. Luciferase (Control) siRNA treatment was the control.

Figure S4 (Related to Figure 4)

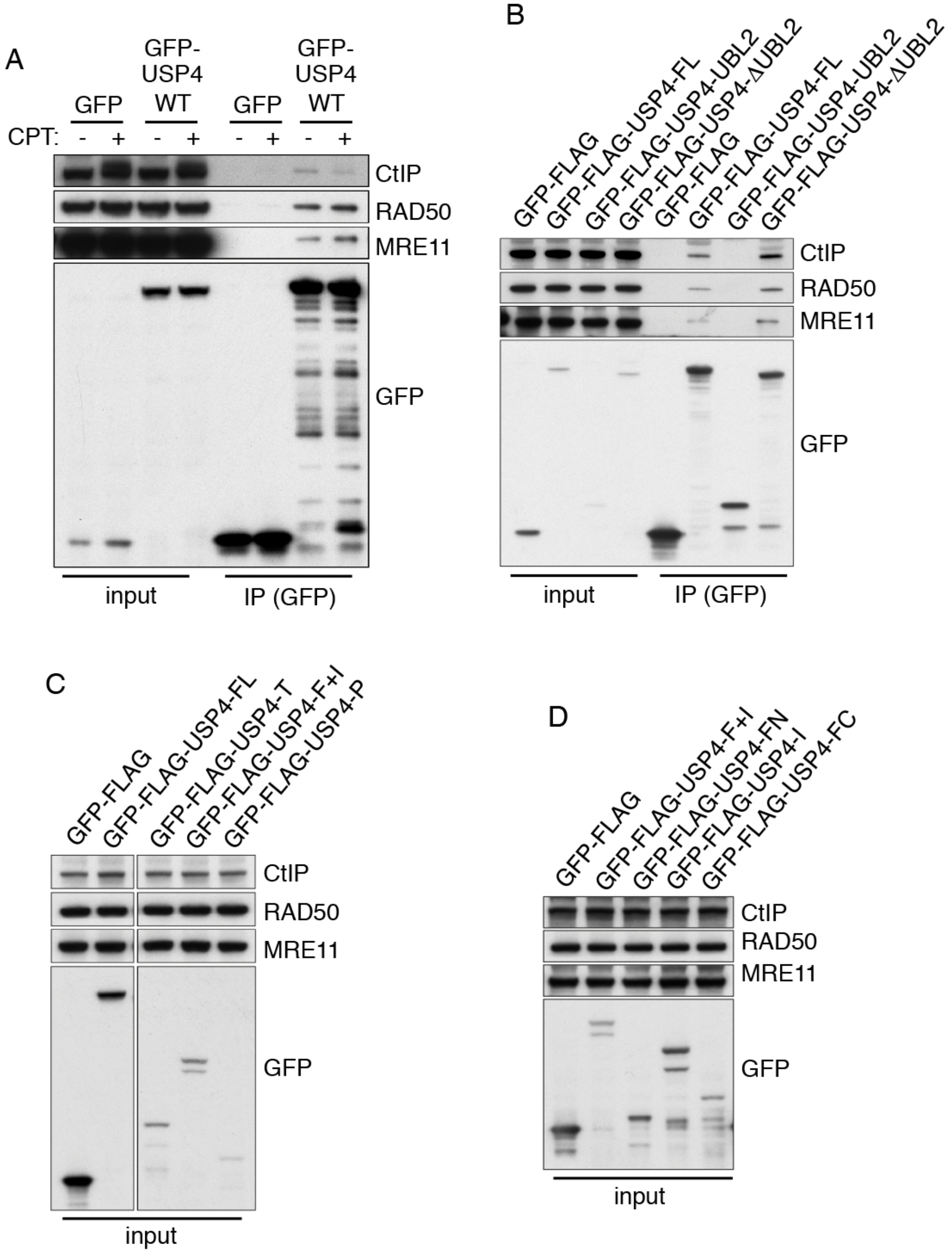

Figure S4 (Related to Figure 4)

(A) GFP-USP4 WT immunoprecipitations retrieved CtIP, RAD50 and MRE11 independent of DNA damage. GFP IP-western blot analysis with indicated antibodies, of untreated or camptothecin (CPT; 1 μM for 1 h) treated U2OS cells transiently expressing GFP or GFP-USP4 WT. (B) The USP4-UBL2 region was not necessary for the USP4-CtIP/MRN interactions. GFP immunoprecipitations and western blot analysis with the indicated antibodies, from protein lysates of U2OS cells transiently expressing GFP-FLAG, full length (FL) GFP-FLAG-USP4 or GFP-FLAG-fused USP4 mutants (see Figure 4E and Table S3 for descriptions of the USP4 truncation mutants). (C, D) Input fractions corresponding to (C) Figure 4F or (D) Figure 4G, respectively.

Figure S5 (Related to Figure 5)

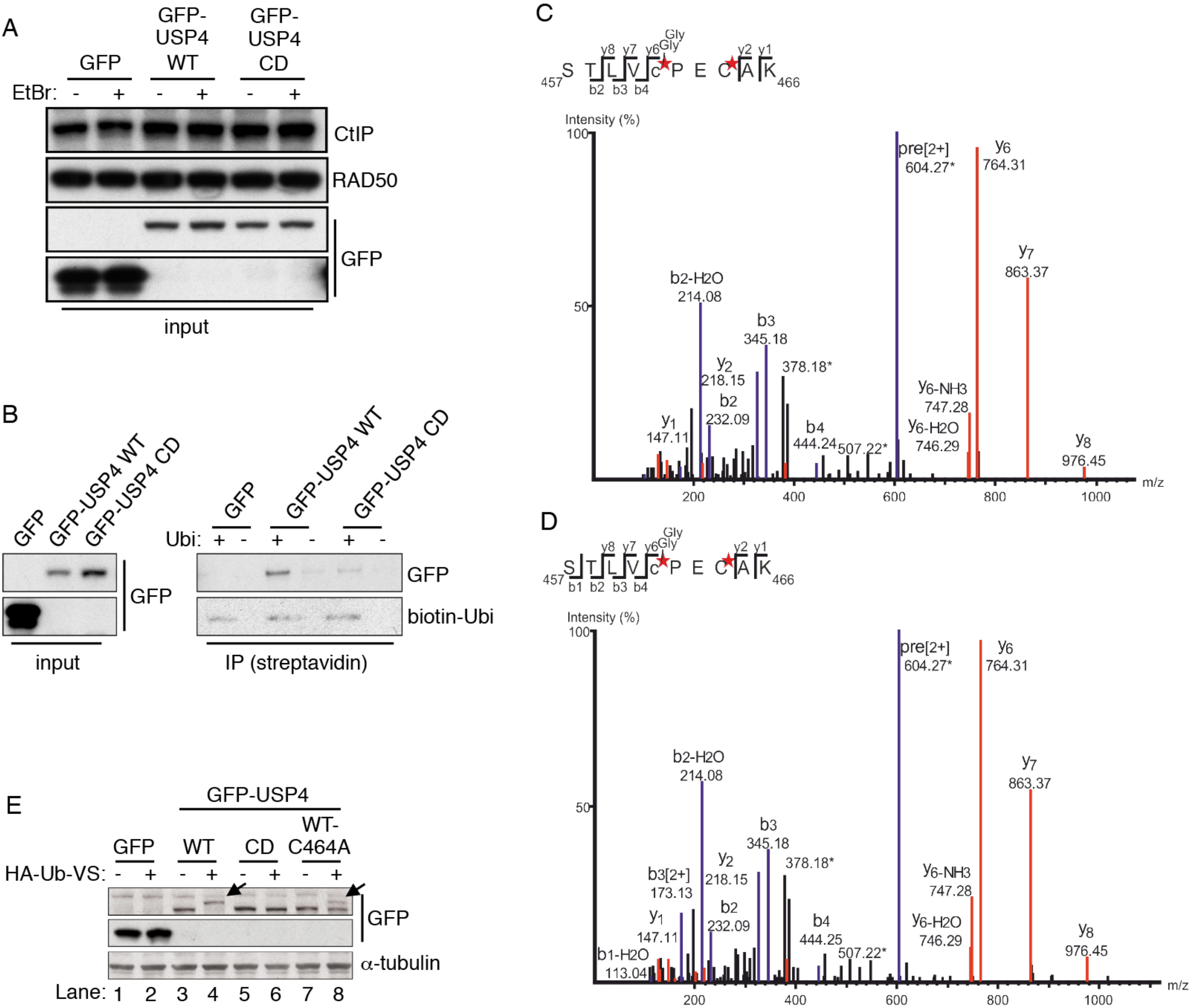

Figure S5 (Related to Figure 5)

(A) Input fractions corresponding to Figure 5A. (B) Immobilized ubiquitin retrieved GFP-USP4 WT and CD. Human amino-terminally biotinylated ubiquitin (Biotin-Ubi) was immobilized onto streptavidin Dynabeads, incubated in lysates from U2OS cells expressing GFP, GFP-USP4 WT or CD and analyzed by western blotting with the indicated antibodies. (C, D) Mass spectrometry and PEAKS analysis identified modified Cys-461 and/or Cys-464 residues suggesting possible cysteine ubiquitylations on (C) USP4 CD and (D) USP4 WT. Samples were prepared by GFP-immunoprecipitation of lysates from HEK293FT cells that transiently expressed GFP-USP4 CD or GFP-USP4 WT. The ensuing eluted material was subjected to proteolytic digestion and mass spectrometry analysis. MS/MS spectra were analyzed using PEAKS (Version 7, Bioinformatics Solutions) and indicated a match to the $_{457}$STLVCPECAK$_{466}$ peptide of USP4 (UniProt Nr Q13107). b- and y-ions are marked in blue and red, respectively, indicating a GlyGly modification either at Cys-461 or Cys-464. Asterisks indicate an alternative interpretation, assigning the y5/y4/y3 peaks as carbamidomethylation (57.02) on Cys 461 and Cys 464. Further characterization (including relative quantitation) failed, because when the carbamidomethylation step was omitted, the digestion performed with or without reduction by dithiothreitol (DTT), or when iodoacetamide was replaced by alternative alkylation reagents such as N-ethylmaleimide, this did not lead to the detection of the unmodified peptide counterpart, and further modification assignments were inconclusive. (E) USP4 Cys-464 to alanine mutation decreased but did not abolish the USP4 enzymatic activity. HA-ubiquitin probe binding assay and subsequent western blot analysis with the indicated antibodies of U2OS cells transiently expressing GFP, GFP-USP4 WT, CD or WT-C464A.

Figure S6 (Related to Figure 6)

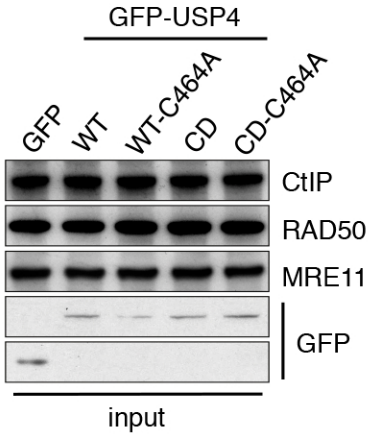

Figure S6 (Related to Figure 6)
Input fractions that correspond to Figure 6A.

Figure S7 (Related to Figure 7)

A

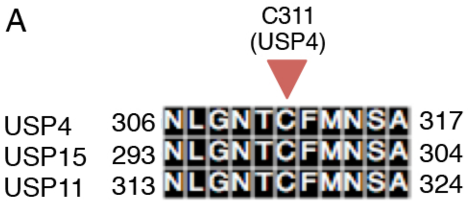

B

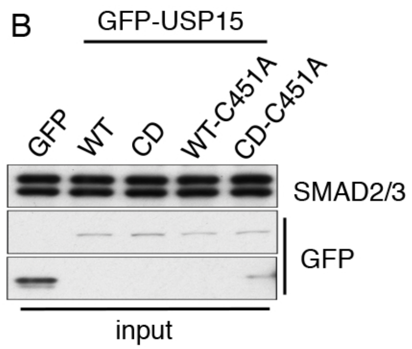

Figure S7 (Related to Figure 7)
(A) Multiple sequence alignments showing the evolutionary conserved catalytic cysteine residues of USP4, USP15 and USP11. (B) Input fractions corresponding to Figure 7D.

Supplemental Tables

Table S1 (Related to Figures 1-3 and 6): siRNAs Used in This Study

siRNA	Sequence	Supplier
siControl (luciferase)	5' AACGUACGCGGAAUACUUCGA '3	Eurofins
siUSP4-1	5' CAGGCAGACCTTGCAGTCAAA '3	Eurofins
siUSP4-2	5' CACCTACGAGCAGTTGAGCAA '3	Eurofins
siUSP4-3	5' ACCGAGGCGTGGAATAAACTA '3	Qiagen
siUSP4-4	5' TAGATGAATTAAGACGGTTAA '3	Qiagen
siUSP4-UTR	5' UUAAACAGGUGGUGAGAAA '3	Eurofins
siCtIP	5' GCUAAAACAGGAACGAAUC '3	Eurofins
siLigase IV	5' AGGAAGUAUUCUCAGGAAUUA '3	Eurofins
siXRCC4	5' AUAUGUUGGUGAACUGAGA '3	Eurofins
siBRCA1	5' GGAACCUGUCUCCACAAG '3	Eurofins
siRAD50	5' CUGCGACUUGCUCCAGAUAAA '3	Eurofins
siSART3-1	5' GGAGACAGGAAAUGCCUUA '3	Eurofins
siSART3-2	5' GAUGUGGUGUCCUGAGAUA '3	Eurofins

[a]USP4 siRNA pool (siUSP4-pool; see Figure 1A and 1B) consisted of pooled USPs 4-1, 4-2, 4-3 and 4-4 siRNAs.

Table S2 (Related to Figures 1-7): Antibodies Used in This Study

Antibody target	Supplier	Catalogue no.	Clone no.	Application	Dilution
USP4	Bethyl	A300-830A		WB	1,000
α-tubulin	Sigma	T9026		WB	1,000
Flag	Sigma	F7425		WB	1,000
GFP	Roche	118144600001		WB	1,000
CtIP	(Yu and Baer, 2000)			WB/IF	100/7.5
Ligase IV	(Riballo et al., 1999)			WB	3,000
XRCC4	Abcam	ab145		WB	500
SART3	Bethyl	A301-521A		WB	1,000
γH2AX	Millipore	05-636		WB/IF	1,000/100
RAD51	Santa Cruz	sc-8349	H-92	IF	100
RPA2 (pS4/pS8)	Bethyl	A300-245A		WB	10,000
γH2AX	Cell Signaling	2577		IF	100
RPA2	Abcam	ab2175		WB	1,000
H2AX	Abcam	ab11175		WB	5,000
BrdU	Amersham	RPN20AB		IF	200
MRE11	Genetex	GTX70212/ab214		WB	1,000
RAD50	Genetex	GTX70228/ab89		WB	1,000
NBS1	NovusBio	NB100-143		IF	800
ATM	Abcam	ab32420		WB	1000
BRCA1	Santa Cruz	sc-642		WB	500
ATR	Santa Cruz	sc-1887	N-19	WB	200
USP4	Bethyl	A300-829A		IP	1 μl/mg
USP4	Santa Cruz	sc-376000	H-3	WB	250
Cyclin A	Santa Cruz	sc-751	H432	IF	500
Cyclin A	BD	611268		IF	200
HA	CRUK	12CA5	12CA5	WB	2,000
SMAD2/3	Cell Signaling	8685	D7G7	WB	1,000
Goat anti-rabbit IgG HRP	Fisher	31462		WB	5,000
Goat anti-rabbit IgG HRP	Dako	P0260		WB	5,000
AF 594 (Goat anti-rabbit IgG)	Probes	A11037		IF	1,000
AF 488 (goat anti-rabbit IgG)	Probes	A11034		IF	1,000
AF 594 (Goat anti-mouse IgG)	Probes	A11032		IF	1,000
AF 488 (Goat anti-mouse IgG)	Probes	A11029		IF	1,000
IRDye 680CW Donkey anti (M)	Licor	926-32222		WB	25,000
IRDye 800CW Donkey anti (R)	Licor	926-32213		WB	25,000

[a]Abbreviations: WB = western blotting, IF = immunofluorescence, IP = immunoprecipitation, AF = Alexa-Fluor, M = mouse, R = rabbit.

Table S3 (Related to Figures 1, 2 and 4-7): Plasmids Used in This Study

Plasmid	Gene	Amino-acid(s)	Alteration
GFP	-	-	-
GFP-USP4 WT	USP4	-	-
GFP-USP4 CD	USP4	Cys-311	Ala
FLAG-Tev-Strep	-	-	-
FLAG-(Tev-Strep)-USP4 WT	USP4	-	-
GFP-FLAG-MRE11	MRE11	-	-
GFP-CtIP	CtIP	-	-
GFP-FLAG	-	-	-
GFP-FLAG-USP4-FL	USP4	-	-
GFP-FLAG-USP4-ΔUBL2	USP4	484-571	deleted
GFP-FLAG-USP4-UBL2	USP4	484-571	expressed
GFP-FLAG-USP4-ΔN	USP4	1-307	deleted
GFP-FLAG-USP4-N	USP4	226-963	deleted
GFP-FLAG-USP4-T	USP4	308-444	expressed
GFP-FLAG-USP4-F+I	USP4	444-862/484-571	expressed/deleted
GFP-FLAG-USP4-P	USP4	863-927	expressed
GFP-FLAG-USP4-FC	USP4	444-483	expressed
GFP-FLAG-USP4-I	USP4	572-773	expressed
GFP-FLAG-USP4-FN	USP4	774-862	expressed
HA-Ub	ubiquitin	-	-
GFP-USP4 WT-C464A	USP4	Cys-464	Ala
GFP-USP4 CD-C464A	USP4	Cys-311/Cys-464	Ala/Ala
GFP-USP4 CD-C461A	USP4	Cys-311/Cys-461	Ala/Ala
GFP-USP4 CD-C799A	USP4	Cys-311/Cys-799	Ala/Ala
GFP-USP4 CD-C802A	USP4	Cys-311/Cys-802	Ala/Ala
GFP-USP15 WT	USP15	-	-
GFP-USP15 CD	USP15	Cys-298	Ala
GFP-USP15 WT-C451A	USP15	Cys-451	Ala
GFP-USP15 CD-C451A	USP15	Cys-298/Cys-451	Ala/Ala
GFP-USP11 WT	-	-	-
GFP-USP11 CD	USP11	Cys-318	Ala

[a]Regions with the corresponding alterations are indicated in column three and four, respectively.
[b]Abbreviations: GFP = green fluorescent protein, WT = wild-type, CD = catalytically-dead, FLAG = FLAG epitope, Tev = tev protease recognition site, Strep = streptavidin epitope, FL = full-length, UBL2 = ubiquitin-like domain 2, N = N-terminal 308 amino-acids of USP4, T = tumb domain, F = fingers domain, I = insert domain, P = palm domain, FC is C-terminal part of fingers domain, FN = N-terminal part of fingers domain, HA-Ub = hemagglutinin epitope tagged ubiquitin.
[c]Amino-acid substitutions are described as one letter abbreviations in column one and three letter abbreviations in column three and four.

Table S4 (Related to Figure 5): Ubiquitin Sites Identified on USP4 WT or CD by Tandem Mass Spectrometry

USP4 CD	USP4 WT	USP4 CD	USP4 WT
Lys-47	ND	Ser-315	Ser-315
Ser-72	Ser-72	ND	Thr-323
ND	Lys-132	ND	Thr-458
Lys-158	ND	Lys-588	ND
Thr-161	Thr161	Ser-589	ND
Lys-167	ND	Thr-594	ND
Lys-171	ND	Ser-714	Ser-714
**Lys-186**	ND	Lys-770	ND
Thr-201	ND	Lys-773	ND
Ser-231	ND	ND	**Lys-811**
Lys-232	ND	**Lys-837**	ND
Ser-247	ND	Ser-900	ND
ND	Ser-256	Thr-943	Thr-943

[a]USP4 ubiquitylations identified by mass spectrometry of GFP antibody immunoprecipitations from HEK293FT lysates, which do not include the cysteine ubiquitylations described in Figure S5C and S5D.

[b]Amino-acid three letter abbreviations of ubiquitylation sites detected on USP4 WT and CD are indicated. ND = not detected. Bold lettering indicates previously reported human USP4 ubiquitylation (Kim et al., 2011; Mertins et al., 2013; Wagner et al., 2011).

Supplemental Experimental Procedures

Cell Lines and Cell Culture

Human osteosarcoma U2OS cells were cultured at 37 °C in a humidified atmosphere and 5% (v/v) CO_2 in Dulbecco's modified eagle's medium (DMEM; Sigma-Aldrich) supplemented with 10% (v/v) fetal bovine serum (FBS; Life Technologies), 100 U/ml penicillin, 100 μg/ml streptomycin and 292 μg/ml L-glutamine (Life Technologies). SV40 virus T-antigen containing human embryonic kidney (HEK) 293FT cells or U2OS cells stably expressing GFP, GFP-USP4 WT(L), WT(H), CD, WT-C464A, CD-C464A, GFP-Flag-MRE11, FLAG-Tev-Strep-USP4 WT and CD were cultured in the presence of 0.5 mg/ml geneticin (Life Technologies). U2OS cells stably expressing direct-repeat (DR)-GFP were cultured in the presence of 1 μg/ml puromycin (Sigma-Aldrich) and were established according to a previously described method (Pierce et al., 1999).

Plasmids

All expression plasmids are listed in Table S3 and all oligo-nucleotides used to produce mutants of various genes in this study, were provided by Eurofins MWG Operon. GFP-fused USP4 WT (NM_003363), USP15 WT (NP_006304.1) and USP11 WT (NM_004651), were cloned in pEGFP-GW-JJ and were described previously (Nishi et al., 2014). Catalytically-dead mutants of USP4, USP15 and USP11 and zinc-binding domain mutants of USP4 and USP15 were generated by QuickChange Lightning site-directed mutagenesis according to the manufacturer's instructions (Agilent Technologiexs). MRE11 (NM_005590) was previously cloned in pEGFP-C1-FlagN by NotI-XmaI (NEB) digestion and ligation. Construction of pcDNA3.1-FLAG-Tev-Strep was done in two steps: First, fragment A, created by annealing oligo-nucleotide one (5'-CTA GCA CCA TGG ACT ACA AAG ACC ACG ACG GAG ACT ACA AAG ACC A-3') and two (5'-AGC TTT TTT GTT GGT ATT GCT AGT TCT CCT TGG AAG TAT AGG TTT T-3') was cloned into pcDNA3.1 by Nhe1-HindIII (NEB) digestion and ligation. Fragment B, created by annealing oligo-nucleotide three (5'-AGC TCG AAA ACC TAT ACT TCC AAA GCA GCG CAT GGA GCC ACC CAC A-3') and four 5'-GAT CCT TTT TCG AAT TGT GGG TGG CTC CAG CTT CCT CCT CCG CTT C-3') was cloned into pcDNA3.1-fragment A, by HindIII-BamHI (NEB) digestion and ligation. USP4 was sub-cloned into the pcDNA3.1-FLAG-Tev-Strep plasmid by BamHI-XhoI (NEB) digestion and ligation where pEGFP-GW-JJ-USP4 was used as the DNA template. Full length (FL) USP4 and the deletion mutants used in this study were cloned into pEGFP-C1-FLAGN after KpnI-BamHI digestion and ligation. The GFP-CtIP plasmid used in this study was described previously (Sartori et al., 2007). The HA-ubiquitin PCR product from oligonucleotide combination 5'-GGG GAT CCT CAA CCA CCT CTT AGT CTT AAG ACA-3' and 5'-GGG CTA GCA TGT ACC CAT ACG ATG TTC CAG ATT ACG CTC ATA TGC AGA TCT TCG TCA AGA CGT TAA-3' was cloned into pcDNA3.1 by BamHI and NheI (NEB) digestion and ligation.

siRNA Treatment

U2OS cells (4.8 x 10^5 cells/6 cm dish) were transfected with 30 nM siRNA (see Table S1 for siRNAs used in this study) using HiPerFect (Qiagen) transfection reagent according to the manufacturer's instruction, followed 24 hours later by a second identical transfection. Treatments and analyses were performed 72 hours after initial transfection.

Cell Extract Preparation, SDS-PAGE and Western Blot Analysis

Cells were lysed at 4 °C for 30 minutes with Cytoskeleton (CSK)-buffer [300 mM sucrose (Sigma), 3 mM $MgCl_2$ (Sigma), 10 mM piperazine-N,N′-bis(2-ethanesulfonic acid) [PIPES; pH 6.8 (Sigma)], 1 mM ethylene glycol tetra-acetic acid (EGTA), 0.1% Triton X-100 (Sigma), 1x EDTA free protease inhibitor cocktail (Roche), 1x phosphatase inhibitor cocktail (Sigma), 250 μM phenylmethylsulfonyl fluoride (PMSF; Sigma) and 10 mM N-Ethylmaleimide (NEM; Sigma)] containing 300 mM NaCl (Sigma). Soluble and chromatin fractions were separated by centrifugation (20,000 x g for 10 minutes at 4 °C). Chromatin fractions were sonicated at 4 °C (30% amplitude; Sonics Vibra Cell, VHX 500 Watt) and protein concentrations were determined by Bradford protein assays (Thermo Scientific). Ten micrograms of soluble or the corresponding amount of chromatin fractions were boiled for five minutes at 95 °C in loading-buffer [67 mM Tris/HCl (pH 6.8), 2% (w/v) SDS (MP Biomedicals). 10% (v/v) Glycerol (Sigma) and 0.002% (w/v) Bromophenol Blue (Sigma) and 20% (v/v) β−mercaptoethanol (βME) in milliQ water], loaded on 4-12% Bis/Tris (Invitrogen) or Tris acrylamide gels [375 mM Tris (pH 8.8), various acrylamide/bis-acrylamide (37.5:1; Geneflow) concentrations, 0.1% (m/v) SDS, 1% (m/v) ammonium persulfate (APS) and 0.1% (v/v) tetramethylethylenediamine (TEMED)] and separated at 120 V for two hours with NuPAGE® MOPS SDS Running Buffer (Invitrogen) or Tris/glycine buffer [25 mM Tris, 191 mM glycine (Sigma) and 0.1% (m/v) SDS], respectively. After separation the proteins were transferred (350 mA, 1.5 h) onto nitro-cellulose membranes (Millipore), using the Biorad blotting systems according to the manufacturer's instructions, in Tris/Glycine buffer containing 10% (v/v) Methanol (Normapur). Nitrocellulose membranes were then blocked in Tris-buffered saline containing 0.1% (v/v) Tween-20 (TBS-T; Sigma-Aldrich) containing 1% (w/v) bovine serum albumin (BSA; Sigma-Aldrich) and incubated with various primary and secondary antibodies summarized in Table S2.

Neutral Comet Assay

U2OS cells were treated with phleomycin (40 μg/ml; Sigma) for two hours. Cells were then washed twice with phosphate buffered saline (PBS) and left to recover for two hours under normal cell culture conditions. Cells were then washed once with PBS [pH 7.5, lacking $CaCl_2$ and $MgCl_2$ (-/-); Life Technologies] and scraped off in 500 μl PBS (-/-), after which the pellets were re-suspended in PBS (-/-) at an approximate concentration of 5 x 10^6 cells/ml. Cell suspensions (10 μl) were mixed with 90 μl LMAgarose (37 °C; Trevigen) and 70 μl was spotted onto GelBond Films (Lonza), covered with 22 mm cover glasses (VWR) and incubated at 4 °C for 10 minutes. Cover glasses were removed and samples were incubated in Trevigen lysis solution for one hour at 4 °C. The lysis solution was then washed off with TBE [90 mM Tris-Borate, pH 8.3 and 2 mM 2,2',2'',2'''-(Ethane-1,2-diyldinitrilo)tetraacetic acid (EDTA)] and samples were exposed to a 35 V current for seven minutes in TBE at 22 °C. After fixation in 70% (v/v) ethanol for five minutes at 22 °C, the samples were dried for approximately 16 hours and stained with SYBER green nucleic acid staining solution (Invitrogen). Pictures were taken with an inverted Olympus TH4-200 microscope, connected to a Lumen2000 Prior stage, a FView soft imaging camera and Cell^F analysis imaging software. Per condition, the tail moments of approximately 50 individual cells were quantified with CometScore software (Tritek Corp.). Tail moments – product of tail length and fraction of total DNA in tails – of recovered cells were normalized to tail moments of damaged cells that were run on the same gel bond. Tail moments of undamaged cells were used to ascertain damage had occurred.

Clonogenic Cell Survival Assay

Cells were seeded, in six well plates (NUNC) at different concentrations (250, 500 or 1000 cells/well), 48 hours after the first siRNA treatment. Twenty-four hours after seeding, cells were exposed to various acute doses of ionizing radiation (Faxitron X-Ray Corporation, Illinois, USA), or camptothecin (Sigma-Aldrich) for one hour (camptothecin was removed and the cells were washed twice with PBS). Cells were cultured for 10 to 14 days and then stained with crystal violet solution [2% (w/v) crystal violet in 10% (v/v) ethanol]. Colonies containing more than 30 cells were counted and normalized to the undamaged controls.

Live Cell Laser-Line Micro-Irradiation

U2OS cells (1.0 x 10^5 cells/dish) stably expressing GFP-USP4 WT(H) were seeded in glass-bottom dishes. Twenty-four hours later, cells were pre-sensitized with 10 μM BrdU for 24 hours and then subjected to 400 μW localized laser micro-irradiation with a 405 nm UV-A laser beam (Limoli et al., 1993). Pictures were taken before and 30 minutes after irradiation.

Immunoprecipitation

U2OS or 293FT cells, which were cultured in 15 cm dishes (NUNC) and in most cases transfected with 15 μg of various GFP-fused expression vectors (summarized in Table S3), were washed twice with PBS and re-suspended in 1 ml (U2OS) or 3 ml (HEK293FT) lysis buffer [50 mM Tris-HCl (pH 7.5), 100 mM NaCl, 1 mM $MgCl_2$, 10% (v/v) glycerol, 5 mM NaF, 0.2% Igepal CA-630 (Sigma), protease inhibitor cocktail (10x) and 10 mM NEM] containing 25 U/ml benzonase (Novagen). Lysates were incubated at 4 °C for 45 minutes whilst rotating (15 rpm, 20 cm diameter). Then, NaCl and EDTA were added to a final concentration of 200 mM and 2 mM, respectively and lysates were incubated for an extra 20 minutes at 4 °C (whilst rotating). Lysates were subsequently separated by centrifugation (20,000 x g for 10 minutes at 4 °C).

To immunoprecipitate USP4, supernatants from 293FT cells were incubated with 1 μg/mg USP4 antibody (Bethyl) or non-specific rabbit immunoglobulins (IgG) for 16 hours at 4 °C. Supernatants were subsequently incubated in the presence of 30 μl protein A Dynabeads (Life Technologies) for one hour at 4 °C and then washed six times with lysis buffer containing 200 mM NaCl and 2 mM EDTA. Beads were subsequently incubated in 1x loading buffer (diluted with lysis buffer) at 95 °C for 10 minutes and then subjected to western blot analysis as described above.

Supernatants from cells that were transfected with various GFP-fused expression vectors were immunoprecipitated using 10 μl/sample GFP-Trap®_A gta-20 beads (Cromotek) for two and up to 16 hours at 4 °C and then washed and processed as described above.

DR-GFP HR Reporter Assay

The homologous recombination (HR) repair assays using U2OS cells, stably expressing the direct-repeat (DR)-GFP reporter, were carried out based on a previously established methodology (Pierce et al., 1999). DR-GFP cells, seeded in 6 cm dishes (4.8 x 10^5 cells/dish) and treated with siRNAs for 72 hours, were transfected with 4 μg of different plasmid combinations as described below, using the TransIT-LT1 transfection reagent according to the manufacturer's protocol (Mirus Bio LLC). To induce DSBs, the restriction enzyme I-SceI was transiently expressed (3.75 μg per sample pCBA I-SceI) together with

red fluorescent protein (RFP; 0.25 μg per sample pCS2-mRFP) to control for the transfection efficiency. To control for any background GFP signal a combination of the empty pCDNA3.1 plasmids and pCS2-mRFP was transfected (3.75 μg and 0.25 μg respectively). Forty-eight hours after plasmid transfection the cells were washed twice with PBS, detached from their growth plates using 0.1% EDTA in PBS, and collected in PBS with 5% (w/v) FBS. To be able to exclude dead cells, 1 μg/ml 4',6-diamidino-2-phenylindole (DAPI) was added, and the HR efficiency was measured as the amount of GFP and RFP double positive cells normalized to the background-GFP control population, with the BD LSRFortessa cell analyzer.

DNA-End Resection (BrdU) Assay

The DNA-end resection assays were carried out based on a previously established methodology (Nishi et al., 2014). Forty-eight hours after the first siRNA treatments, U2OS cells were pulse-labelled with 30 μM BrdU for 24 hours and then treated with 1 μM camptothecin for one hour. In some cases, cells were washed twice with PBS and then left to recover under normal cell culture conditions for one hour. After camptothecin treatment cells were washed twice with PBS, scraped off and re-suspended in 500 μl PBS. Cell suspensions were fixed in ice cold 70% (v/v) ethanol and stored at -20 °C for a minimum of 16 hours. Fixed cells were washed twice with PBS-T, incubated in block-buffer (30 minutes, 22 °C) and incubated with anti-BrdU and anti-γH2AX primary antibodies and different combinations of secondary antibodies in block-buffer (summarized in Table S2), for two and one hour at 22 °C, respectively. After antibody treatments the samples were analyzed with the BD LSRFortessa cell analyzer (BD Biosciences) in the presence of 1 μg/ml DAPI.

Cell Cycle Analysis

U2OS cells were pulse-labelled with 10 μM BrdU for 30 minutes, washed twice with PBS and scraped off in 500 μl PBS. Cell suspensions were fixed in ice cold 70% (v/v) ethanol and stored for a minimum of 16 hours at -20 °C. Fixed cells were washed twice with PBS and pellets were re-suspended in 400 μl acid-solution [5 M HCl and 0.1% (v/v) Triton X-100 in MilliQ water]. After 20 minutes incubation at 22 °C, the acid was neutralized with $Na_2B_4O_7$ (4 ml, 0.1 M). Pellets were washed once with PBS-T, incubated in block-buffer for 30 minutes at 22 °C, incubated with an anti-BrdU and a secondary Alexa Fluor® 488 goat anti-mouse antibody (summarized in Table S2). The pellets were then incubated in 300 μl PI-reagent [10 μg/ml propidium iodide (Invitrogen) and 250 μg/ml RNase A (Invitrogen)] for 20 minutes at 37 °C and cells were profiled with the BD LSRFortessa cell analyzer.

Random Plasmid Integration Assay

Seventy-two hours after the initial siRNA transfection, U2OS cells were transfected with 5 μg (per 6 cm dish) linearized (by BamHI and XhoI restriction digestion) pEGFP-C1 plasmids with the TransIT-LT1 transfection reagent according to the manufacturer's instructions. Six hours after plasmid transfection, cells were seeded into 15 cm dishes at four different concentrations (1.0 x 10^3, 2.5 x 10^3, 1.0 x 10^4 or 2.0 x 10^4 cells/plate). Twenty-four hours after re-seeding, plates containing 1.0 x 10^4 or 2.0 x 10^4 cells were cultured in presence of 1 mg/ml of geneticin and the plates containing 1.0 x 10^3 or 2.5 x 10^3 cells in absence of geneticin for 10 to 14 days and subsequently stained with 2% (w/v) crystal violet solution. Plasmid integration efficiencies were analyzed as the percentage of geneticin-resistant cells normalized to the transfection efficiency [transfection efficiencies

were calculated as percentages of GFP positive cells (24 hours after plasmid transfection), assessed by flow-cytometry with the FACS Calibur (BD Biosciences)].

Immunofluorescence Staining

U2OS cells, cultured on poly-L-lysine [0.01% (w/v); Sigma-Aldrich] coated coverslips or glass bottom dishes (Willco Wells; 3.5 cm diameter and 0.17 mm glass thickness), were fixed with 2% (w/v) paraformaldehyde (PFA; Sigma-Aldrich) at 22 °C for 20 minutes and permeabilized with 0.2% (v/v) Triton X-100 at 22 °C for 15 minutes. Following incubation in block-buffer [PBS, 5% (v/v) FBS] for 30 minutes at 22 °C, fixed cells were incubated with various primary antibodies at 4 °C for 16 to 24 hours. Cells were washed twice with PBS containing 0.1% (v/v) Tween-20 (PBS-T) and incubated with various secondary antibodies at 22 °C for one to two hours. Primary and secondary antibodies are described in Table S2. Last, cells were exposed to 1 μg/ml DAPI in PBS for 10 minutes (22 °C), mounted onto glass and subsequently analyzed with an Olympus inverted confocal microscope.

EdU Labeling

Seventy-two hours after the first transfection, U2OS cells were exposed to 10 μM 5-ehtynyl-2'-deoxyuridine (EdU) for 15 minutes. Cells were then washed twice with PBS and fixed in 2% PFA in PBS (w/v) at 22 °C for 20 minutes. Subsequently, the cells were washed twice in PBS and permeabilized with 0.2% Triton-X-100 in PBS (15 minutes at 22 °C), washed once with PBS and incubated in 100 μl/sample 'Click-iT' solution [20 mg/ml (1x) Click-iT® EdU buffer, 20 mM $CuSO_4$ and 1:500 diluted Alexa Fluor 647 azide in PBS (Molecular probes)] for 30 minutes at 22 °C, under protection from the light. The EdU intensities of the S-phase cell populations were measured with the BD LSRFortessa cell analyzer.

NBS1 or CtIP Recruitment to Laser-Line Micro-Irradiation Induced DNA Lesions

Cells (1.0 x 10^5 cells/dish) were seeded in glass bottom dishes. Forty-eight hours after siRNA treatments cells were pre-sensitized with 10 μM BrdU for 24 hours and then subjected to 250 μW localized laser micro-irradiation with a 405 nm UV-A laser beam (Limoli et al., 1993). Two hours after the first cells were irradiated, the samples were processed for immunofluorescent staining as described above.

Ubiquitylation Assay

Cells were cultured in 10 cm or 15 cm dishes and transfected with 2.5 μg or 7.5 μg pcDNA3.1-HA-ubiquitin and 2.5 μg or 7.5 μg various expression vectors (summarized in Table S3), respectively. Forty-eight hours later, cells were washed twice with PBS and the pellets were lysed with CSK buffer containing 300 mM NaCl (described previously but without addition of phosphatase inhibitor or PMSF). Supernatants were immunoprecipitated using EZview™ RED-Anti-HA affinity beads (Sigma) for two hours and washed six times with CSK buffer. Beads were then incubated at 95 °C for 10 minutes in loading buffer either in presence or absence of βME and subjected to western blot analysis as described above.

Sample Preparation for Mass Spectrometry

Immunoprecipitated and eluted protein materials were precipitated and desalted using chloroform-methanol. Protein samples were reduced and alkylated using 20 mM DTT and 20 mM iodoacetamide as described previously (Ternette et al., 2013). Tryptic digests were

carried out overnight at 37 °C and desalting steps were performed with C18 SepPak cartridge columns according to the manufacturer's instructions (Waters). Eluted peptides were dried under vacuum and the pellets were kept at -20 °C until analysis. Pellets were re-suspended in 20 μl H_2O with 2% (v/v) acetonitrile, 0.1% formic acid (v/v) prior mass spectrometry analysis.

Tandem Mass Spectrometry and Data Analysis

Samples were analyzed with nano–liquid chromatography tandem mass spectrometry (nano–LC-MS/MS), using a Dionex Ultimate 3000 UPLC coupled to a hybrid quadrupole-orbitrap instrument (Q Exactive, Thermo). Samples were loaded onto a PepMAP C18, 300 μm x 5mm, 5 μm particle pre-column (Thermo) for one minute at a flow rate of 20 μl/min and separated on an nEASY column (PepMAP C18, 75 μm x 500 mm, 2 μm particle; Thermo) for 60 minutes using a gradient of 2%-35% acetonitrile (v/v) in 5% DMSO (v/v) and 0.1% formic acid (v/v) at 250 nl/min. Scans were performed at a resolution of 70,000 at 200 mass/charge and the 15 most abundant precursors were selected for Higher-energy Collisional Dissociation (HCD) fragmentation.

Peak lists containing MS/MS spectra were generated using MSConvert (ProteoWizard 3.0.4743), keeping the 200 most intense peaks. These lists were then searched using Mascot version 2.5.1 (http://www.matrixscience.com) against the Swiss-Prot protein database with the taxonomy restriction "human" (20,353 entries as of July 2014) with tryptic enzymatic restriction and with mass deviations of 10 parts per million/0.05 daltons in MS and MS/MS mode, respectively. Oxidation of methionine, deamidation of asparagine and glutamine, and GlyGly (C/T/S/K) were searched for as variable modifications. In addition, the same data sets were analyzed using PEAKS (Version 7, Bioinformatics Solutions) software. Raw MS data were *de novo* sequenced by PEAKS using HCD fragmentation data. A database search (SwissProt, 20,204 human sequences) with subsequent posttranslational modification (PTM) searches, where all modifications reported in UNIMOD were considered, was then applied to the *de novo* identified MS/MS spectra. False discovery rates (FDR) of 1% threshold were applied. Peptide MS/MS spectra including GlyGly modifications were inspected manually.

Biotin-Ubiquitin Binding Assay

Cells (293FT) were cultured in 15 cm dishes and transfected with 15 μg plasmid [GFP or GFP-fused USP4 derivatives (summarized in Table S3)]. Forty-eight hours later, cells were processed to create protein extracts using CSK buffer containing 300 mM NaCl and without phosphatase inhibitor, PMSF or NEM, according to the protocol described above. Meanwhile, streptavidin M-280 Dynabeads (Life Technologies) were soaked in an excess of Biotin-fused human recombinant ubiquitin (R&D Systems) for one hour at 4 ºC, after which 10 μl ubiquitin-coupled streptavidin was added to the lysates, incubated for 16 hours at 4 ºC, washed six times in CSK buffer containing 300 mM NaCl, incubated at 95 °C for 10 minutes in loading buffer and subjected to western blot analysis as described previously.

Active Probe Binding Assay

Cell extracts were lysed (30 min at 4 ºC) with NP lysis buffer [50 mM Tris-HCl (pH 7.5), 150 mM NaCl, 3 mM EDTA and 0.5% NP-40] containing 1x EDTA free protease inhibitor, 1x phosphatase inhibitor, 1 mM DTT. Supernatants were prepared by centrifugation at

20,000x g for 10 min at 4 ºC. To 20 μg supernatant, 250 ng HA-Ub-VS probe [vinyl sulfone, (HA-tag); Enzo Life Sciences, (Borodovsky et al., 2002)] was added and incubated at 37 ºC for two hours. Samples were boiled for 10 minutes at 95 °C in loading-buffer to inactivate the reaction and prepare for SDS-PAGE and western blot analysis as described previously.

Supplemental References

Borodovsky, A., Ovaa, H., Kolli, N., Gan-Erdene, T., Wilkinson, K.D., Ploegh, H.L., and Kessler, B.M. (2002). Chemistry-based functional proteomics reveals novel members of the deubiquitinating enzyme family. Chem Biol *9*, 1149–1159.

Kim, W., Bennett, E.J., Huttlin, E.L., Guo, A., Li, J., Possemato, A., Sowa, M.E., Rad, R., Rush, J., Comb, M.J., et al. (2011). Systematic and quantitative assessment of the ubiquitin-modified proteome. Molecular Cell *44*, 325–340.

Larrieu, D., Britton, S., Demir, M., Rodriguez, R., and Jackson, S.P. (2014). Chemical inhibition of NAT10 corrects defects of laminopathic cells. Science *344*, 527–532.

Limoli, C.L., Limoli, C.L., Ward, J.F., and Ward, J.F. (1993). A new method for introducing double-strand breaks into cellular DNA. Radiat. Res. *134*, 160–169.

Mertins, P., Qiao, J.W., Patel, J., Udeshi, N.D., Clauser, K.R., Mani, D.R., Burgess, M.W., Gillette, M.A., Jaffe, J.D., and Carr, S.A. (2013). Integrated proteomic analysis of post-translational modifications by serial enrichment. Nat. Methods *10*, 634–637.

Nishi, R., Wijnhoven, P., le Sage, C., Tjeertes, J., Galanty, Y., Forment, J.V., Clague, M.J., Urbé, S., and Jackson, S.P. (2014). Systematic characterization of deubiquitylating enzymes for roles in maintaining genome integrity. Nat. Cell Biol. *16*, 1016–26–1–8.

Pierce, A.J., Johnson, R.D., Thompson, L.H., and Jasin, M. (1999). XRCC3 promotes homology-directed repair of DNA damage in mammalian cells. Genes & Development *13*, 2633–2638.

Riballo, E., Critchlow, S.E., Teo, S.H., Doherty, A.J., Priestley, A., Broughton, B., Kysela, B., Beamish, H., Plowman, N., Arlett, C.F., et al. (1999). Identification of a defect in DNA ligase IV in a radiosensitive leukaemia patient. Curr Biol *9*, 699–702.

Sartori, A.A., Lukas, C., Coates, J., Mistrik, M., Fu, S., Bartek, J., Baer, R., Lukas, J., and Jackson, S.P. (2007). Human CtIP promotes DNA end resection. Nature *450*, 509–514.

Ternette, N., Yang, M., Laroyia, M., Kitagawa, M., O'Flaherty, L., Wolhulter, K., Igarashi, K., Saito, K., Kato, K., Fischer, R., et al. (2013). Inhibition of mitochondrial aconitase by succination in fumarate hydratase deficiency. Cell Reports *3*, 689–700.

Wagner, S.A., Beli, P., Weinert, B.T., Nielsen, M.L., Cox, J., Mann, M., and Choudhary, C. (2011). A proteome-wide, quantitative survey of in vivo ubiquitylation sites reveals widespread regulatory roles. Mol. Cell Proteomics *10*, M111.013284.

Yu, X., and Baer, R. (2000). Nuclear localization and cell cycle-specific expression of CtIP, a protein that associates with the BRCA1 tumor suppressor. J Biol Chem *275*, 18541–18549.
