# Supplementary material for: USP4 Auto-Deubiquitylation Promotes Homologous Recombination
Source: Mol Cell. 2015 Nov 5;60(3):362–73. doi: 10.1016/j.molcel.2015.09.019 (PMC4643306; doi:10.1016/j.molcel.2015.09.019)
Supplement: Document S2. Article plus Supplemental Information [file mmc2.pdf]

Molecular Cell

Article

USP4 Auto-Deubiquitylation Promotes Homologous Recombination

Graphical Abstract

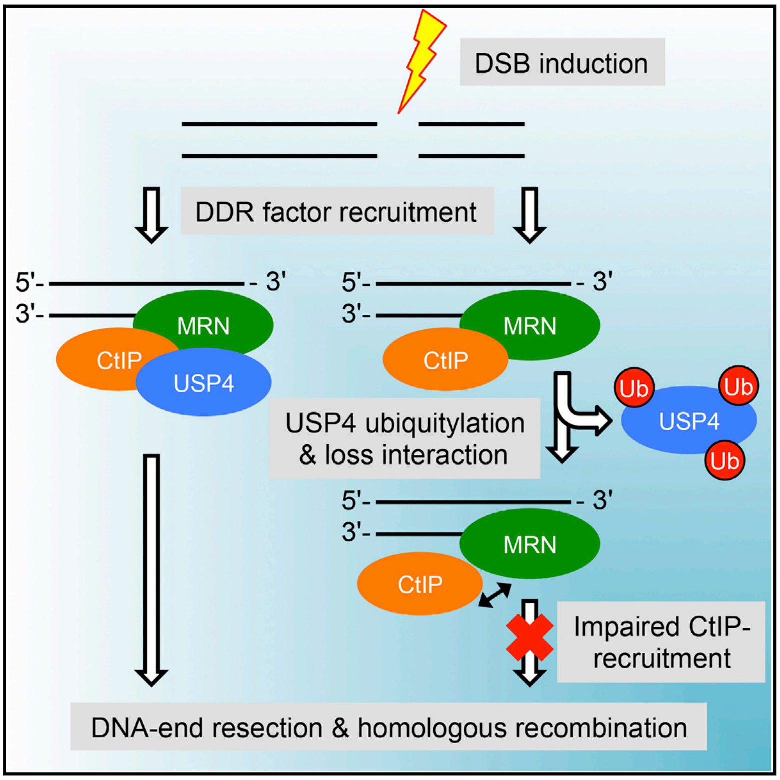

Authors

Paul Wijnhoven, Rebecca Konietzny, Andrew N. Blackford, Jonathan Travers, Benedikt M. Kessler, Ryotaro Nishi, Stephen P. Jackson

Correspondence

rnishi@fc.ritsumei.ac.jp (R.N.),
s.jackson@gurdon.cam.ac.uk (S.P.J.)

In Brief

Wijnhoven et al. revealed that the human deubiquitylase USP4 promotes homologous recombination, DNA end-resection, and CtIP recruitment and that USP4 interacts with CtIP and MRN. Wijnhoven et al. also showed that such interactions and DDR processes are stimulated by USP4 auto-deubiquitylation on multiple sites, including those on cysteine residues.

Highlights

- The deubiquitylating enzyme USP4 promotes homologous recombination-mediated repair
- USP4 regulates recruitment and/or association dynamics of CtIP at DNA damage sites
- Auto-deubiquitylation of USP4 stimulates HR events and interactions with CtIP/MRN
- Ubiquitin adducts might regulate USP-enzyme interactions/ function more commonly

Wijnhoven et al., 2015, Molecular Cell *60*, 362–373
November 5, 2015 ©2015 The Authors
http://dx.doi.org/10.1016/j.molcel.2015.09.019

Molecular Cell
Article

USP4 Auto-Deubiquitylation Promotes Homologous Recombination

Paul Wijnhoven,[1] Rebecca Konietzny,[2] Andrew N. Blackford,[1] Jonathan Travers,[1,4] Benedikt M. Kessler,[2,6] Ryotaro Nishi,[1,5,*] and Stephen P. Jackson[1,3,6,*]
[1]The Wellcome Trust/Cancer Research UK Gurdon Institute, University of Cambridge, Cambridge CB2 1QN, UK
[2]Target Discovery Institute, Nuffield Department of Medicine, University of Oxford, Oxford OX3 7FZ, UK
[3]The Wellcome Trust Sanger Institute, Hinxton CB10 1SA, UK
[4]Present address: MedImmune, Sir Aaron Klug Building, Granta Park, Cambridge CB21 6GH, UK
[5]Present address: Department of Biomedical Sciences, College of Life Sciences, Ritsumeikan University, 1-1-1 Nojihigashi, Kusatsu, Shiga, 525-8577, Japan
[6]Co-senior authors
*Correspondence: rnishi@fc.ritsumei.ac.jp (R.N.), s.jackson@gurdon.cam.ac.uk (S.P.J.)
http://dx.doi.org/10.1016/j.molcel.2015.09.019
This is an open access article under the CC BY license (http://creativecommons.org/licenses/by/4.0/).

SUMMARY

Repair of DNA double-strand breaks is crucial for maintaining genome integrity and is governed by post-translational modifications such as protein ubiquitylation. Here, we establish that the deubiquitylating enzyme USP4 promotes DNA-end resection and DNA repair by homologous recombination. We also report that USP4 interacts with CtIP and the MRE11-RAD50-NBS1 (MRN) complex and is required for CtIP recruitment to DNA damage sites. Furthermore, we show that USP4 is ubiquitylated on multiple sites including those on cysteine residues and that deubiquitylation of these sites requires USP4 catalytic activity and is required for USP4 to interact with CtIP/MRN and to promote CtIP recruitment and DNA repair. Lastly, we establish that regulation of interactor binding by ubiquitylation occurs more generally among USP-family enzymes. Our findings thus identify USP4 as a novel DNA repair regulator and invoke a model in which ubiquitin adducts regulate USP enzyme interactions and functions.

INTRODUCTION

To counteract the deleterious consequences of DNA double-strand breaks (DSBs) and other DNA lesions, multiple cellular mechanisms have evolved, collectively termed the DNA damage response (DDR) (Ciccia and Elledge, 2010; Jackson and Bartek, 2009). The DDR is tightly regulated by reversible post-translational protein modifications (PTMs). For instance, following DSB recognition by sensor proteins such as the MRE11-RAD50-NBS1 (MRN) complex that interacts with CtIP (Sartori et al., 2007), phosphorylation cascades triggered by the protein kinases ATM, ATR, and DNA-PKcs control and coordinate DSB repair and associated events. These events include phosphorylation of histone 2A variant H2AX on serine 139 (to form γH2AX) on chromatin flanking DSB sites, to which MDC1 then binds (Stucki et al., 2005), mediating recruitment of factors such as the E3 ubiquitin ligases RNF8 and RNF168, together with 53BP1 and the BRCA1-A complex, leading to chromatin remodelling in preparation for repair (Ciccia and Elledge, 2010).

Phosphorylation has been extensively studied in the context of the DDR for many years. By contrast, how ubiquitylation—the covalent attachment of the ~8.5 kDa protein ubiquitin to substrates—and related events regulate DSB repair and associated processes has only recently become the focus of intensive research. Ubiquitin is conjugated to its substrates via an enzymatic cascade involving an activating (E1), conjugating (E2) enzyme, and, in most cases an E3 ubiquitin ligase (Hershko et al., 1983); and in mammals, ubiquitylation involves two E1s, more than 35 E2s, and >600 E3 ligases (Clague et al., 2015). Substrates can be mono-ubiquitylated at more than one site and/or are polyubiquitylated by polymerization of multiple ubiquitin moieties via one or more of seven internal lysine residues (Lys-6, Lys-11, Lys-27, Lys-29, Lys-33, Lys-48, and Lys-63) within ubiquitin or with the ubiquitin amino-terminus (Komander, 2009a). These different linkages lead to ubiquitin chains with distinct structural features and functions, including those with well-established roles in the DDR (Ciccia and Elledge, 2010; Jackson and Durocher, 2013). In recent years, it has become evident that editing and removal of such ubiquitylations by deubiquitylases (DUBs) play crucial roles in regulating ubiquitylation events and the activities they control.

The human genome encodes 94 putative DUBs, classed into five groups based on structural features of their catalytic domains (Komander et al., 2009b). We recently carried out a systematic screen of DUBs for DDR functions, particularly focusing on DSB repair by non-homologous end-joining (NHEJ) or homologous recombination (HR) (Nishi et al., 2014). Among others, this work suggested DSB repair roles for members of the structurally similar ubiquitin-specific proteases USP4, USP15, and USP11 (Komander et al., 2009b), the latter being a DUB with previously established DDR roles (Schoenfeld et al., 2004; Wiltshire et al.,

2010). Notably, USP4, USP11, and USP15 are implicated in related cellular events, including TGF-β signaling (Al-Salihi et al., 2012; Inui et al., 2011; Zhang et al., 2012), raising the possibility that they might have redundant and/or complementary DDR functions.

Here, we report that USP4, a DUB with previously reported links to mitogen-activated protein (MAP) kinase signaling (e.g., Zhang et al., 2012), pre-mRNA splicing (Song et al., 2010), and control of p53 stability (Zhang et al., 2011), regulates DNA repair and cellular survival upon DSB induction. We also show that USP4 depletion impairs HR repair by affecting the process of DNA-end resection. Additionally, we establish that USP4 interacts with CtIP and the MRN complex via its C-terminal insert domain and that these interactions are subject to a USP4 autoregulatory deubiquitylation mechanism. Finally, we provide evidence that this type of control might operate more widely by showing that interactions between USP15 with SMAD2/3 (Inui et al., 2011) are subject to USP15 auto-regulation.

RESULTS

USP4 Promotes DSB Repair

We recently showed that the structurally similar proteins USP4, USP11, and USP15 have DDR roles (Nishi et al., 2014) (see Figure S1A for their domain features). Focusing on USP4, we found by neutral comet assays that its depletion from human U2OS cells by various short-interfering RNA (siRNA) oligonucleotides (Figure 1A) reduced repair of DSBs induced by the radiomimetic chemical phleomycin (Figure 1B; see Figure S1B for control CtIP and DNA ligase IV depletions). To determine whether such effects were mediated via USP4 loss, we generated stable cell lines expressing GFP fused to wild-type USP4 (GFP-USP4 WT) and selected a clone, GFP-USP4 WT(L) (see Figure S1C for this and other cell lines used in this study), where fusion protein expression was comparable to endogenous USP4 (see Figure 1C, lane 4 to compare endogenous and exogenous USP4). We then treated GFP-USP4 WT(L) cells or control cells expressing GFP alone with an siRNA targeting the USP4 coding region (USP4-2) to deplete both endogenous and GFP-fused USP4 or with an siRNA targeting the USP4 3′ UTR to deplete endogenous but not exogenous GFP-fused USP4 (Figure 1C). Ensuing comet analyses (Figure 1D) revealed that, while both siRNAs produced DSB repair defects in control cells, only the coding-region targeting siRNA yielded such a defect in cells expressing GFP-USP4 WT(L). These data thus indicated that GFP-USP4 WT expression complemented DSB repair defects induced by depleting endogenous USP4. USP4 depletion also sensitized cells to ionizing radiation (IR) (Figure 1E; see Figure S1D for depletion of the NHEJ protein XRCC4), providing additional evidence that USP4 promotes DNA repair.

Consistent with previous work (Nishi et al., 2014), we found that stably expressed GFP-fused USP4 WT accumulated at DNA damage sites generated by laser micro-irradiation (Figure S1E), suggesting that USP4 functions in proximity to DNA lesions. By contrast, we did not detect DNA-damage accumulation of GFP-fused U4/U6 recycling protein SART3 (data not shown), which interacts with USP4 and targets USP4 to its spliceosomal substrate PRP3 (Song et al., 2010). Although this might be due to detection limitations, these data suggested that USP4 might exist in multiple complexes and that its roles in DSB repair might be distinct from its spliceosomal functions. While it has been reported that overexpressed SART3 causes nuclear localization of exogenously expressed USP4 (Song et al., 2010), we found that SART3 depletion from U2OS cells did not detectably affect the nuclear/cytoplasmic distribution of endogenous USP4 (data not shown), suggesting that USP4 nuclear targeting might be mediated by multiple mechanisms.

To assess whether USP4 catalytic activity was needed for effective DSB repair, we characterized U2OS cell clones expressing similar amounts of USP4 WT (referred to as GFP-USP4 WT(H)) or GFP fused to catalytically dead USP4 (USP4 CD), where the catalytic cysteine (Cys-311) was changed to alanine (C311A; also see Figure S1C for expression levels). We then depleted endogenous USP4 from such cells by siRNA treatment (Figure 1F) and subjected them to neutral comet assays. This showed that USP4 catalytic activity was required for effective DSB repair (Figure 1G). Furthermore, during the course of these studies, we found that cells expressing USP4 CD displayed DSB repair defects even without endogenous USP4 depletion (Figure 1G; see Figure S1F for equivalent neutral comet assays without siRNA treatments), suggesting that catalytically dead USP4 behaved in a dominant-negative manner. Accordingly, U2OS cells expressing GFP-USP4 CD were more sensitive to IR than cells expressing GFP or GFP-USP4 WT (Figure 1H). By co-transfection of FLAG-epitope-tagged USP4 WT with GFP-USP4 WT or CD constructs, followed by GFP-immunoprecipitation (IP) and western blot analyses, we found that USP4 molecules interacted with one another irrespective of catalytic function (Figure 1I). We thus speculate that USP4 CD exerts its dominant-negative effects, at least in part via binding to endogenous USP4.

USP4 Functions in DNA-End Resection

Through ensuing studies, we established that USP4 depletion reduced HR-repair efficiencies in a cell-based assay measuring chromosomal DSB repair by gene conversion (Figure 2A). Importantly, these effects were observed despite the siRNA targeting the USP4 3′ UTR having little effect on the combined S/G2-phase cell population (Figure S2A; although siUSP4-2 reduced the S/G2 population by ~25% compared to the siRNA control, this is unlikely to account for the ~60% reduced HR efficiency). USP4 depletion also reduced NHEJ as assessed by a random plasmid integration assay (Figure S2B). Although USP4 involvement in NHEJ will be worth pursuing, our studies focused on its impact on HR and related events. Importantly, in line with our other studies implying that USP4 promotes DSB repair via mechanisms distinct from its spliceosomal functions in concert with SART3, HR was not significantly altered by SART3 depletion (Figure S2D; see Figure S2C for siSART3 depletion).

In accordance with USP4 functioning in HR, its depletion sensitized cells to camptothecin (Figure 2B), which yields replication-associated one-ended DSBs in S-phase that must be repaired by HR. In HR, RAD51 assemblies replace replication protein A (RPA) on resected ssDNA to form nucleoprotein filaments that mediate strand-invasion and ensuing HR events. As these RAD51 assemblies can be detected as IR-induced subnuclear foci (IRIF) (Polo and Jackson, 2011), we assessed

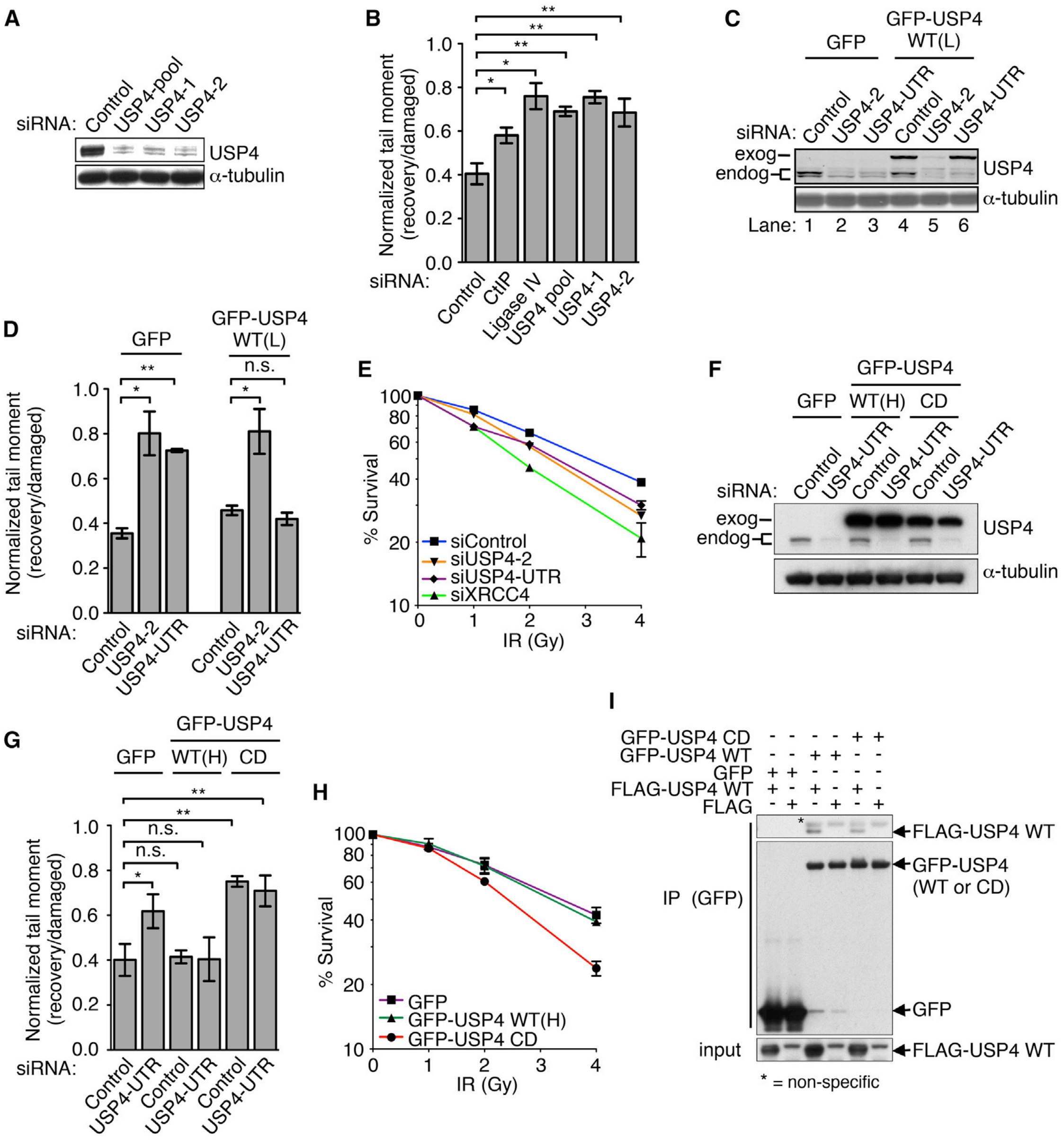

Figure 1. USP4 Promotes DSB Repair

(A) USP4-targeting siRNAs depleted USP4 from U2OS cells.

(B) USP4 depletion from U2OS cells caused DSB repair defects (neutral comet assays) after phleomycin (40 μg/ml, 2 hr) treatment, measuring the effects of depleting CtIP or DNA ligase IV, as controls (mean ± SEM; n = 3). Also see Table S1 for siRNAs and Table S2 for antibodies used in this study.

(C) Treatments of U2OS cells with USP4-2 but not USP4-UTR siRNAs depleted exogenously expressed GFP-USP4 WT(L); exog, exogenous; endog, endogenous. Also, see Table S3 for plasmids used in this study.

(D) Exogenously expressed USP4 WT(L) restored DSB repair defects (neutral comet assays) observed after USP4 depletion (mean ± SEM; n = 3).

(E) USP4 depletion sensitized U2OS cells to IR (mean ± SEM; n = 3 and XRCC4 siRNA-treated cells were the positive control).

(F) Treatment of U2OS cells with USP4-UTR siRNAs depleted endogenous USP4 but not exogenously expressed GFP-USP4 WT(H) or CD. exog, exogenous; endog, endogenous (also see Figure S1C for expression levels).

(G) Expression of GFP-USP4 CD but not WT(H) caused DSB repair defects (neutral comet assays) irrespective of endogenous USP4 depletion (mean ± SEM; n = 3).

(H) Expression of GFP-USP4 CD sensitized U2OS cells to IR (mean ± SEM; n = 3).

(I) GFP-USP4 WT and CD immunoprecipitations from U2OS cell extracts retrieved FLAG-USP4 WT (*$p < 0.05$ **$p < 0.01$; n.s., not significant). See also Figure S1.

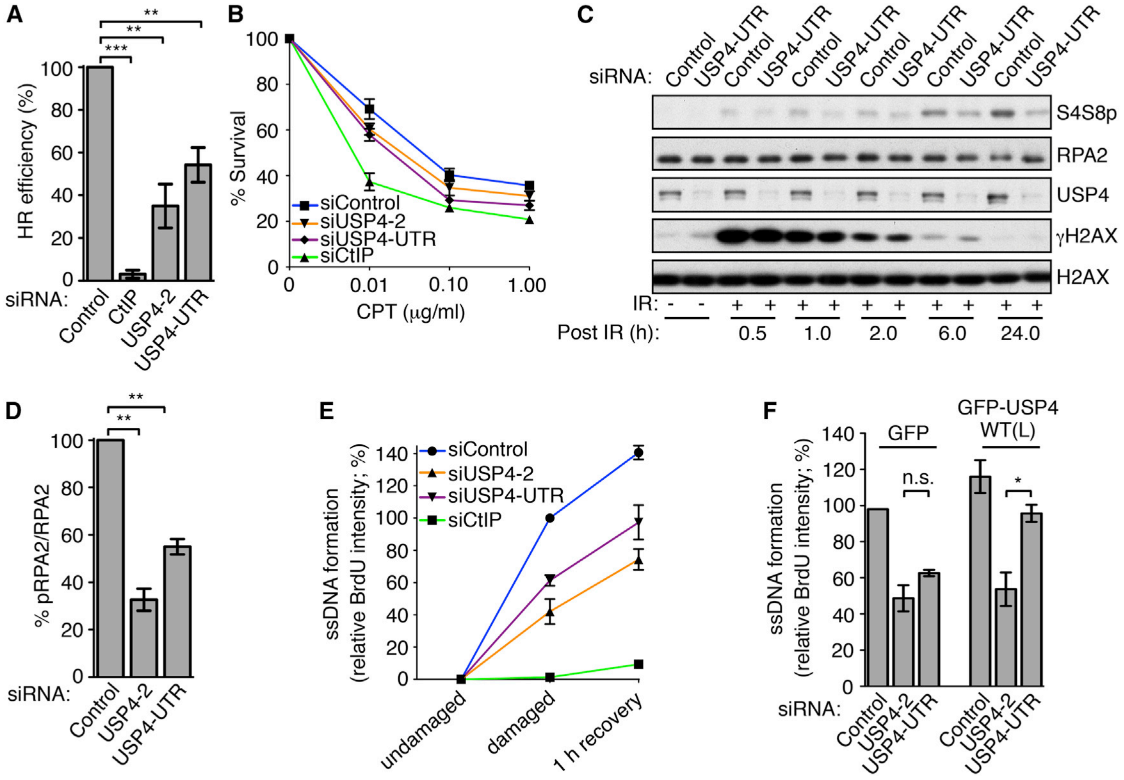

Figure 2. USP4 Functions in DNA-End Resection

(A) USP4 depletion caused HR defects [direct-repeat (DR)-GFP reporter assays]. Quantifications were normalized to control siRNA-treated cells and set to 100% (mean ± SEM; n = 4).

(B) USP4 depletion sensitized U2OS cells to camptothecin (CPT) (mean ± SEM; n = 3 and CtIP siRNA-treatment was the positive control).

(C) USP4 depletion reduced RPA2 Ser-4/Ser-8 phosphorylation (S4S8p) after IR (10 Gy).

(D) USP4 depletion reduced RPA2 S4S8p after camptothecin (1 μM, 1 hr) treatment. Intensities were quantified with Odysey CLx (LI-COR) and Image Studio 4.x software and RPA2 S4S8p was normalized to RPA2. Quantifications were normalized to the camptothecin-treated siControl and set to 100% (mean ± SEM; n = 3).

(E) USP4 siRNA treatment, followed by camptothecin (1 μM, 1 hr) treatment, of U2OS cells reduced resection (BrdU intensities). Quantifications were normalized to the camptothecin-treated siControl (CtIP depletion was the positive control; mean ± SEM; n = 3).

(F) GFP-USP4 WT(L)-complemented U2OS cells restored resection defects (BrdU intensities) observed upon USP4 depletion. Quantifications were normalized to camptothecin and Control siRNA-treated GFP cells (mean ± SEM; n = 3; *$p < 0.05$; **$p < 0.01$; ***$p < 0.001$; n.s., not significant). See also Figure S2.

whether their formation was affected by USP4. Indeed, USP4 depletion reduced the proportion of cells exhibiting RAD51 foci that co-localized with γH2AX, indicating that RAD51 loading at DSB sites was compromised (Figure S2E; BRCA1 depletion was used as control). When loaded on ssDNA, the RPA subunit RPA2 is phosphorylated on Ser-4 and Ser-8, and inhibition of all known DNA-end resection factors reduces this mark (e.g., Gravel et al., 2008; Polo et al., 2012; Sartori et al., 2007). In line with USP4 affecting resection, its depletion reduced RPA2 Ser-4/Ser-8 phosphorylation (S4S8p) at various times after IR (Figure 2C) or camptothecin exposure (Figures 2D and S2F). Importantly, γH2AX intensities after camptothecin treatment were not significantly affected when cells were treated with the siRNA targeting the USP4 3′ UTR (Figure S2F), implying that S-phase entry and progression were not markedly altered by USP4 depletion (siUSP4-2 treatments reduced S-phase cell populations by ~25% [Figure S2A], probably partially accounting for reduced γH2AX and S4S8p RPA2 after siUSP4-2 treatment [Figure S2F]). We also found that expression of GFP-USP4 WT(L) at levels similar to those of endogenous USP4, partially rescued the RPA2 phosphorylation defect caused by depleting endogenous USP4 (Figure S2G). Furthermore, pulse-labeling cells with 5-ethynyl-2′-deoxyuridine (EdU) followed by assessment of its incorporation into DNA using EdU labeling indicated that overall levels of DNA replication in S-phase cells were not significantly altered by USP4 depletion (Figure S2H). Collectively, these data strongly suggested that USP4 promotes DNA-end resection.

To more directly address resection efficiencies in S-phase cell populations, we pulse-labeled cells with 5-bromo-2′-deoxyuridine (BrdU), treated them with camptothecin, and then probed for BrdU incorporation under native conditions where BrdU is detected in ssDNA but not dsDNA. Flow-cytometry-based quantification established that USP4 depletion (Figure 2E), but not SART3 depletion (Figure S2I), reduced native BrdU staining intensities in replicating cells without USP4 or SART3 depletion affecting protein levels of key resection factors (Figure S2J). Furthermore, the resection defect caused by depleting

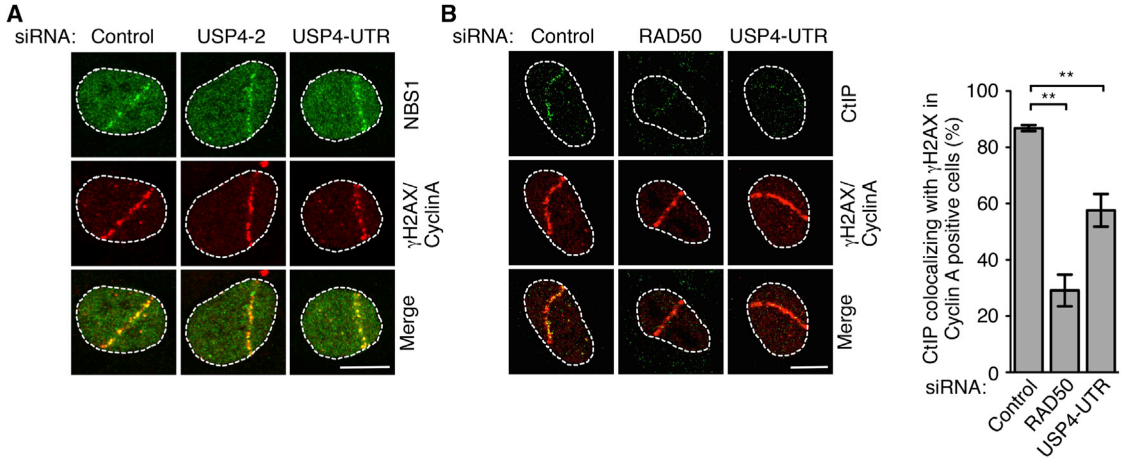

Figure 3. USP4 Regulates CtIP Recruitment to DNA Damage Sites

(A) NBS1 recruitment to laser-line micro-irradiation induced DNA lesions in Cyclin A-positive U2OS cells that were treated with USP4-targeting siRNAs was not reduced compared to the siRNA control. Nuclei were outlined, and the scale bar indicated 10 μm.

(B) CtIP recruitment to laser-line micro-irradiation induced DNA lesions in Cyclin A-positive U2OS cells was reduced after USP4 siRNA treatments. RAD50 siRNA treatment (see Figure S3A for RAD50 depletion) was the positive control (mean ± SEM; n = 3; **p < 0.01). Nuclei were outlined, and the scale bar indicated 10 μm. See also Figure S3.

endogenous USP4 was largely restored by GFP-USP4 WT(L) expression (Figure 2F). Taken together, these data indicated that, in a SART3-independent manner, USP4 promotes resection, thus at least in part explaining its impact on HR.

USP4 Regulates CtIP Recruitment to DNA Damage Sites

Because the MRN complex and CtIP play key roles in DSB resection (Sartori et al., 2007), we assessed whether their recruitment to DNA damage sites was affected by USP4. We observed that USP4 depletion did not detectably affect NBS1 (Figure 3A) or MRE11 (data not shown) recruitment to DNA damage induced by laser micro-irradiation. By contrast, CtIP recruitment to DNA damage sites in γH2AX and Cyclin-A-positive cells was significantly reduced upon USP4 depletion (Figure 3B; CtIP recruitment was also impaired in RAD50-depleted cells; see Figure S3 for RAD50 depletion). These data thus implied that USP4 promotes HR by affecting the recruitment and/or association dynamics of CtIP at DNA damage sites.

USP4 Interacts with CtIP and MRN via Its C-Terminal Insert Region

In light of the above findings, we tested whether USP4 might physically interact with CtIP and/or MRN. Indeed, when we immunoprecipitated endogenous USP4 from human 293FT cell extracts ensuing western blotting analyses readily detected both RAD50 and MRE11 (Figure 4A). Despite it being well established that CtIP interacts with MRN (e.g., Sartori et al., 2007; Wang et al., 2013), we did not detect CtIP in our immunoprecipitates. In line with our speculation that this might reflect CtIP interactions being disrupted/weakened by the anti-USP4 antibody, CtIP was detected together with RAD50 and MRE11 in immunoprecipitates generated by using an anti-GFP antibody and lysates from cells transiently expressing GFP-FLAG-fused full-length (FL) USP4 (GFP-FLAG-USP4-FL; Figure 4B; GFP-FLAG only expression was used as control. As shown in Figure S4A, these interactions were not discernibly affected by DNA damage induction). Such interactions were also seen in reciprocal studies where GFP-CtIP or GFP-FLAG-MRE11 was immunoprecipitated and ensuing samples probed for endogenous USP4 (Figures 4C and 4D, respectively).

To identify the region(s) of USP4 mediating its MRN/CtIP interactions, we expressed various USP4 deletion mutants in cells (Figure 4E; Table S3), immunoprecipitated them, and then probed for CtIP, RAD50, and MRE11 binding by western blotting. This established that interactions with CtIP and MRN were not diminished by deleting the USP4 ubiquitin-like domain 2 (UBL2) region (ΔUBL2) or the N-terminal ~30% of USP4 (ΔN). Furthermore, CtIP and MRN did not detectably interact with the UBL2 or N-terminal domain of USP4 (Figures 4E and S4B; data not shown). These results thus indicated that USP4 interactions with CtIP and the MRN complex likely occurred via the USP4 C-terminal catalytic region D1 and D2 domains and/or the C-terminal insert region (I) positioned between these domains (Figure 4E). Focusing on the USP4 catalytic domain, which structurally resembles an open right hand comprising three regions named the "thumb" (T), "fingers" (F), and "palm" (P) catalytic sub-domains (Clerici et al., 2014), we found that the USP4 "fingers" domain including the insert (F+I), but not the other regions tested, was sufficient to mediate interactions with CtIP, MRE11, and RAD50 (Figures 4E and 4F; see Figure S4C for corresponding inputs). Further analyses indicated that the USP4 insert (I), but not the fingers (F), region was sufficient for these interactions (Figures 4E and 4G; see Figure S4D for inputs), although we note that additional USP4 regions might also contribute to interactor binding.

USP4 Counteracts Its Own Ubiquitylation

During the course of our studies, we observed that mutating the USP4 catalytic cysteine to alanine (C311A) to render USP4

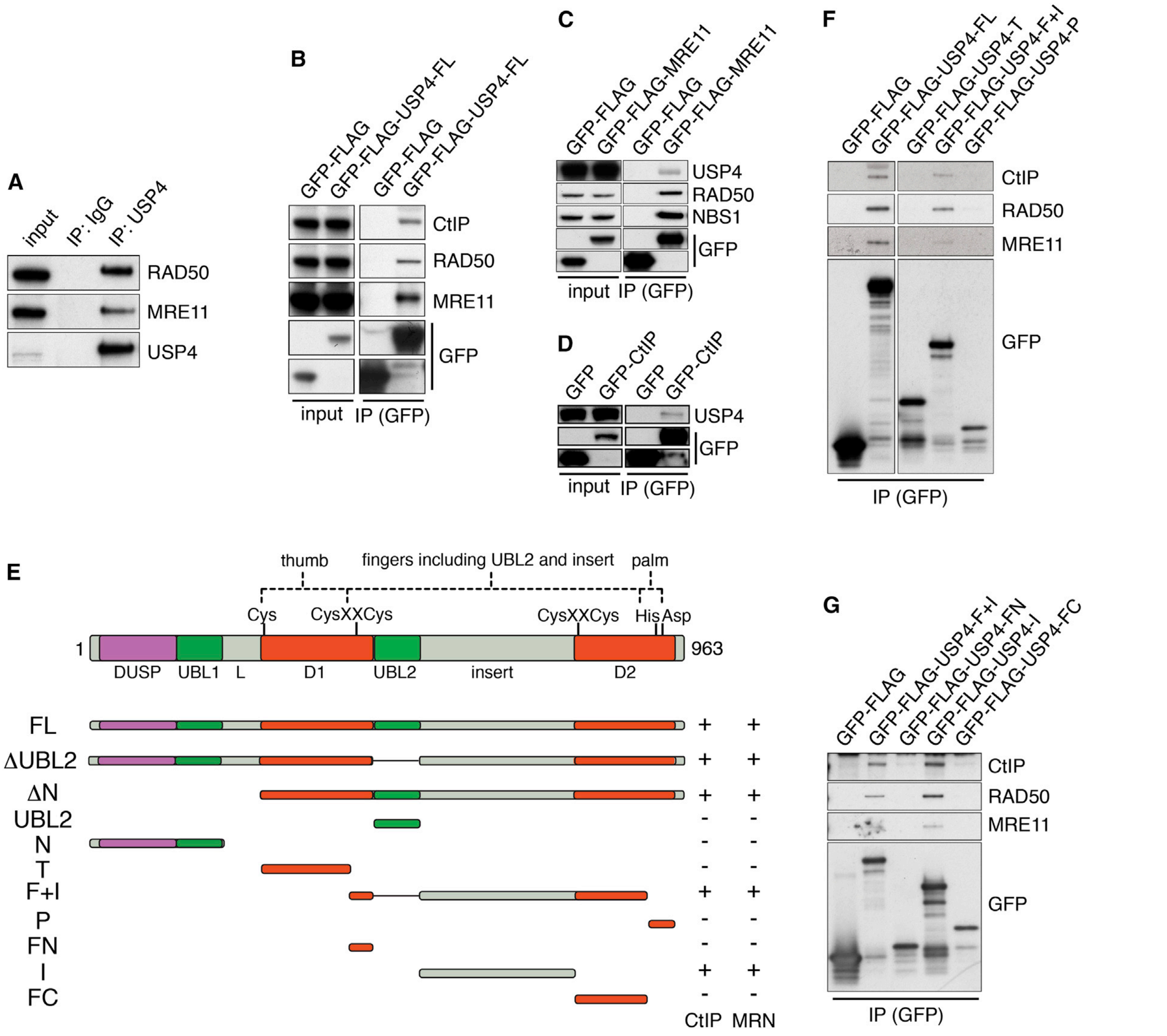

Figure 4. USP4 Interacts with CtIP and MRN via Its C-Terminal Insert Region

(A) Endogenous USP4 immunoprecipitation (IP) from 293FT cell extracts retrieved RAD50 and MRE11.

(B) Full-length (FL) GFP-FLAG-USP4 IP from U2OS cell lysates retrieved CtIP, RAD50, and MRE11.

(C and D) (C) GFP-FLAG-MRE11 or (D) GFP-CtIP immunoprecipitations from 293FT cell extracts retrieved USP4.

(E) Schematic view of full-length (FL) USP4 with indicated structural domains. USP4 deletion mutants and their ability to retrieve CtIP or MRN were indicated. Positions of cysteine, histidine and aspartic-acid that form the USP4 catalytic triad; the zinc-binding motif cysteine residues (CysXXCys); and the "thumb," "fingers," and "palm" catalytic subdomains were indicated.

(F) GFP-FLAG-USP4-F+I immunoprecipitations retrieved CtIP, RAD50, and MRE11 (See Figure S4C for corresponding inputs; all samples were run on the same SDS-poly acrylamide gel).

(G) GFP-FLAG-USP4-I immunoprecipitations retrieved CtIP, RAD50, and MRE11 (See Figure S4D for inputs). See also Figure S4.

enzymatically inactive ("catalytic-dead" [CD]), almost totally abrogated its interactions with CtIP and MRN (Figure 5A; note that binding of USP4 to CtIP and RAD50 was not abrogated by the DNA-intercalating agent ethidium bromide (EtBr), suggesting that interaction was not mediated by DNA bridging. See Figure S5A for inputs). In light of previous work indicating that USP4 can deubiquitylate itself (Wada et al., 2006), we hypothesized that USP4 catalytic inactivation could lead to its enhanced ubiquitylation, which might block its CtIP/MRN interactions. We therefore assessed ubiquitylation of GFP-USP4 WT, GFP-USP4 CD, and GFP (assessment of endogenous USP4 ubiquitylation events could more directly address the physiological nature of such modifications but was technically not feasible; data not shown) by co-expressing these with human influenza hemagglutinin-epitope-tagged ubiquitin (HA-Ub) and immunoprecipitating ubiquitylated proteins with an HA antibody in the presence of 1 M NaCl. Western blotting of ensuing samples with a GFP antibody indicated that HA-ubiquitin retrieved USP4 CD but not USP4 WT

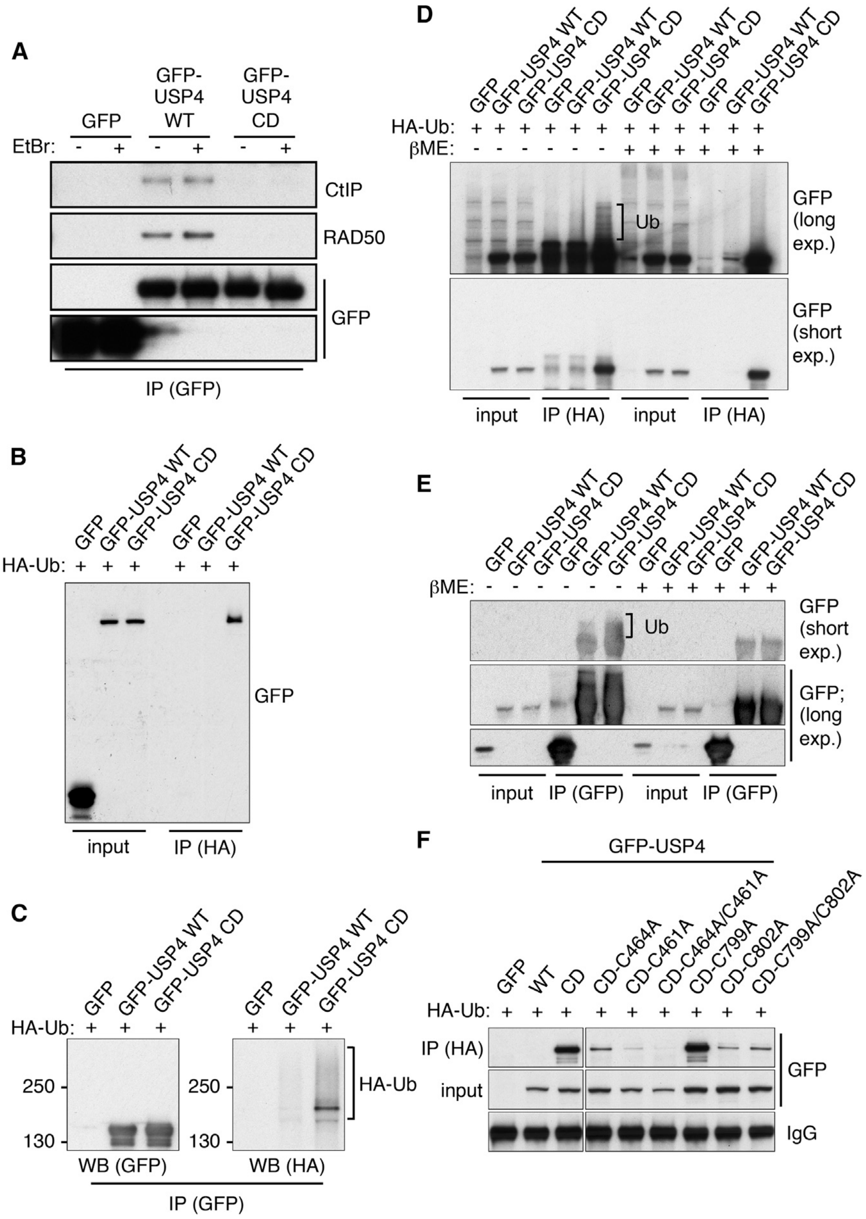

Figure 5. USP4 Counteracts Its Own Ubiquitylation

(A) GFP-USP4 WT but not CD immunoprecipitations from U2OS cell extracts retrieved CtIP and RAD50 in presence or absence of EtBr (50 μg/ml; see Figure S5A for inputs).

(B) HA-ubiquitin (HA-Ub) immunoprecipitations (in presence of 1 M NaCl) retrieved GFP-USP4 CD but no detectable GFP-USP4 WT from U2OS cell extracts.

(C) GFP-immunoprecipitations from U2OS cell extracts that expressed HA-ubiquitin and GFP, GFP-USP4 WT, or CD, followed by western blot analysis with an HA antibody retrieved HA-ubiquitylated forms of GFP-USP4 CD and to a lesser extent GFP-USP4 WT (130 or 250 indicated respective protein sizes in kDa).

(D) HA-Ub immunoprecipitations from U2OS cell extracts that were processed in absence of β-mercaptoethanol (βME) retrieved modified forms of GFP-USP4 CD that were not visible in presence of βME (exp., exposure; GFP cells were the control).

(E) GFP immunoprecipitations from U2OS cell extracts that were processed in absence of βME, retrieved modified forms of GFP-USP4 CD, and to a lesser extent GFP-USP4 WT, which were not visible in presence of βME.

(F) HA-Ub immunoprecipitations (followed by western blotting analysis) from U2OS cell extracts expressing various GFP-fused USP4 derivatives retrieved GFP-USP4 CD and CD-C799A, but less efficiently the other zinc-binding motif cysteine mutants (CD-C461A, CD-C464A, and CD-C802A; IgG, IgG heavy chain). All samples were run on the same SDS poly-acrylamide gel. See also Figure S5 and Table S4 (describes all ubiquitin sites identified by tandem mass spectrometry on USP4 WT and CD).

or GFP alone at appreciable levels (Figure 5B). Although these results might have been explained by USP4 CD displaying enhanced non-covalent binding to ubiquitin than wild-type USP4, when we carried out binding studies with recombinant ubiquitin, this was not the case (Figure S5B). To verify that USP4 was indeed ubiquitylated, we prepared GFP-immunoprecipitates from lysates of cells co-expressing HA-ubiquitin together with GFP-USP4 WT or CD. Probing ensuing western blots with an HA antibody detected smears of slower migrating products, thus identifying these as ubiquitylated USP4 derivatives (Figure 5C). Furthermore, in line with our other findings and previously reported USP4 ubiquitylation events (Wada et al., 2006), these ubiquitylated species were more prominent with GFP-USP4 CD than with GFP-USP4 WT (Figure 5C). Together with the stringency of the immunoprecipitation conditions we used, these data indicated that USP4 is ubiquitylated and that catalytically dead USP4 contained greater levels of covalently bound ubiquitin than the wild-type protein.

To assess USP4 ubiquitylation further, we carried out tandem mass spectrometry studies on GFP-USP4 CD or WT, purified from cell lysates via GFP-immunoprecipitations, followed by post-translational modification analysis to identify GlyGly modifications on amino acid residues, representing remains of ubiquitin or ubiquitin-like modifications after trypsin digestion. Thus, we detected such modifications on a considerable number of lysine residues and also on serine and threonine residues (see Table S4). Notably, our analysis also suggested ubiquitylations on non-conventional cysteine residues of both USP4 CD and WT. In particular, we noted evidence for modifications on Cys-461 or Cys-464 and Cys-799 or Cys-802 (Figures S5C and S5D; data not shown; as each pair of cysteine residues was on the same trypic peptide, it was not possible to differentiate between them), which form a flexible zinc-binding region that stabilizes the catalytic domain of USP4 (Clerici et al., 2014). Further

analysis and quantification of these ubiquitylations by mass spectrometry was not possible however, due to technical issues relating to the labile nature of ubiquitin-thioester linkages (see below) and confounding modifications of these and other USP4 cysteine residues by chemical reagents used in sample preparations (see Supplemental Information for further details).

Previous reports have proposed the existence of ubiquitin adducts in which the ubiquitin C terminus is covalently attached to target protein serine or threonine residues by ester linkages or cysteine residues by thioester linkages (e.g., Cadwell and Coscoy, 2005). While cysteine ubiquitylation has so far been largely unexplored, its prevalence may have been underestimated because the associated thioester linkage is readily disrupted by reducing agents (Huang et al., 2009) such as β-mercaptoethanol that are often used in cell extract generation and analysis. In light of this and our other data, we tested whether we could detect USP4 ubiquitylations that were sensitive to β-mercaptoethanol treatment. Thus, through HA-ubiquitin immunoprecipitations followed by western blotting in the absence or presence of β-mercaptoethanol, we identified modified forms of GFP-USP4 CD that migrated more slowly on SDS polyacrylamide gels than GFP-USP4 WT and which were lost upon β-mercaptoethanol treatment (Figure 5D). To confirm this finding, we carried out GFP immunoprecipitations from extracts containing GFP-tagged USP4 CD or USP4 WT and processed these the in absence or presence of β-mercaptoethanol. Western blot analysis with an antibody recognizing GFP revealed that USP4 CD and to a lesser extent USP4 WT were modified and that these modifications were not observed when samples had been treated with β-mercaptoethanol (Figure 5E; note that USP4 WT and CD were present in similar amounts and that it is unlikely that the observed modifications reflected differential oxidation events between wild-type and catalytically dead USP4). These results indicated that at least a fraction of ubiquitylated USP4 CD was sensitive to reducing conditions, thus supporting the conclusion that the protein is subject to cysteine ubiquitylation.

Structural data (Clerici et al., 2014) indicate that Cys-799, -802, -461, and -464 form a flexible zinc-binding region that stabilizes the USP4 catalytic domain and is exposed outward from the protein core, potentially making the region accessible for modification. While exploring the possible functional impact of these cysteine residues, we found that USP4 CD derivatives containing cysteine to alanine mutations on Cys-461 (C461A), Cys-464 (C464A), and Cys-802 (C802A) but not Cys-799 (C799A) upon co-expression in cells with HA-ubiquitin, were less readily retrieved by HA-ubiquitin immunoprecipitations from cell extracts than the GFP-USP4 CD protein itself (Figure 5F). While these findings provided support for the zinc-binding motif cysteine residues being ubiquitylated, it is also possible that mutating these sites altered USP4 structurally in a manner that reduced its overall ubiquitylation levels, perhaps by making it a less effective target for relevant E3 ubiquitin ligases. Nevertheless, as shown in Figure S5E, we found that although binding of an HA-ubiquitin activity probe was lower for USP4 WT-C464A than for USP4 WT, the Cys-464 mutation still maintained catalytic activity, implying that the USP4 structure was still at least in part intact. Together, these findings supported a model in which USP4 ubiquitylations, including those on zinc-binding cysteine residues, are subject to turnover by USP4 catalytic activity.

Ubiquitylation Counteracts USP4 Interactions and Function

Our data highlighted how disrupting USP4 catalytic activity abrogated its interactions with CtIP and MRN and also led to enhanced USP4 ubiquitylation, suggesting that these phenomena might be mechanistically linked. To address this possibility, we focused on USP4 Cys-464, whose mutation strongly reduced retrieval of USP4 CD by HA-ubiquitin immunoprecipitations. Thus, we carried out immunoprecipitation-western blot analyses to see whether introducing the Cys-464 to Ala (C464A) mutation into USP4 CD (in which the USP4 catalytic cysteine residue C311 was mutated to alanine) might restore interactions with CtIP and MRN. Indeed, while having little or no effect on CtIP/MRN interactions with GFP-USP4 WT, the C464A mutation markedly stimulated interactions between GFP-USP4 CD and CtIP/MRN (Figure 6A; see Figure S6 for corresponding inputs). To explore whether this compensatory effect extended to USP4 functions in the DDR, we generated U2OS cell lines stably expressing GFP-USP4 WT-C464A or GFP-USP4 CD-C464A (Figure 6B). Analyses of these and the previously described GFP-USP4 WT(H) and CD cell lines established that mutating the USP4 catalytic cysteine to alanine resulted in CtIP recruitment and resection defects that were largely alleviated by the C464A mutation (Figures 6C and 6D, respectively). These results thus supported a model in which USP4 functions primarily through mediating interactions with CtIP and the MRN complex rather than by targeting these or other factors for deubiquitylation.

As we had previously found that expression of USP4 CD functioned in a dominant-negative manner (Figure 1G and Figure S1F), we assessed whether this was abrogated by the C464A mutation. Indeed, neutral comet assays indicated that GFP-USP4 CD but not GFP-USP4 CD-C464A expression caused DSB repair defects in cells after phleomycin treatment (Figure 6E). Importantly, GFP-USP4 CD but not GFP-USP4 CD-C464A expression also caused DSB repair defects when endogenous USP4 was depleted from cells by siRNA treatment (Figure 6F), establishing that GFP-USP4 CD-C464A functions directly to promote DSB repair without endogenous USP4 contributing to the phenotype. Together, these findings provided support for USP4 interactions with CtIP/MRN being critical for its DDR functions and for a model wherein USP4 auto-deubiquitylation promotes these interactions and thereby USP4 functions in DSB repair.

Auto-Regulated Ubiquitylation of Other USP-Family DUBs

Based on the above findings, we hypothesized that, like USP4, other USP-family DUBs might be subject to ubiquitylation to regulate protein interactions in a manner counteracted by their catalytic activities. Focusing on USP15 and USP11, the two DUBs most related to USP4, we rendered them catalytically inactive by mutating their catalytic cysteine residue to alanine (USP15 C298A and USP11 C318A; see Figure S7A for sequence alignments). Strikingly, co-expression of these or wild-type versions with HA-ubiquitin followed by immunoprecipitations with

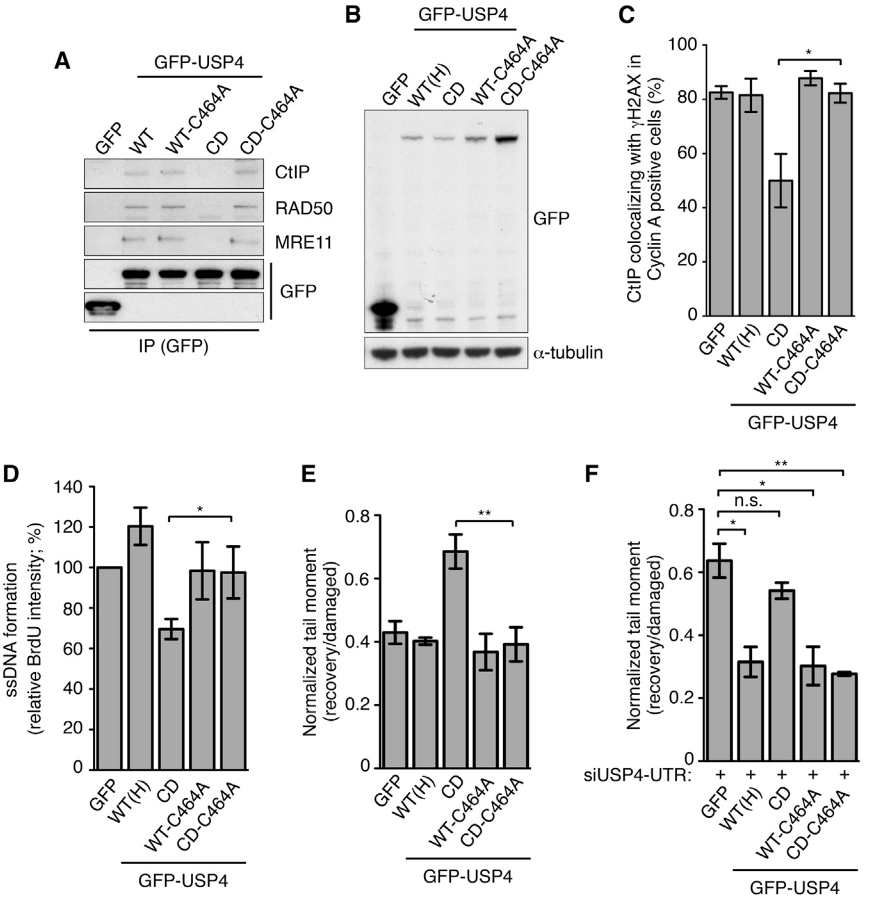

Figure 6. Ubiquitylation Counteracts USP4 Interactions and Function

(A) GFP-USP4 WT, WT-C464A, and CD-C464A but not CD immunoprecipitations retrieved CtIP, RAD50, and MRE11 (see Figure S5A for inputs).

(B) Protein levels of U2OS cells expressing GFP-USP4 WT-C464A or GFP-USP4 CD-C464A. GFP, GFP-USP4 WT (H) and CD cell lines were described previously (Figure S1C).

(C–E) Mutating USP4 Cys-464 to Ala restored (C) CtIP recruitment defects (mean ± SEM; n = 3), (D) DNA-end resection defects (mean ± SEM; n = 5), and (E) DSB repair (neutral comet assays) defects (mean ± SEM; n = 3), observed with GFP-USP4 CD-expressing U2OS cells.

(F) Mutating Cys-464 to an alanine in USP4-UTR siRNA-treated U2OS cells restored the DSB repair defects observed in GFP-USP4 CD expressing cells upon endogenous USP4 depletion (mean ± SEM; n = 3; $^{*}p < 0.05$; $^{**}p < 0.01$). See also Figure S6.

an HA antibody and western blot analysis under reducing conditions revealed that USP15 CD and USP11 CD were retrieved more strongly than their corresponding wild-type proteins (Figure 7A). Furthermore, as for USP4 CD, when samples were analyzed under non-reducing conditions, USP15 CD and USP11 CD exhibited additional slower-migrating species (Figure 7B). These findings thus suggested that, as for USP4, the catalytic activities of USP15 and USP11 counteract their respective ubiquitin modifications. To investigate USP15 further, we focused on USP15 Cys-451, which forms part of the USP15 zinc-binding motif and aligns with USP4 Cys-464, whose mutation to alanine reduced the ability of USP4 CD to be retrieved by HA-ubiquitin under stringent immunoprecipitation conditions (see Figure 5D). Notably, transient co-expression studies employing HA-ubiquitin and various USP15 derivatives followed by HA immunoprecipitation-western blotting indicated that introducing the C451A mutation reduced the amount of GFP-USP15 CD recovered with HA-ubiquitin (Figure 7C). In light of our USP4 findings, we tested whether catalytically dead USP15 was still able to interact with one of its established substrates, SMAD2/3 (Inui et al., 2011), and if this interaction was influenced by C451A mutagenesis. Thus, through immunoprecipitation-western blot analyses, we found that, unlike USP15 WT, USP15 CD was impaired in its ability to interact with SMAD2/3 and that the USP15 CD-C451A mutant restored this interaction (Figure 7D; see Figure S7B for inputs). These findings thereby supported a model in which, as for USP4, USP15 catalytic activity counteracts its own ubiquitylation to promote substrate interactions.

DISCUSSION

We have established that USP4 promotes DSB repair by HR and cellular resistance to IR and the topoisomerase I inhibitor camptothecin. Mechanistically, we found that USP4 does so at least in part by promoting DSB resection in a manner that appears to be independent of its established spliceosomal functions. Accordingly, we established that USP4-depleted cells display defects in DNA-damage-induced RPA2 phosphorylation and in RAD51 accumulation at DNA damage sites. Moreover, we found that USP4 depletion markedly impaired DNA-damage accumulation of the DNA-end resection factor CtIP. USP4 thus joins a growing number of proteins known to affect CtIP activity, highlighting the crucial importance of appropriately controlling and regulating the initiation of resection.

By assessing the properties of USP4 derivatives, we discovered that it directly or indirectly interacts with CtIP and MRN and that the USP4 insert region, which resides between the USP4 D1 and D2 catalytic subdomains, was sufficient to mediate such interactions. Moreover, we observed that inactivating USP4 catalytic function almost totally abrogated its CtIP/MRN interactions. Through exploring the mechanism of this effect, we found that catalytically inactive USP4 was retrieved more effectively by HA-tagged ubiquitin than the wild-type USP4 protein, leading us to investigate whether its CtIP/MRN interactions might be affected by USP4 auto-deubiquitylation. Indeed, by mass-spectrometry, we identified various USP4 ubiquitylations that were enhanced upon USP4 catalytic inactivation, including those on cysteine residues within an evolutionarily conserved USP4 zinc-binding motif. Consistent with the chemical nature

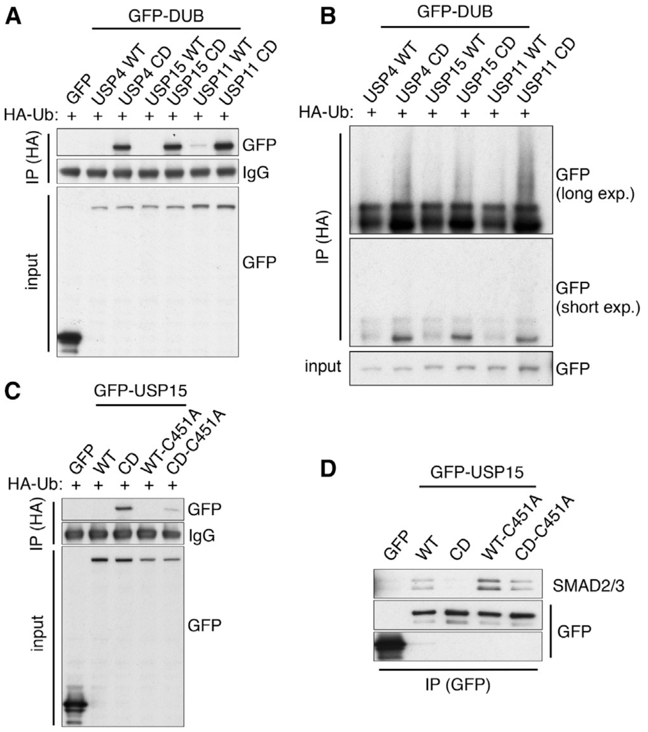

Figure 7. Auto-Regulated Ubiquitylation of Other USP-Family DUBs

(A) HA-ubiquitin (HA-Ub) immunoprecipitations (in presence of βME) retrieved USP4 CD, USP15 CD, and USP11 CD (wild-type USP4, USP15, and USP11 were not or weakly detected under these conditions).

(B) HA-Ub immunoprecipitations from U2OS cell extracts that were processed without βME retrieved modified forms of USP4 CD, USP15 CD, and USP11 CD (exp., exposure).

(C) HA-Ub immunoprecipitations from U2OS cell extracts retrieved USP15 CD-C451A less efficiently than USP15 CD.

(D) USP15 WT, WT-C451A, and CD-C451A but not CD immunoprecipitations from U2OS cell extracts (see Figure S7B for inputs) efficiently retrieved SMAD2/3. See also Figure S7.

of these ubiquitin thioester linkages, our ensuing studies highlighted their removal by β-mercaptoethanol. Moreover, mutating such cysteine residues to alanine prevented USP4 retrieval by HA-ubiquitin, restored the ability of catalytically dead USP4 to interact with CtIP/MRN, and also restored the ability of catalytically inactive USP4 to promote DSB repair, even in the absence of endogenous USP4. Taken together, our observations support a model in which USP4 is subject to ubiquitylation in a manner that interferes with CtIP and MRN binding, thus impairing resection and abrogating HR. Moreover, our results indicate that the key DDR role for USP4 catalytic function is to counteract modifications on itself, thereby promoting CtIP/MRN binding, resection, and HR. Additional biochemical analyses will be needed to address precisely how USP4 ubiquitylations inhibit its interactions with other proteins. One possibility is that ubiquitylation competes with ubiquitin that is retained by the USP4 ubiquitin-binding pocket and switching-loop motif, following substrate hydrolysis (Clerici et al., 2014; Sahtoe and Sixma, 2015). It remains to be established which ubiquitin ligase(s) mediate(s) USP4 ubiquitylation and whether the auto-regulatory paradigm we have established is constitutive or is affected by factors such as cell-cycle status, chromatin structure, or DDR signaling.

In contrast to the extensive literature on lysine ubiquitylation, few reports have described ubiquitylation of cysteine residues (e.g., Cadwell and Coscoy, 2005) other than on E1-activating enzymes, E2-conjugating enzymes, and HECT-domain E3 ubiquitin ligases (Komander, 2009a). Our mapping of USP4 cysteine ubiquitylations and our observation that such ubiquitylations are labile under reducing conditions, highlight how cysteine ubiquitylation and deubiquitylation might occur more generally, at least within the USP-DUB ubiquitin protease family, many of which contain zinc-binding cysteine motifs (Ye et al., 2009). Furthermore, we found that, as for USP4, USP15 and USP11 catalytic inactivation led to the accumulation of modified forms that were abrogated by reducing agents and that mutating Cys-451 of USP15, which aligns with USP4 Cys-464, reduced USP15 retrieval by HA-ubiquitin. Moreover, we established that, analogously to USP4, catalytically inactive USP15 was impaired in binding to its protein target, SMAD2/3, and that binding was restored by introducing the Cys-451 mutation. In light of the phylogenetic connections between USP4, USP15, and USP11, and because both USP4 (this study) and USP11 function in DNA repair (Schoenfeld et al., 2004; Wiltshire et al., 2010), it will be of interest to explore possible DDR roles for USP15. In this regard, we note that USP15 has been identified as a target for ATM-mediated phosphorylation (Mu et al., 2007) and mediates resistance to IR (Nishi et al., 2014) and that like USP4, USP11 and USP15 are recruited to sites of laser-induced DNA damage (Nishi et al., 2014). Finally, if small-molecule inhibitors of USP4, USP15, and/or USP11 are developed, it will be interesting to pursue their potential in cancer therapy.

EXPERIMENTAL PROCEDURES

For detailed descriptions of these and additional procedures, see Supplemental Experimental Procedures.

Cells, Cell Lines and Growth Conditions

U2OS cells were cultured under conventional growth conditions. All stable cells lines exogenously expressing GFP or FLAG-fused USP4 and mutant derivatives and 293FT cells were cultured in presence of 0.5 mg/ml geneticin (Life technologies). DR-GFP expressing U2OS cells were cultured in presence of 1 μg/ml puromycin.

Antibodies, SDS-PAGE, and Western Blot Analysis

See Table S2 for antibodies used in this study. SDS-PAGE and western blot analyses were performed as described previously (Nishi et al., 2014).

siRNAs, Plasmids, and Transfections

See Table S1s and S3 for respective siRNAs and plasmids described in this study. Plasmids were transfected using TransIT-LT1 (Mirus Bio) transfection reagent according to the manufacturer's instructions, and siRNA transfections (30 nM/transfection) were carried out using Hiperfect (QIAGEN) according to the manufacturer's instructions.

Neutral Comet and Clonogenic Cell Survival Assays

Neutral comet and clonogenic cell survival assays were performed as previously described (Nishi et al., 2014).

Immunoprecipitations

Immunoprecipitation experiments from U2OS or 293FT cells were performed as previously described (Blackford et al., 2015).

Live Cell Laser-Line Micro-Irradiation

GFP-USP4 WT(H)-expressing cells were BrdU sensitized and then subjected to 400 μW localized laser micro-irradiation with a 405 nm UVA laser beam (Limoli et al., 1993). Pictures were taken before and 30 min after irradiation.

DR-GFP HR Reporter Assays

HR reporter assays were performed as previously described (Nishi et al., 2014).

DNA-End Resection (BrdU) Assay

BrdU pulse-labeled U2OS cells were treated with 1 μM camptothecin, processed, and treated with BrdU and γH2AX primary and secondary antibodies (Table S2). Cells were analyzed by flow cytometry with γH2AX detection as a control for DNA damage.

Cell Cycle Analysis and Random Plasmid Integration Assays

Cell cycle analyses and random plasmid integration assays to measure NHEJ efficiencies were performed as described previously (Nishi et al., 2014).

RAD51 IRIF

Cells were treated with IR (5 Gy), allowed to recover for 8 hr, and were fixed and treated with RAD51 and γH2AX primary and secondary antibodies. γH2AX-positive cells with more than three RAD51 foci were scored.

Click-it EDU Labeling

U2OS cells were EdU pulse-labeled and fixed. EdU labeling reactions were performed using Click-iT according to the manufacturer's instructions (Thermo Fisher Scientific). EdU intensities of S-phase cell populations were measured and quantified.

NBS1 or CtIP Recruitment to Laser-Line Micro-Irradiation Induced DNA Lesions

U2OS cells were treated with BrdU (10 μM) for 24 hr, subjected to 250 μW localized laser micro-irradiation with a 405 nm UV-A laser beam, and 2 hr after irradiation were fixed and processed. Cyclin A, γH2AX, and NBS1- or CtIP-positive cells were quantified.

Mass Spectrometry and Data Analysis

Samples were analyzed using nano liquid chromatography-tandem mass spectrometry (nano-LC-MS/MS) in HCD mode as described previously (Fischer and Kessler, 2015). Raw MS data were processed and analyzed by PEAKS Version 7 (Bioinformatics Solutions) using HCD fragmentation spectra. MS/MS spectra were searched against the Swissprot (21,039 human sequence entries) database allowing for variable post-translational modifications to be applied to the de novo identified peptides. See Table S4 for identified ubiquitylations on USP4 WT and CD upon immunoprecipitation with a GFP antibody from 293FT lysates. Amino acids in bold indicate previously described USP4 ubiquitylations.

Ubiquitylation Assays

U2OS cells were co-transfected with HA-ubiquitin and GFP-tagged expression constructs. Lysates were immunoprecipitated 48 hr later with an HA antibody, and HA-retrieved proteins were subjected to western blot analysis.

Biotin-Ubiquitin Binding Assay

Streptavidin M-280 Dynabeads (Life Technologies) were soaked in an excess of Biotin-fused human recombinant ubiquitin (R&D Systems) for 1 hr at 4°C. Then, cell lysates were incubated for 16 hr at 4°C in presence of 10 μl ubiquitin-coupled streptavidin Dynabeads, after which those were washed, processed, and subjected to western blot analysis as described in the Supplemental Experimental Procedures.

Active Probe Binding Assays

Cells transfected with GFP, GFP-fused USP4 WT, CD, or WT-C464A were processed; incubated with HA-tagged ubiquitin vinyl sulfone (HA-Ub-VS) according to the manufacturer's (Enzo Life Sciences) instructions; and subjected to western blot analysis.

Statistics and Quantitative Analysis

For experiments reproduced at least three times in this study, the standard two-tailed Student's t test for statistical significance was used. For quantitative analysis, the SEM was used. All experiments were reproduced at least twice.

SUPPLEMENTAL INFORMATION

Supplemental Information includes seven figures, four tables, and Supplemental Experimental Procedures and can be found with this article online at http://dx.doi.org/10.1016/j.molcel.2015.09.019.

AUTHOR CONTRIBUTIONS

P.W. designed experiments through discussions with R.N., R.K., A.N.B., J.T., B.M.K., and S.P.J.; R.N. cloned HA-ubiquitin; J.T. carried out the RAD51 IRIF experiments; P.W. and A.N.B. prepared samples for MS analyses that were carried out by R.K. and B.M.K.; P.W. carried out all further studies; P.W. and S.P.J., with input from R.N., wrote the paper; all other authors commented and suggested revisions for the paper.

CONFLICTS OF INTEREST

S.P.J. is a founder and part-time chief scientific officer (CSO) of MISSION Therapeutics Ltd., which is developing DUB inhibitors for therapeutic applications. B.M.K. is associated with Cancer Research Technologies and Forma Therapeutics. The other authors declare no competing financial interests.

ACKNOWLEDGMENTS

We thank all members of the S.P.J. and B.M.K. laboratories and Jorrit Tjeertes for helpful discussions, and Kate Dry for commenting on the manuscript. We thank Carlos le Sage for help with FLAG-Tev-Strep tag plasmid design and helpful discussions; Matylda Sczaniecka-Clift, Muku Demir, and Julia Coates for help with stable cell line establishment; and Tobias Oelschlaegel for producing GFP-FLAG-MRE11 polyclonal U2OS cells. We also thank Matylda Sczaniecka-Clift and Nicola Geisler for purifying USP4 peptides for antibody production. Research in the S.P.J. laboratory is funded by CRUK Program Grant C6/A11224, the European Community Seventh Framework Program grant agreement no. HEALTH-F2-2010-259893 (DDResponse), and ERC Advanced Grant DDREAM. R.N. was funded by the Daiichi Sankyo Foundation of Life Sciences fellowship and the CRUK Project Grant C6/A14831. Cancer Research UK Grant C6946/A14492 and Wellcome Trust Grant WT092096 provided core infrastructure funding. S.P.J receives his salary from the University of Cambridge, supplemented by CRUK. The John Fell Fund 133/075 and the Wellcome Trust grant 097813/Z/11/Z funded research in the B.M.K. laboratory performed by R.K. and B.M.K.

Received: April 29, 2015
Revised: August 20, 2015
Accepted: September 18, 2015
Published: October 8, 2015

REFERENCES

Al-Salihi, M.A., Herhaus, L., Macartney, T., and Sapkota, G.P. (2012). USP11 augments TGFβ signalling by deubiquitylating ALK5. Open Biol. *2*, 120063.

Blackford, A.N., Nieminuszczy, J., Schwab, R.A., Galanty, Y., Jackson, S.P., and Niedzwiedz, W. (2015). TopBP1 interacts with BLM to maintain genome stability but is dispensable for preventing BLM degradation. Mol. Cell *57*, 1133–1141.

Cadwell, K., and Coscoy, L. (2005). Ubiquitination on nonlysine residues by a viral E3 ubiquitin ligase. Science *309*, 127–130.

Ciccia, A., and Elledge, S.J. (2010). The DNA damage response: making it safe to play with knives. Mol. Cell *40*, 179–204.

Clague, M.J., Heride, C., and Urbé, S. (2015). The demographics of the ubiquitin system. Trends Cell Biol. *25*, 417–426.

Clerici, M., Luna-Vargas, M.P.A., Faesen, A.C., and Sixma, T.K. (2014). The DUSP-Ubl domain of USP4 enhances its catalytic efficiency by promoting ubiquitin exchange. Nat. Commun. *5*, 5399.

Fischer, R., and Kessler, B.M. (2015). Gel-aided sample preparation (GASP)–a simplified method for gel-assisted proteomic sample generation from protein extracts and intact cells. Proteomics *15*, 1224–1229.

Gravel, S., Chapman, J.R., Magill, C., and Jackson, S.P. (2008). DNA helicases Sgs1 and BLM promote DNA double-strand break resection. Genes Dev. *22*, 2767–2772.

Hershko, A., Heller, H., Elias, S., and Ciechanover, A. (1983). Components of ubiquitin-protein ligase system. Resolution, affinity purification, and role in protein breakdown. J. Biol. Chem. *258*, 8206–8214.

Huang, D.T., Ayrault, O., Hunt, H.W., Taherbhoy, A.M., Duda, D.M., Scott, D.C., Borg, L.A., Neale, G., Murray, P.J., Roussel, M.F., and Schulman, B.A. (2009). E2-RING expansion of the NEDD8 cascade confers specificity to cullin modification. Mol. Cell *33*, 483–495.

Inui, M., Manfrin, A., Mamidi, A., Martello, G., Morsut, L., Soligo, S., Enzo, E., Moro, S., Polo, S., Dupont, S., et al. (2011). USP15 is a deubiquitylating enzyme for receptor-activated SMADs. Nat. Cell Biol. *13*, 1368–1375.

Jackson, S.P., and Bartek, J. (2009). The DNA-damage response in human biology and disease. Nature *461*, 1071–1078.

Jackson, S.P., and Durocher, D. (2013). Regulation of DNA damage responses by ubiquitin and SUMO. Mol. Cell *49*, 795–807.

Komander, D. (2009a). The emerging complexity of protein ubiquitination. Biochem. Soc. Trans. *37*, 937–953.

Komander, D., Clague, M.J., and Urbé, S. (2009b). Breaking the chains: structure and function of the deubiquitinases. Nat. Rev. Mol. Cell Biol. *10*, 550–563.

Limoli, C.L., Ward, J.F., Ward, J.F., and Ward, J.F. (1993). A new method for introducing double-strand breaks into cellular DNA. Radiat. Res. *134*, 160–169.

Mu, J.-J., Wang, Y., Luo, H., Leng, M., Zhang, J., Yang, T., Besusso, D., Jung, S.Y., and Qin, J. (2007). A proteomic analysis of ataxia telangiectasia-mutated (ATM)/ATM-Rad3-related (ATR) substrates identifies the ubiquitin-proteasome system as a regulator for DNA damage checkpoints. J. Biol. Chem. *282*, 17330–17334.

Nishi, R., Wijnhoven, P., le Sage, C., Tjeertes, J., Galanty, Y., Forment, J.V., Clague, M.J., Urbé, S., and Jackson, S.P. (2014). Systematic characterization of deubiquitylating enzymes for roles in maintaining genome integrity. Nat. Cell Biol. *16*, 1016–1026.

Polo, S.E., and Jackson, S.P. (2011). Dynamics of DNA damage response proteins at DNA breaks: a focus on protein modifications. Genes Dev. *25*, 409–433.

Polo, S.E., Blackford, A.N., Chapman, J.R., Baskcomb, L., Gravel, S., Rusch, A., Thomas, A., Blundred, R., Smith, P., Kzhyshkowska, J., et al. (2012). Regulation of DNA-end resection by hnRNPU-like proteins promotes DNA double-strand break signaling and repair. Mol. Cell *45*, 505–516.

Sahtoe, D.D., and Sixma, T.K. (2015). Layers of DUB regulation. Trends Biochem. Sci. *40*, 456–467.

Sartori, A.A., Lukas, C., Coates, J., Mistrik, M., Fu, S., Bartek, J., Baer, R., Lukas, J., and Jackson, S.P. (2007). Human CtIP promotes DNA end resection. Nature *450*, 509–514.

Schoenfeld, A.R., Apgar, S., Dolios, G., Wang, R., and Aaronson, S.A. (2004). BRCA2 is ubiquitinated in vivo and interacts with USP11, a deubiquitinating enzyme that exhibits prosurvival function in the cellular response to DNA damage. Mol. Cell. Biol. *24*, 7444–7455.

Song, E.J., Werner, S.L., Neubauer, J., Stegmeier, F., Aspden, J., Rio, D., Harper, J.W., Elledge, S.J., Kirschner, M.W., and Rape, M. (2010). The Prp19 complex and the Usp4Sart3 deubiquitinating enzyme control reversible ubiquitination at the spliceosome. Genes Dev. *24*, 1434–1447.

Stucki, M., Clapperton, J.A., Mohammad, D., Yaffe, M.B., Smerdon, S.J., and Jackson, S.P. (2005). MDC1 directly binds phosphorylated histone H2AX to regulate cellular responses to DNA double-strand breaks. Cell *123*, 1213–1226.

Wada, K., Tanji, K., and Kamitani, T. (2006). Oncogenic protein UnpEL/Usp4 deubiquitinates Ro52 by its isopeptidase activity. Biochem. Biophys. Res. Commun. *339*, 731–736.

Wang, H., Shi, L.Z., Wong, C.C.L., Han, X., Hwang, P.Y.-H., Truong, L.N., Zhu, Q., Shao, Z., Chen, D.J., Berns, M.W., et al. (2013). The interaction of CtIP and Nbs1 connects CDK and ATM to regulate HR-mediated double-strand break repair. PLoS Genet. *9*, e1003277.

Wiltshire, T.D., Lovejoy, C.A., Wang, T., Xia, F., O'Connor, M.J., and Cortez, D. (2010). Sensitivity to poly(ADP-ribose) polymerase (PARP) inhibition identifies ubiquitin-specific peptidase 11 (USP11) as a regulator of DNA double-strand break repair. J. Biol. Chem. *285*, 14565–14571.

Ye, Y., Scheel, H., Hofmann, K., and Komander, D. (2009). Dissection of USP catalytic domains reveals five common insertion points. Mol. Biosyst. *5*, 1797–1808.

Zhang, X., Berger, F.G., Yang, J., and Lu, X. (2011). USP4 inhibits p53 through deubiquitinating and stabilizing ARF-BP1. EMBO J. *30*, 2177–2189.

Zhang, L., Zhou, F., Drabsch, Y., Gao, R., Snaar-Jagalska, B.E., Mickanin, C., Huang, H., Sheppard, K.-A., Porter, J.A., Lu, C.X., and ten Dijke, P. (2012). USP4 is regulated by AKT phosphorylation and directly deubiquitylates TGF-β type I receptor. Nat. Cell Biol. *14*, 717–726.

Molecular Cell, Volume *60*

Supplemental Information

USP4 Auto-Deubiquitylation

Promotes Homologous Recombination

Paul Wijnhoven, Rebecca Konietzny, Andrew N. Blackford, Jonathan Travers, Benedikt M. Kessler, Ryotaro Nishi, and Stephen P. Jackson

Figure S1 (Related to Figure 1)

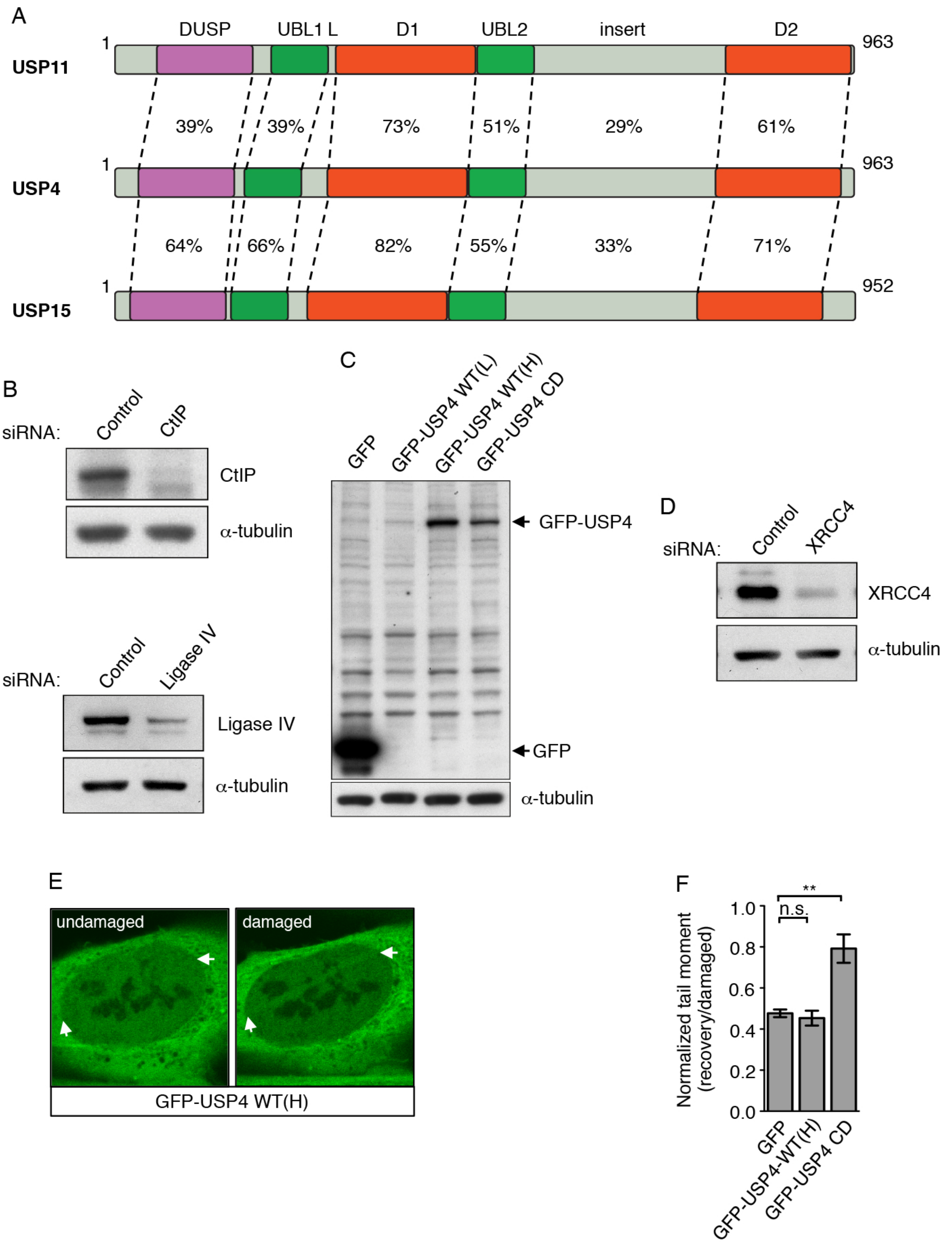

Figure S1 (Related to Figure 1)

(A) Graphic representation of USP4 and its paralogs USP15 and USP11. Domain structures and corresponding degrees of identity (%) to USP4 are indicated. DUSP = domain in USP, UBL1 = ubiquitin-like 1, L = linker region, D1 = catalytic subdomain 1, UBL2 = ubiquitin-like 2, insert = C-terminal insert, D2 = catalytic subdomain 2. (B) Treatments with CtIP or DNA Ligase IV siRNAs depleted CtIP or DNA Ligase IV from U2OS cells, respectively. Luciferase (Control) siRNA treatment was the control. (C) GFP, GFP-USP4 WT(L; where L = low expressing), GFP-USP4 WT(H; where H = high expressing) or GFP-USP4 CD protein levels from established monoclonal U2OS cell lines. (D) Treatment with XRCC4 siRNAs depleted XRCC4 in U2OS cells. Luciferase (Control) siRNA treatment was the control. (E) GFP-USP4 WT(H) stably expressed in BrdU treated (10 μM for 24 hours) U2OS cells accumulated to laser-line micro-irradiation induced DNA damage sites. (F) Exogenously expressed GFP-USP4 WT(H), but not CD rescued DSB repair defects after phleomycin treatment (neutral comet assays; mean ± s.e.m., n=3, **P < 0.01, n.s. = not significant).

Figure S2 (Related to Figure 2)

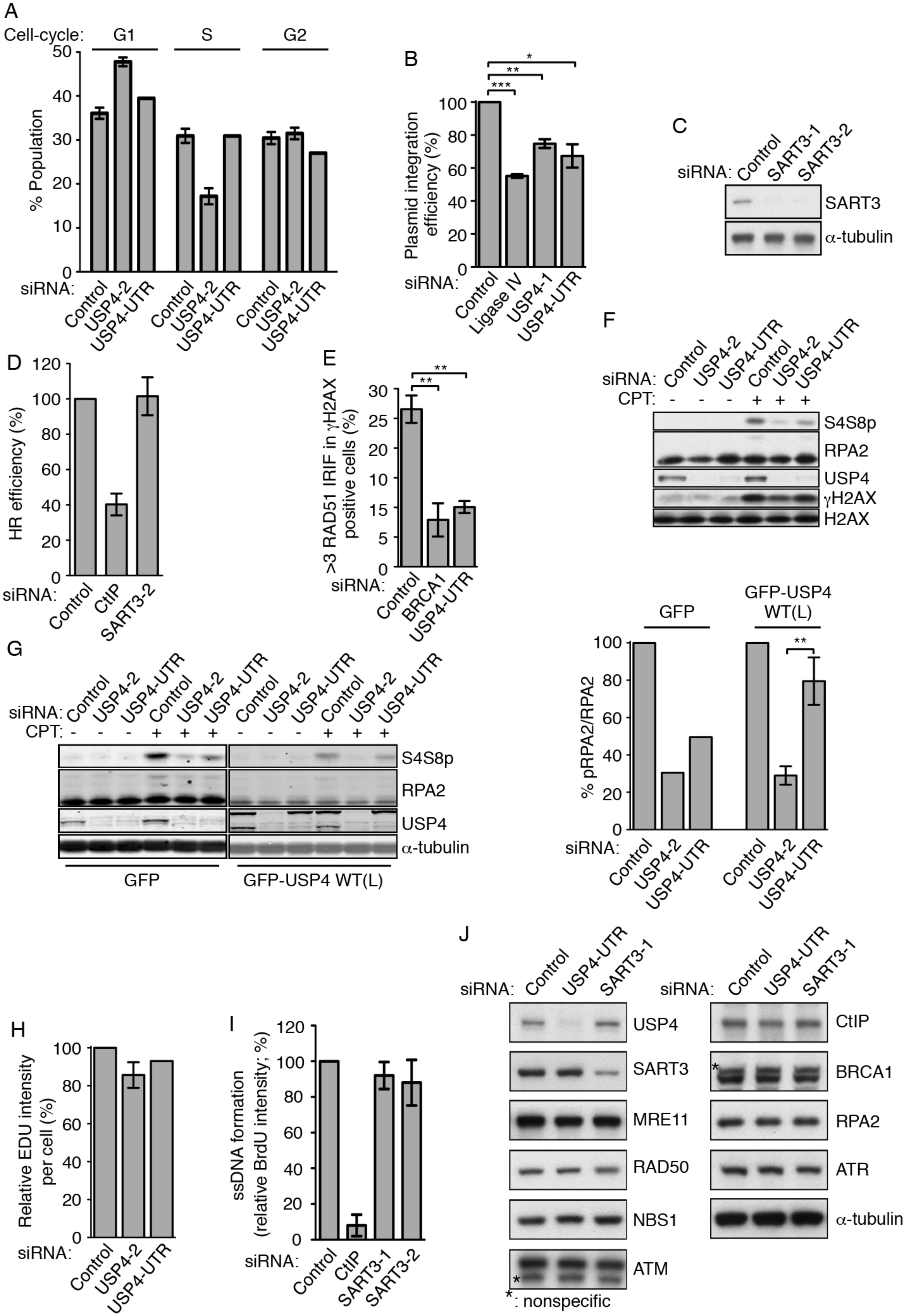

Figure S2 (Related to Figure 2)

(A) Cell cycle profiles of U2OS cells treated with luciferase (Control; n=3), USP4 [USP4-2; n=3 (mean ± s.e.m.) or USP4-UTR; n=2] siRNAs and pulse-labelled with BrdU (10 μM for 30 minutes). (B) USP4 depletion caused NHEJ defects: Random plasmid integration assays with luciferase (Control), Ligase IV or USP4 (USP4-1 or USP4-UTR) siRNA treated U2OS cells. Samples were normalized to Control siRNA treatments and set to 100% (mean ± s.e.m., n=3). (C) Treatment with SART3 siRNAs depleted SART3 from U2OS cells. Luciferase (Control) siRNA treatment was the control. (D) SART3 depletion did not cause HR defects [direct-repeat (DR)-GFP reporter assays]. Quantifications were normalized to luciferase (Control) siRNA treatments, and set to 100%. siCtIP was the positive control (mean ± s.e.m., n=4). (E) siUSP4 treatment reduced RAD51 loading after IR exposure. RAD51 ionizing irradiation-induced foci (IRIF) after luciferase (Control), BRCA1 or USP4 (USP4-UTR) siRNA treatment of U2OS cells. Cells were subsequently exposed to IR (5 Gy) and recovered for eight hours under normal growth conditions. Cells exhibiting more than three RAD51 foci in γH2AX positive cells were scored positive (mean ± s.e.m., n=3). (F) USP4 depletion reduced RPA2 Ser-4/Ser-8 phosphorylation (S4S8p) after camptothecin treatment. Luciferase (Control) or USP4 (USP4-2 or USP4-UTR) siRNA treated U2OS cells were exposed to 1 μM camptothecin for one hour. Samples were taken before and after camptothecin treatment and analyzed by western blotting with the indicated antibodies (See Figure 2D for RPA S4S8p quantifications). (G) Complementation of U2OS cells with GFP-USP4 WT(L) restored the RPA2 S4S8 phosphorylation defects observed upon siUSP4 treatment of GFP expressing cells. Representative western blots and RPA2 S4S8 phosphorylation quantifications of U2OS cells stably expressing GFP or GFP-USP4 WT(L), treated with luciferase (Control) or USP4 (USP4-2 or USP4-UTR) siRNAs and camptothecin under conditions described above [western blots describing GFP or GFP-USP4 WT(L) results were independently produced]. Per cell line, camptothecin treated GFP or GFP-USP4 WT(L) cells were set to 100% (mean ± s.e.m., GFP; n=2, GFP-USP4 WT(L); n=4). (H) USP4 depletion did not notably affect EdU incorporation during replication. U2OS cells were treated with the indicted siRNAs and pulse labelled with EdU (10 μM for 15 minutes). EdU signal intensities of S-phase cells were measured after EdU labelling (Larrieu et al., 2014) was performed and samples were normalized to Luciferase (Control) siRNA treatments, which was set to 100%. For Control and USP4-2 treatments the mean values were calculated from biological triplicates (n=3, mean ± s.e.m.) and duplicates (n=2) for USP4-UTR treatments. (I) SART3 siRNA treatment, followed by camptothecin (1 μM, 1 h) exposure of U2OS cells did not significantly reduce resection (BrdU intensities) compared to Control (luciferase) siRNA treatment. Quantifications were normalized to the camptothecin treated Control siRNA treatments (CtIP depletion was the positive control; mean ± s.e.m., n=3). (J) USP4 or SART3 siRNA treatments did not noticeably affect canonical resection factor protein levels compared to Control (luciferase) siRNA treatment (*$P < 0.05$, **$P < 0.01$, ***$P < 0.001$).

Figure S3 (Related to Figure 3)

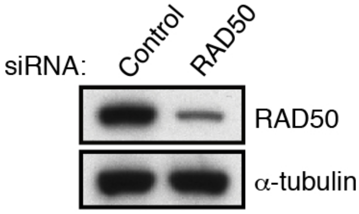

Figure S3 (Related to Figure 3)
Treatment with RAD50 siRNAs depleted RAD50 from U2OS cells. Luciferase (Control) siRNA treatment was the control.

Figure S4 (Related to Figure 4)

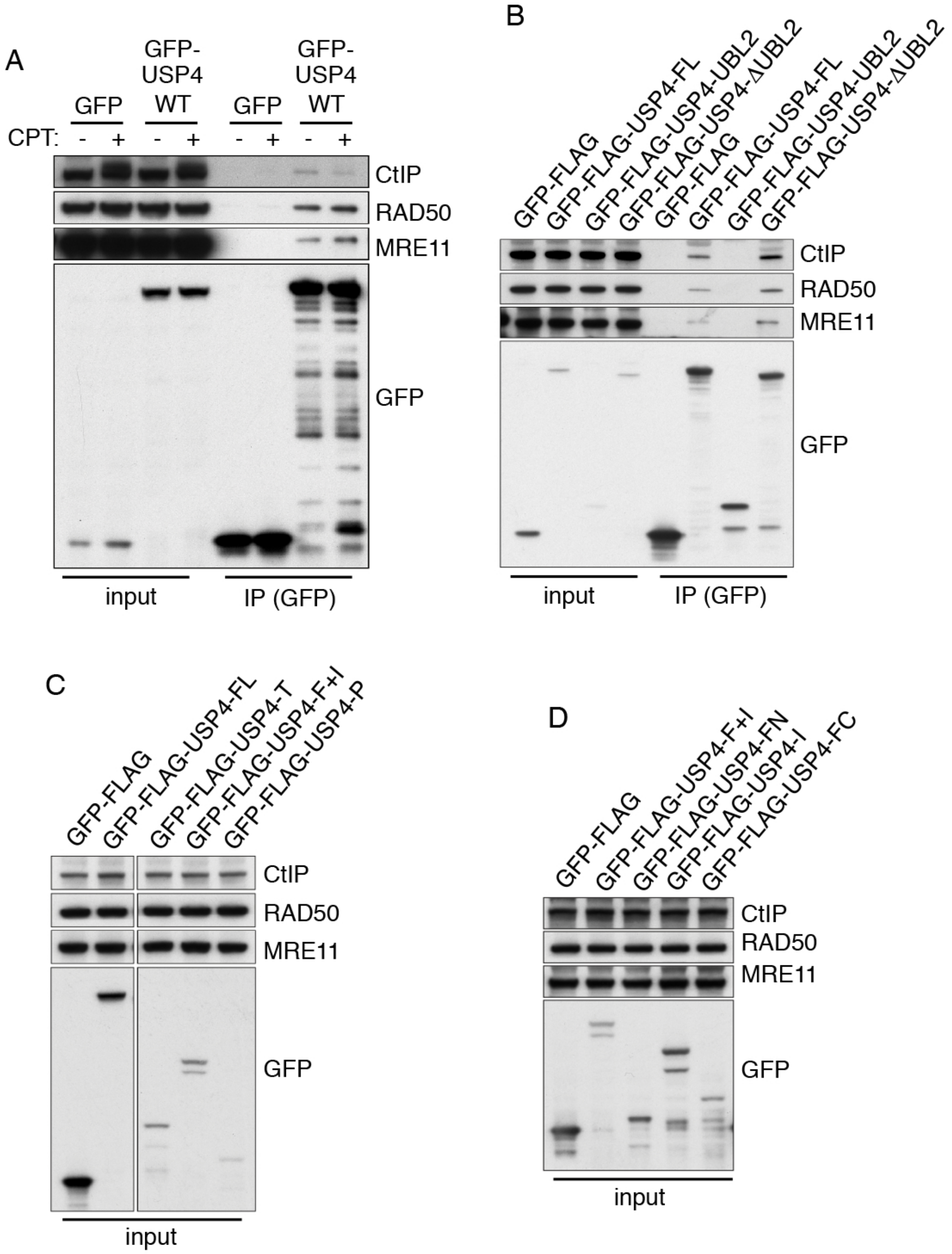

Figure S4 (Related to Figure 4)

(A) GFP-USP4 WT immunoprecipitations retrieved CtIP, RAD50 and MRE11 independent of DNA damage. GFP IP-western blot analysis with indicated antibodies, of untreated or camptothecin (CPT; 1 μM for 1 h) treated U2OS cells transiently expressing GFP or GFP-USP4 WT. (B) The USP4-UBL2 region was not necessary for the USP4-CtIP/MRN interactions. GFP immunoprecipitations and western blot analysis with the indicated antibodies, from protein lysates of U2OS cells transiently expressing GFP-FLAG, full length (FL) GFP-FLAG-USP4 or GFP-FLAG-fused USP4 mutants (see Figure 4E and Table S3 for descriptions of the USP4 truncation mutants). (C, D) Input fractions corresponding to (C) Figure 4F or (D) Figure 4G, respectively.

Figure S5 (Related to Figure 5)

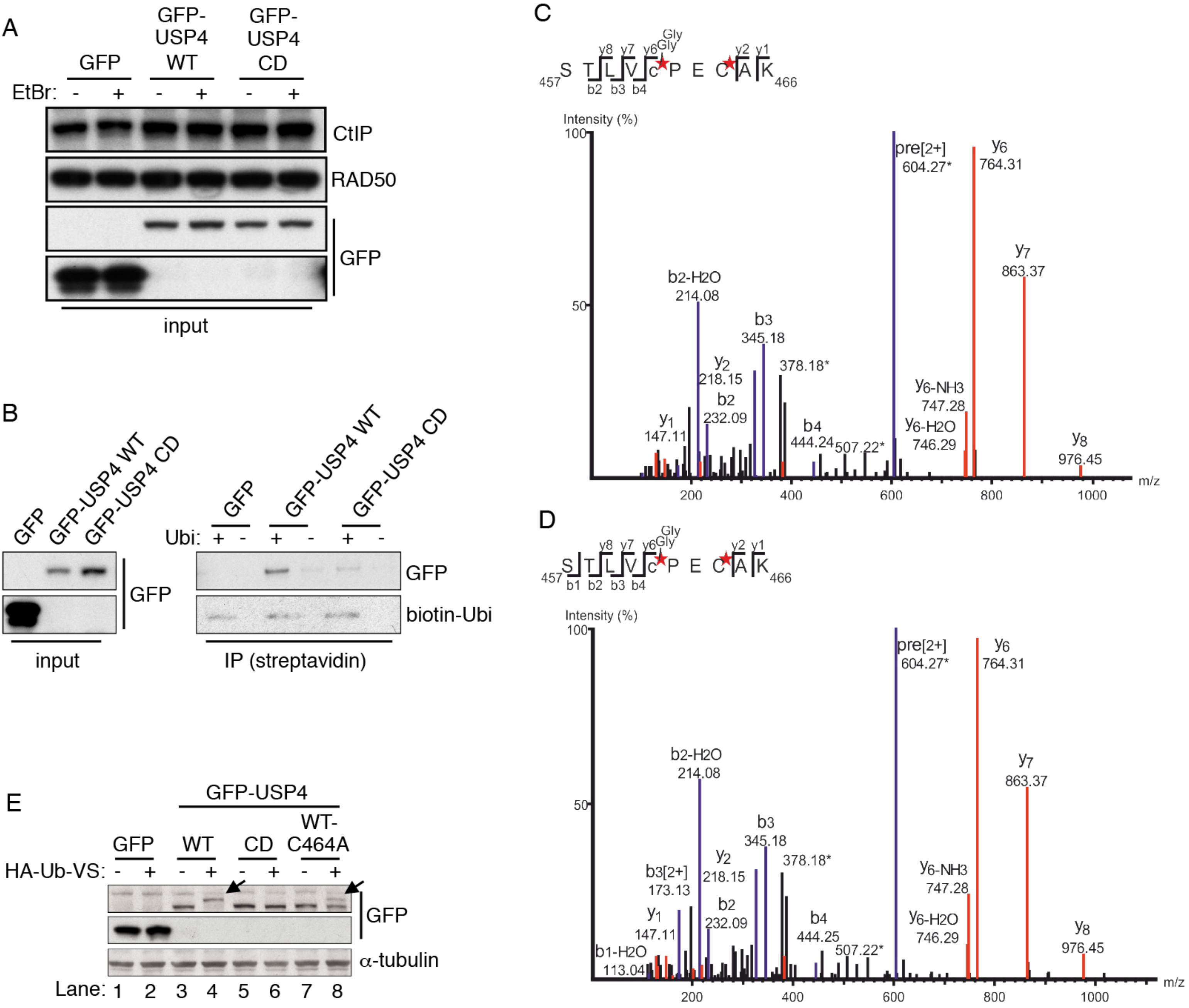

Figure S5 (Related to Figure 5)

(A) Input fractions corresponding to Figure 5A. (B) Immobilized ubiquitin retrieved GFP-USP4 WT and CD. Human amino-terminally biotinylated ubiquitin (Biotin-Ubi) was immobilized onto streptavidin Dynabeads, incubated in lysates from U2OS cells expressing GFP, GFP-USP4 WT or CD and analyzed by western blotting with the indicated antibodies. (C, D) Mass spectrometry and PEAKS analysis identified modified Cys-461 and/or Cys-464 residues suggesting possible cysteine ubiquitylations on (C) USP4 CD and (D) USP4 WT. Samples were prepared by GFP-immunoprecipitation of lysates from HEK293FT cells that transiently expressed GFP-USP4 CD or GFP-USP4 WT. The ensuing eluted material was subjected to proteolytic digestion and mass spectrometry analysis. MS/MS spectra were analyzed using PEAKS (Version 7, Bioinformatics Solutions) and indicated a match to the $_{457}$STLVCPECAK$_{466}$ peptide of USP4 (UniProt Nr Q13107). b- and y-ions are marked in blue and red, respectively, indicating a GlyGly modification either at Cys-461 or Cys-464. Asterisks indicate an alternative interpretation, assigning the y5/y4/y3 peaks as carbamidomethylation (57.02) on Cys 461 and Cys 464. Further characterization (including relative quantitation) failed, because when the carbamidomethylation step was omitted, the digestion performed with or without reduction by dithiothreitol (DTT), or when iodoacetamide was replaced by alternative alkylation reagents such as N-ethylmaleimide, this did not lead to the detection of the unmodified peptide counterpart, and further modification assignments were inconclusive. (E) USP4 Cys-464 to alanine mutation decreased but did not abolish the USP4 enzymatic activity. HA-ubiquitin probe binding assay and subsequent western blot analysis with the indicated antibodies of U2OS cells transiently expressing GFP, GFP-USP4 WT, CD or WT-C464A.

Figure S6 (Related to Figure 6)

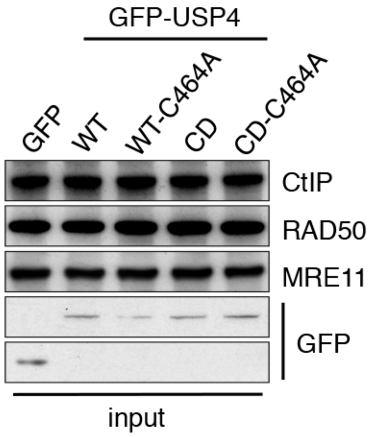

Figure S6 (Related to Figure 6)
Input fractions that correspond to Figure 6A.

Figure S7 (Related to Figure 7)

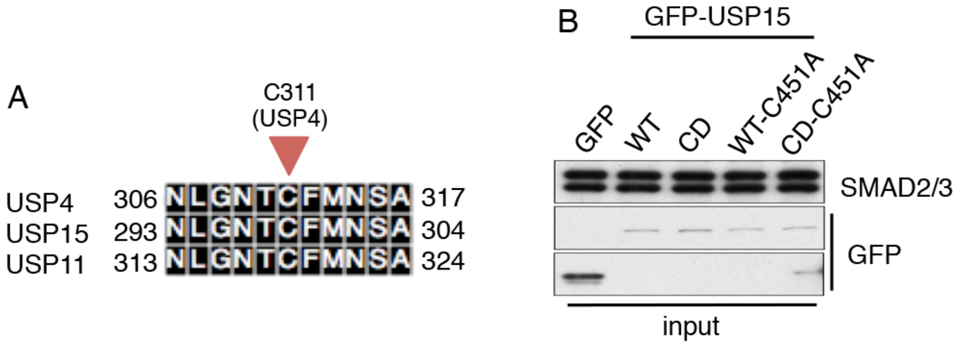

Figure S7 (Related to Figure 7)
(A) Multiple sequence alignments showing the evolutionary conserved catalytic cysteine residues of USP4, USP15 and USP11. (B) Input fractions corresponding to Figure 7D.

Supplemental Tables

Table S1 (Related to Figures 1-3 and 6): siRNAs Used in This Study

siRNA	Sequence	Supplier
siControl (luciferase)	5’ AACGUACGCGGAAUACUUCGA ‘3	Eurofins
siUSP4-1	5’ CAGGCAGACCTTGCAGTCAAA ‘3	Eurofins
siUSP4-2	5’ CACCTACGAGCAGTTGAGCAA ‘3	Eurofins
siUSP4-3	5’ ACCGAGGCGTGGAATAAACTA ‘3	Qiagen
siUSP4-4	5’ TAGATGAATTAAGACGGTTAA ‘3	Qiagen
siUSP4-UTR	5’ UUAAACAGGUGGUGAGAAA ‘3	Eurofins
siCtIP	5’ GCUAAAACAGGAACGAAUC ‘3	Eurofins
siLigase IV	5’ AGGAAGUAUUCUCAGGAAUUA ‘3	Eurofins
siXRCC4	5’ AUAUGUUGGUGAACUGAGA ‘3	Eurofins
siBRCA1	5’ GGAACCUGUCUCCACAAG ‘3	Eurofins
siRAD50	5’ CUGCGACUUGCUCCAGAUAAA ‘3	Eurofins
siSART3-1	5’ GGAGACAGGAAAUGCCUUA ‘3	Eurofins
siSART3-2	5’ GAUGUGGUGUCCUGAGAUA ‘3	Eurofins

[a]USP4 siRNA pool (siUSP4-pool; see Figure 1A and 1B) consisted of pooled USPs 4-1, 4-2, 4-3 and 4-4 siRNAs.

Table S2 (Related to Figures 1-7): Antibodies Used in This Study

Antibody target	Supplier	Catalogue no.	Clone no.	Application	Dilution
USP4	Bethyl	A300-830A		WB	1,000
α-tubulin	Sigma	T9026		WB	1,000
Flag	Sigma	F7425		WB	1,000
GFP	Roche	118144600001		WB	1,000
CtIP	(Yu and Baer, 2000)			WB/IF	100/7.5
Ligase IV	(Riballo et al., 1999)			WB	3,000
XRCC4	Abcam	ab145		WB	500
SART3	Bethyl	A301-521A		WB	1,000
γH2AX	Millipore	05-636		WB/IF	1,000/100
RAD51	Santa Cruz	sc-8349	H-92	IF	100
RPA2 (pS4/pS8)	Bethyl	A300-245A		WB	10,000
γH2AX	Cell Signaling	2577		IF	100
RPA2	Abcam	ab2175		WB	1,000
H2AX	Abcam	ab11175		WB	5,000
BrdU	Amersham	RPN20AB		IF	200
MRE11	Genetex	GTX70212/ab214		WB	1,000
RAD50	Genetex	GTX70228/ab89		WB	1,000
NBS1	NovusBio	NB100-143		IF	800
ATM	Abcam	ab32420		WB	1000
BRCA1	Santa Cruz	sc-642		WB	500
ATR	Santa Cruz	sc-1887	N-19	WB	200
USP4	Bethyl	A300-829A		IP	1 μl/mg
USP4	Santa Cruz	sc-376000	H-3	WB	250
Cyclin A	Santa Cruz	sc-751	H432	IF	500
Cyclin A	BD	611268		IF	200
HA	CRUK	12CA5	12CA5	WB	2,000
SMAD2/3	Cell Signaling	8685	D7G7	WB	1,000
Goat anti-rabbit IgG HRP	Fisher	31462		WB	5,000
Goat anti-rabbit IgG HRP	Dako	P0260		WB	5,000
AF 594 (Goat anti-rabbit IgG)	Probes	A11037		IF	1,000
AF 488 (goat anti-rabbit IgG)	Probes	A11034		IF	1,000
AF 594 (Goat anti-mouse IgG)	Probes	A11032		IF	1,000
AF 488 (Goat anti-mouse IgG)	Probes	A11029		IF	1,000
IRDye 680CW Donkey anti (M)	Licor	926-32222		WB	25,000
IRDye 800CW Donkey anti (R)	Licor	926-32213		WB	25,000

[a]Abbreviations: WB = western blotting, IF = immunofluorescence, IP = immunoprecipitation, AF = Alexa-Fluor, M = mouse, R = rabbit.

Table S3 (Related to Figures 1, 2 and 4-7): Plasmids Used in This Study

Plasmid	Gene	Amino-acid(s)	Alteration
GFP	-	-	-
GFP-USP4 WT	USP4	-	-
GFP-USP4 CD	USP4	Cys-311	Ala
FLAG-Tev-Strep	-	-	-
FLAG-(Tev-Strep)-USP4 WT	USP4	-	-
GFP-FLAG-MRE11	MRE11	-	-
GFP-CtIP	CtIP	-	-
GFP-FLAG	-	-	-
GFP-FLAG-USP4-FL	USP4	-	-
GFP-FLAG-USP4-ΔUBL2	USP4	484-571	deleted
GFP-FLAG-USP4-UBL2	USP4	484-571	expressed
GFP-FLAG-USP4-ΔN	USP4	1-307	deleted
GFP-FLAG-USP4-N	USP4	226-963	deleted
GFP-FLAG-USP4-T	USP4	308-444	expressed
GFP-FLAG-USP4-F+I	USP4	444-862/484-571	expressed/deleted
GFP-FLAG-USP4-P	USP4	863-927	expressed
GFP-FLAG-USP4-FC	USP4	444-483	expressed
GFP-FLAG-USP4-I	USP4	572-773	expressed
GFP-FLAG-USP4-FN	USP4	774-862	expressed
HA-Ub	ubiquitin	-	-
GFP-USP4 WT-C464A	USP4	Cys-464	Ala
GFP-USP4 CD-C464A	USP4	Cys-311/Cys-464	Ala/Ala
GFP-USP4 CD-C461A	USP4	Cys-311/Cys-461	Ala/Ala
GFP-USP4 CD-C799A	USP4	Cys-311/Cys-799	Ala/Ala
GFP-USP4 CD-C802A	USP4	Cys-311/Cys-802	Ala/Ala
GFP-USP15 WT	USP15	-	-
GFP-USP15 CD	USP15	Cys-298	Ala
GFP-USP15 WT-C451A	USP15	Cys-451	Ala
GFP-USP15 CD-C451A	USP15	Cys-298/Cys-451	Ala/Ala
GFP-USP11 WT	-	-	-
GFP-USP11 CD	USP11	Cys-318	Ala

[a]Regions with the corresponding alterations are indicated in column three and four, respectively.

[b]Abbreviations: GFP = green fluorescent protein, WT = wild-type, CD = catalytically-dead, FLAG = FLAG epitope, Tev = tev protease recognition site, Strep = streptavidin epitope, FL = full-length, UBL2 = ubiquitin-like domain 2, N = N-terminal 308 amino-acids of USP4, T = tumb domain, F = fingers domain, I = insert domain, P = palm domain, FC is C-terminal part of fingers domain, FN = N-terminal part of fingers domain, HA-Ub = hemagglutinin epitope tagged ubiquitin.

[c]Amino-acid substitutions are described as one letter abbreviations in column one and three letter abbreviations in column three and four.

Table S4 (Related to Figure 5): Ubiquitin Sites Identified on USP4 WT or CD by Tandem Mass Spectrometry

USP4 CD	USP4 WT	USP4 CD	USP4 WT
Lys-47	ND	Ser-315	Ser-315
Ser-72	Ser-72	ND	Thr-323
ND	Lys-132	ND	Thr-458
Lys-158	ND	Lys-588	ND
Thr-161	Thr161	Ser-589	ND
Lys-167	ND	Thr-594	ND
Lys-171	ND	Ser-714	Ser-714
**Lys-186**	ND	Lys-770	ND
Thr-201	ND	Lys-773	ND
Ser-231	ND	ND	**Lys-811**
Lys-232	ND	**Lys-837**	ND
Ser-247	ND	Ser-900	ND
ND	Ser-256	Thr-943	Thr-943

[a]USP4 ubiquitylations identified by mass spectrometry of GFP antibody immunoprecipitations from HEK293FT lysates, which do not include the cysteine ubiquitylations described in Figure S5C and S5D.

[b]Amino-acid three letter abbreviations of ubiquitylation sites detected on USP4 WT and CD are indicated. ND = not detected. Bold lettering indicates previously reported human USP4 ubiquitylation (Kim et al., 2011; Mertins et al., 2013; Wagner et al., 2011).

Supplemental Experimental Procedures

Cell Lines and Cell Culture

Human osteosarcoma U2OS cells were cultured at 37 °C in a humidified atmosphere and 5% (v/v) CO_2 in Dulbecco's modified eagle's medium (DMEM; Sigma-Aldrich) supplemented with 10% (v/v) fetal bovine serum (FBS; Life Technologies), 100 U/ml penicillin, 100 μg/ml streptomycin and 292 μg/ml L-glutamine (Life Technologies). SV40 virus T-antigen containing human embryonic kidney (HEK) 293FT cells or U2OS cells stably expressing GFP, GFP-USP4 WT(L), WT(H), CD, WT-C464A, CD-C464A, GFP-Flag-MRE11, FLAG-Tev-Strep-USP4 WT and CD were cultured in the presence of 0.5 mg/ml geneticin (Life Technologies). U2OS cells stably expressing direct-repeat (DR)-GFP were cultured in the presence of 1 μg/ml puromycin (Sigma-Aldrich) and were established according to a previously described method (Pierce et al., 1999).

Plasmids

All expression plasmids are listed in Table S3 and all oligo-nucleotides used to produce mutants of various genes in this study, were provided by Eurofins MWG Operon. GFP-fused USP4 WT (NM_003363), USP15 WT (NP_006304.1) and USP11 WT (NM_004651), were cloned in pEGFP-GW-JJ and were described previously (Nishi et al., 2014). Catalytically-dead mutants of USP4, USP15 and USP11 and zinc-binding domain mutants of USP4 and USP15 were generated by QuickChange Lightning site-directed mutagenesis according to the manufacturer's instructions (Agilent Technologiexs). MRE11 (NM_005590) was previously cloned in pEGFP-C1-FlagN by NotI-XmaI (NEB) digestion and ligation. Construction of pcDNA3.1-FLAG-Tev-Strep was done in two steps: First, fragment A, created by annealing oligo-nucleotide one (5'-CTA GCA CCA TGG ACT ACA AAG ACC ACG ACG GAG ACT ACA AAG ACC A-3') and two (5'-AGC TTT TTT GTT GGT ATT GCT AGT TCT CCT TGG AAG TAT AGG TTT T-3') was cloned into pcDNA3.1 by Nhe1-HindIII (NEB) digestion and ligation. Fragment B, created by annealing oligo-nucleotide three (5'-AGC TCG AAA ACC TAT ACT TCC AAA GCA GCG CAT GGA GCC ACC CAC A-3') and four 5'-GAT CCT TTT TCG AAT TGT GGG TGG CTC CAG CTT CCT CCT CCG CTT C-3') was cloned into pcDNA3.1-fragment A, by HindIII-BamHI (NEB) digestion and ligation. USP4 was sub-cloned into the pcDNA3.1-FLAG-Tev-Strep plasmid by BamHI-XhoI (NEB) digestion and ligation where pEGFP-GW-JJ-USP4 was used as the DNA template. Full length (FL) USP4 and the deletion mutants used in this study were cloned into pEGFP-C1-FLAGN after KpnI-BamHI digestion and ligation. The GFP-CtIP plasmid used in this study was described previously (Sartori et al., 2007). The HA-ubiquitin PCR product from oligonucleotide combination 5'-GGG GAT CCT CAA CCA CCT CTT AGT CTT AAG ACA-3' and 5'-GGG CTA GCA TGT ACC CAT ACG ATG TTC CAG ATT ACG CTC ATA TGC AGA TCT TCG TCA AGA CGT TAA-3' was cloned into pcDNA3.1 by BamHI and NheI (NEB) digestion and ligation.

siRNA Treatment

U2OS cells (4.8 x 10^5 cells/6 cm dish) were transfected with 30 nM siRNA (see Table S1 for siRNAs used in this study) using HiPerFect (Qiagen) transfection reagent according to the manufacturer's instruction, followed 24 hours later by a second identical transfection. Treatments and analyses were performed 72 hours after initial transfection.

Cell Extract Preparation, SDS-PAGE and Western Blot Analysis
Cells were lysed at 4 °C for 30 minutes with Cytoskeleton (CSK)-buffer [300 mM sucrose (Sigma), 3 mM $MgCl_2$ (Sigma), 10 mM piperazine-N,N′-bis(2-ethanesulfonic acid) [PIPES; pH 6.8 (Sigma)], 1 mM ethylene glycol tetra-acetic acid (EGTA), 0.1% Triton X-100 (Sigma), 1x EDTA free protease inhibitor cocktail (Roche), 1x phosphatase inhibitor cocktail (Sigma), 250 μM phenylmethylsulfonyl fluoride (PMSF; Sigma) and 10 mM N-Ethylmaleimide (NEM; Sigma)] containing 300 mM NaCl (Sigma). Soluble and chromatin fractions were separated by centrifugation (20,000 x g for 10 minutes at 4 °C). Chromatin fractions were sonicated at 4 °C (30% amplitude; Sonics Vibra Cell, VHX 500 Watt) and protein concentrations were determined by Bradford protein assays (Thermo Scientific). Ten micrograms of soluble or the corresponding amount of chromatin fractions were boiled for five minutes at 95 °C in loading-buffer [67 mM Tris/HCl (pH 6.8), 2% (w/v) SDS (MP Biomedicals). 10% (v/v) Glycerol (Sigma) and 0.002% (w/v) Bromophenol Blue (Sigma) and 20% (v/v) β−mercaptoethanol (βME) in milliQ water], loaded on 4-12% Bis/Tris (Invitrogen) or Tris acrylamide gels [375 mM Tris (pH 8.8), various acrylamide/bis-acrylamide (37.5:1; Geneflow) concentrations, 0.1% (m/v) SDS, 1% (m/v) ammonium persulfate (APS) and 0.1% (v/v) tetramethylethylenediamine (TEMED)] and separated at 120 V for two hours with NuPAGE® MOPS SDS Running Buffer (Invitrogen) or Tris/glycine buffer [25 mM Tris, 191 mM glycine (Sigma) and 0.1% (m/v) SDS], respectively. After separation the proteins were transferred (350 mA, 1.5 h) onto nitro-cellulose membranes (Millipore), using the Biorad blotting systems according to the manufacturer's instructions, in Tris/Glycine buffer containing 10% (v/v) Methanol (Normapur). Nitrocellulose membranes were then blocked in Tris-buffered saline containing 0.1% (v/v) Tween-20 (TBS-T; Sigma-Aldrich) containing 1% (w/v) bovine serum albumin (BSA; Sigma-Aldrich) and incubated with various primary and secondary antibodies summarized in Table S2.

Neutral Comet Assay
U2OS cells were treated with phleomycin (40 μg/ml; Sigma) for two hours. Cells were then washed twice with phosphate buffered saline (PBS) and left to recover for two hours under normal cell culture conditions. Cells were then washed once with PBS [pH 7.5, lacking $CaCl_2$ and $MgCl_2$ (-/-); Life Technologies] and scraped off in 500 μl PBS (-/-), after which the pellets were re-suspended in PBS (-/-) at an approximate concentration of 5×10^6 cells/ml. Cell suspensions (10 μl) were mixed with 90 μl LMAgarose (37 °C; Trevigen) and 70 μl was spotted onto GelBond Films (Lonza), covered with 22 mm cover glasses (VWR) and incubated at 4 °C for 10 minutes. Cover glasses were removed and samples were incubated in Trevigen lysis solution for one hour at 4 °C. The lysis solution was then washed off with TBE [90 mM Tris-Borate, pH 8.3 and 2 mM 2,2',2'',2'''-(Ethane-1,2-diyldinitrilo)tetraacetic acid (EDTA)] and samples were exposed to a 35 V current for seven minutes in TBE at 22 °C. After fixation in 70% (v/v) ethanol for five minutes at 22 °C, the samples were dried for approximately 16 hours and stained with SYBER green nucleic acid staining solution (Invitrogen). Pictures were taken with an inverted Olympus TH4-200 microscope, connected to a Lumen2000 Prior stage, a FView soft imaging camera and Cell^F analysis imaging software. Per condition, the tail moments of approximately 50 individual cells were quantified with CometScore software (Tritek Corp.). Tail moments – product of tail length and fraction of total DNA in tails – of recovered cells were normalized to tail moments of damaged cells that were run on the same gel bond. Tail moments of undamaged cells were used to ascertain damage had occurred.

Clonogenic Cell Survival Assay
Cells were seeded, in six well plates (NUNC) at different concentrations (250, 500 or 1000 cells/well), 48 hours after the first siRNA treatment. Twenty-four hours after seeding, cells were exposed to various acute doses of ionizing radiation (Faxitron X-Ray Corporation, Illinois, USA), or camptothecin (Sigma-Aldrich) for one hour (camptothecin was removed and the cells were washed twice with PBS). Cells were cultured for 10 to 14 days and then stained with crystal violet solution [2% (w/v) crystal violet in 10% (v/v) ethanol]. Colonies containing more than 30 cells were counted and normalized to the undamaged controls.

Live Cell Laser-Line Micro-Irradiation
U2OS cells (1.0 x 10^5 cells/dish) stably expressing GFP-USP4 WT(H) were seeded in glass-bottom dishes. Twenty-four hours later, cells were pre-sensitized with 10 μM BrdU for 24 hours and then subjected to 400 μW localized laser micro-irradiation with a 405 nm UV-A laser beam (Limoli et al., 1993). Pictures were taken before and 30 minutes after irradiation.

Immunoprecipitation
U2OS or 293FT cells, which were cultured in 15 cm dishes (NUNC) and in most cases transfected with 15 μg of various GFP-fused expression vectors (summarized in Table S3), were washed twice with PBS and re-suspended in 1 ml (U2OS) or 3 ml (HEK293FT) lysis buffer [50 mM Tris-HCl (pH 7.5), 100 mM NaCl, 1 mM $MgCl_2$, 10% (v/v) glycerol, 5 mM NaF, 0.2% Igepal CA-630 (Sigma), protease inhibitor cocktail (10x) and 10 mM NEM] containing 25 U/ml benzonase (Novagen). Lysates were incubated at 4 °C for 45 minutes whilst rotating (15 rpm, 20 cm diameter). Then, NaCl and EDTA were added to a final concentration of 200 mM and 2 mM, respectively and lysates were incubated for an extra 20 minutes at 4 °C (whilst rotating). Lysates were subsequently separated by centrifugation (20,000 x g for 10 minutes at 4 °C).

To immunoprecipitate USP4, supernatants from 293FT cells were incubated with 1 μg/mg USP4 antibody (Bethyl) or non-specific rabbit immunoglobulins (IgG) for 16 hours at 4 °C. Supernatants were subsequently incubated in the presence of 30 μl protein A Dynabeads (Life Technologies) for one hour at 4 °C and then washed six times with lysis buffer containing 200 mM NaCl and 2 mM EDTA. Beads were subsequently incubated in 1x loading buffer (diluted with lysis buffer) at 95 °C for 10 minutes and then subjected to western blot analysis as described above.

Supernatants from cells that were transfected with various GFP-fused expression vectors were immunoprecipitated using 10 μl/sample GFP-Trap®_A gta-20 beads (Cromotek) for two and up to 16 hours at 4 °C and then washed and processed as described above.

DR-GFP HR Reporter Assay
The homologous recombination (HR) repair assays using U2OS cells, stably expressing the direct-repeat (DR)-GFP reporter, were carried out based on a previously established methodology (Pierce et al., 1999). DR-GFP cells, seeded in 6 cm dishes (4.8 x 10^5 cells/dish) and treated with siRNAs for 72 hours, were transfected with 4 μg of different plasmid combinations as described below, using the TransIT-LT1 transfection reagent according to the manufacturer's protocol (Mirus Bio LLC). To induce DSBs, the restriction enzyme I-SceI was transiently expressed (3.75 μg per sample pCBA I-SceI) together with

red fluorescent protein (RFP; 0.25 μg per sample pCS2-mRFP) to control for the transfection efficiency. To control for any background GFP signal a combination of the empty pCDNA3.1 plasmids and pCS2-mRFP was transfected (3.75 μg and 0.25 μg respectively). Forty-eight hours after plasmid transfection the cells were washed twice with PBS, detached from their growth plates using 0.1% EDTA in PBS, and collected in PBS with 5% (w/v) FBS. To be able to exclude dead cells, 1 μg/ml 4',6-diamidino-2-phenylindole (DAPI) was added, and the HR efficiency was measured as the amount of GFP and RFP double positive cells normalized to the background-GFP control population, with the BD LSRFortessa cell analyzer.

DNA-End Resection (BrdU) Assay

The DNA-end resection assays were carried out based on a previously established methodology (Nishi et al., 2014). Forty-eight hours after the first siRNA treatments, U2OS cells were pulse-labelled with 30 μM BrdU for 24 hours and then treated with 1 μM camptothecin for one hour. In some cases, cells were washed twice with PBS and then left to recover under normal cell culture conditions for one hour. After camptothecin treatment cells were washed twice with PBS, scraped off and re-suspended in 500 μl PBS. Cell suspensions were fixed in ice cold 70% (v/v) ethanol and stored at -20 °C for a minimum of 16 hours. Fixed cells were washed twice with PBS-T, incubated in block-buffer (30 minutes, 22 °C) and incubated with anti-BrdU and anti-γH2AX primary antibodies and different combinations of secondary antibodies in block-buffer (summarized in Table S2), for two and one hour at 22 °C, respectively. After antibody treatments the samples were analyzed with the BD LSRFortessa cell analyzer (BD Biosciences) in the presence of 1 μg/ml DAPI.

Cell Cycle Analysis

U2OS cells were pulse-labelled with 10 μM BrdU for 30 minutes, washed twice with PBS and scraped off in 500 μl PBS. Cell suspensions were fixed in ice cold 70% (v/v) ethanol and stored for a minimum of 16 hours at -20 °C. Fixed cells were washed twice with PBS and pellets were re-suspended in 400 μl acid-solution [5 M HCl and 0.1% (v/v) Triton X-100 in MilliQ water]. After 20 minutes incubation at 22 °C, the acid was neutralized with $Na_2B_4O_7$ (4 ml, 0.1 M). Pellets were washed once with PBS-T, incubated in block-buffer for 30 minutes at 22 °C, incubated with an anti-BrdU and a secondary Alexa Fluor® 488 goat anti-mouse antibody (summarized in Table S2). The pellets were then incubated in 300 μl PI-reagent [10 μg/ml propidium iodide (Invitrogen) and 250 μg/ml RNase A (Invitrogen)] for 20 minutes at 37 °C and cells were profiled with the BD LSRFortessa cell analyzer.

Random Plasmid Integration Assay

Seventy-two hours after the initial siRNA transfection, U2OS cells were transfected with 5 μg (per 6 cm dish) linearized (by BamHI and XhoI restriction digestion) pEGFP-C1 plasmids with the TransIT-LT1 transfection reagent according to the manufacturer's instructions. Six hours after plasmid transfection, cells were seeded into 15 cm dishes at four different concentrations (1.0×10^3, 2.5×10^3, 1.0×10^4 or 2.0×10^4 cells/plate). Twenty-four hours after re-seeding, plates containing 1.0×10^4 or 2.0×10^4 cells were cultured in presence of 1 mg/ml of geneticin and the plates containing 1.0×10^3 or 2.5×10^3 cells in absence of geneticin for 10 to 14 days and subsequently stained with 2% (w/v) crystal violet solution. Plasmid integration efficiencies were analyzed as the percentage of geneticin-resistant cells normalized to the transfection efficiency [transfection efficiencies

were calculated as percentages of GFP positive cells (24 hours after plasmid transfection), assessed by flow-cytometry with the FACS Calibur (BD Biosciences)].

Immunofluorescence Staining

U2OS cells, cultured on poly-L-lysine [0.01% (w/v); Sigma-Aldrich] coated coverslips or glass bottom dishes (Willco Wells; 3.5 cm diameter and 0.17 mm glass thickness), were fixed with 2% (w/v) paraformaldehyde (PFA; Sigma-Aldrich) at 22 °C for 20 minutes and permeabilized with 0.2% (v/v) Triton X-100 at 22 °C for 15 minutes. Following incubation in block-buffer [PBS, 5% (v/v) FBS] for 30 minutes at 22 °C, fixed cells were incubated with various primary antibodies at 4 °C for 16 to 24 hours. Cells were washed twice with PBS containing 0.1% (v/v) Tween-20 (PBS-T) and incubated with various secondary antibodies at 22 °C for one to two hours. Primary and secondary antibodies are described in Table S2. Last, cells were exposed to 1 μg/ml DAPI in PBS for 10 minutes (22 °C), mounted onto glass and subsequently analyzed with an Olympus inverted confocal microscope.

EdU Labeling

Seventy-two hours after the first transfection, U2OS cells were exposed to 10 μM 5-ehtynyl-2'-deoxyuridine (EdU) for 15 minutes. Cells were then washed twice with PBS and fixed in 2% PFA in PBS (w/v) at 22 °C for 20 minutes. Subsequently, the cells were washed twice in PBS and permeabilized with 0.2% Triton-X-100 in PBS (15 minutes at 22 °C), washed once with PBS and incubated in 100 μl/sample 'Click-iT' solution [20 mg/ml (1x) Click-iT® EdU buffer, 20 mM $CuSO_4$ and 1:500 diluted Alexa Fluor 647 azide in PBS (Molecular probes)] for 30 minutes at 22 °C, under protection from the light. The EdU intensities of the S-phase cell populations were measured with the BD LSRFortessa cell analyzer.

NBS1 or CtIP Recruitment to Laser-Line Micro-Irradiation Induced DNA Lesions

Cells (1.0 x 10^5 cells/dish) were seeded in glass bottom dishes. Forty-eight hours after siRNA treatments cells were pre-sensitized with 10 μM BrdU for 24 hours and then subjected to 250 μW localized laser micro-irradiation with a 405 nm UV-A laser beam (Limoli et al., 1993). Two hours after the first cells were irradiated, the samples were processed for immunofluorescent staining as described above.

Ubiquitylation Assay

Cells were cultured in 10 cm or 15 cm dishes and transfected with 2.5 μg or 7.5 μg pcDNA3.1-HA-ubiquitin and 2.5 μg or 7.5 μg various expression vectors (summarized in Table S3), respectively. Forty-eight hours later, cells were washed twice with PBS and the pellets were lysed with CSK buffer containing 300 mM NaCl (described previously but without addition of phosphatase inhibitor or PMSF). Supernatants were immunoprecipitated using EZview™ RED-Anti-HA affinity beads (Sigma) for two hours and washed six times with CSK buffer. Beads were then incubated at 95 °C for 10 minutes in loading buffer either in presence or absence of βME and subjected to western blot analysis as described above.

Sample Preparation for Mass Spectrometry

Immunoprecipitated and eluted protein materials were precipitated and desalted using chloroform-methanol. Protein samples were reduced and alkylated using 20 mM DTT and 20 mM iodoacetamide as described previously (Ternette et al., 2013). Tryptic digests were

carried out overnight at 37 °C and desalting steps were performed with C18 SepPak cartridge columns according to the manufacturer's instructions (Waters). Eluted peptides were dried under vacuum and the pellets were kept at -20 °C until analysis. Pellets were re-suspended in 20 μl H_2O with 2% (v/v) acetonitrile, 0.1% formic acid (v/v) prior mass spectrometry analysis.

Tandem Mass Spectrometry and Data Analysis

Samples were analyzed with nano–liquid chromatography tandem mass spectrometry (nano–LC-MS/MS), using a Dionex Ultimate 3000 UPLC coupled to a hybrid quadrupole-orbitrap instrument (Q Exactive, Thermo). Samples were loaded onto a PepMAP C18, 300 μm x 5mm, 5 μm particle pre-column (Thermo) for one minute at a flow rate of 20 μl/min and separated on an nEASY column (PepMAP C18, 75 μm x 500 mm, 2 μm particle; Thermo) for 60 minutes using a gradient of 2%-35% acetonitrile (v/v) in 5% DMSO (v/v) and 0.1% formic acid (v/v) at 250 nl/min. Scans were performed at a resolution of 70,000 at 200 mass/charge and the 15 most abundant precursors were selected for Higher-energy Collisional Dissociation (HCD) fragmentation.

Peak lists containing MS/MS spectra were generated using MSConvert (ProteoWizard 3.0.4743), keeping the 200 most intense peaks. These lists were then searched using Mascot version 2.5.1 (http://www.matrixscience.com) against the Swiss-Prot protein database with the taxonomy restriction "human" (20,353 entries as of July 2014) with tryptic enzymatic restriction and with mass deviations of 10 parts per million/0.05 daltons in MS and MS/MS mode, respectively. Oxidation of methionine, deamidation of asparagine and glutamine, and GlyGly (C/T/S/K) were searched for as variable modifications. In addition, the same data sets were analyzed using PEAKS (Version 7, Bioinformatics Solutions) software. Raw MS data were *de novo* sequenced by PEAKS using HCD fragmentation data. A database search (SwissProt, 20,204 human sequences) with subsequent posttranslational modification (PTM) searches, where all modifications reported in UNIMOD were considered, was then applied to the *de novo* identified MS/MS spectra. False discovery rates (FDR) of 1% threshold were applied. Peptide MS/MS spectra including GlyGly modifications were inspected manually.

Biotin-Ubiquitin Binding Assay

Cells (293FT) were cultured in 15 cm dishes and transfected with 15 μg plasmid [GFP or GFP-fused USP4 derivatives (summarized in Table S3)]. Forty-eight hours later, cells were processed to create protein extracts using CSK buffer containing 300 mM NaCl and without phosphatase inhibitor, PMSF or NEM, according to the protocol described above. Meanwhile, streptavidin M-280 Dynabeads (Life Technologies) were soaked in an excess of Biotin-fused human recombinant ubiquitin (R&D Systems) for one hour at 4 ºC, after which 10 μl ubiquitin-coupled streptavidin was added to the lysates, incubated for 16 hours at 4 ºC, washed six times in CSK buffer containing 300 mM NaCl, incubated at 95 °C for 10 minutes in loading buffer and subjected to western blot analysis as described previously.

Active Probe Binding Assay

Cell extracts were lysed (30 min at 4 ºC) with NP lysis buffer [50 mM Tris-HCl (pH 7.5), 150 mM NaCl, 3 mM EDTA and 0.5% NP-40] containing 1x EDTA free protease inhibitor, 1x phosphatase inhibitor, 1 mM DTT. Supernatants were prepared by centrifugation at

20,000x g for 10 min at 4 ºC. To 20 μg supernatant, 250 ng HA-Ub-VS probe [vinyl sulfone, (HA-tag); Enzo Life Sciences, (Borodovsky et al., 2002)] was added and incubated at 37 ºC for two hours. Samples were boiled for 10 minutes at 95 °C in loading-buffer to inactivate the reaction and prepare for SDS-PAGE and western blot analysis as described previously.

Supplemental References

Borodovsky, A., Ovaa, H., Kolli, N., Gan-Erdene, T., Wilkinson, K.D., Ploegh, H.L., and Kessler, B.M. (2002). Chemistry-based functional proteomics reveals novel members of the deubiquitinating enzyme family. Chem Biol *9*, 1149–1159.

Kim, W., Bennett, E.J., Huttlin, E.L., Guo, A., Li, J., Possemato, A., Sowa, M.E., Rad, R., Rush, J., Comb, M.J., et al. (2011). Systematic and quantitative assessment of the ubiquitin-modified proteome. Molecular Cell *44*, 325–340.

Larrieu, D., Britton, S., Demir, M., Rodriguez, R., and Jackson, S.P. (2014). Chemical inhibition of NAT10 corrects defects of laminopathic cells. Science *344*, 527–532.

Limoli, C.L., Limoli, C.L., Ward, J.F., and Ward, J.F. (1993). A new method for introducing double-strand breaks into cellular DNA. Radiat. Res. *134*, 160–169.

Mertins, P., Qiao, J.W., Patel, J., Udeshi, N.D., Clauser, K.R., Mani, D.R., Burgess, M.W., Gillette, M.A., Jaffe, J.D., and Carr, S.A. (2013). Integrated proteomic analysis of post-translational modifications by serial enrichment. Nat. Methods *10*, 634–637.

Nishi, R., Wijnhoven, P., le Sage, C., Tjeertes, J., Galanty, Y., Forment, J.V., Clague, M.J., Urbé, S., and Jackson, S.P. (2014). Systematic characterization of deubiquitylating enzymes for roles in maintaining genome integrity. Nat. Cell Biol. *16*, 1016–26–1–8.

Pierce, A.J., Johnson, R.D., Thompson, L.H., and Jasin, M. (1999). XRCC3 promotes homology-directed repair of DNA damage in mammalian cells. Genes & Development *13*, 2633–2638.

Riballo, E., Critchlow, S.E., Teo, S.H., Doherty, A.J., Priestley, A., Broughton, B., Kysela, B., Beamish, H., Plowman, N., Arlett, C.F., et al. (1999). Identification of a defect in DNA ligase IV in a radiosensitive leukaemia patient. Curr Biol *9*, 699–702.

Sartori, A.A., Lukas, C., Coates, J., Mistrik, M., Fu, S., Bartek, J., Baer, R., Lukas, J., and Jackson, S.P. (2007). Human CtIP promotes DNA end resection. Nature *450*, 509–514.

Ternette, N., Yang, M., Laroyia, M., Kitagawa, M., O'Flaherty, L., Wolhulter, K., Igarashi, K., Saito, K., Kato, K., Fischer, R., et al. (2013). Inhibition of mitochondrial aconitase by succination in fumarate hydratase deficiency. Cell Reports *3*, 689–700.

Wagner, S.A., Beli, P., Weinert, B.T., Nielsen, M.L., Cox, J., Mann, M., and Choudhary, C. (2011). A proteome-wide, quantitative survey of in vivo ubiquitylation sites reveals widespread regulatory roles. Mol. Cell Proteomics *10*, M111.013284.

Yu, X., and Baer, R. (2000). Nuclear localization and cell cycle-specific expression of CtIP, a protein that associates with the BRCA1 tumor suppressor. J Biol Chem *275*, 18541–18549.
